# The leucine-rich repeat receptor kinase QSK1 regulates PRR-RBOHD complexes targeted by the bacterial effector HopF2*_Pto_*

**DOI:** 10.1093/plcell/koae267

**Published:** 2024-10-21

**Authors:** Yukihisa Goto, Yasuhiro Kadota, Malick Mbengue, Jennifer D Lewis, Hidenori Matsui, Noriko Maki, Bruno Pok Man Ngou, Jan Sklenar, Paul Derbyshire, Arisa Shibata, Yasunori Ichihashi, David S Guttman, Hirofumi Nakagami, Takamasa Suzuki, Frank L H Menke, Silke Robatzek, Darrell Desveaux, Cyril Zipfel, Ken Shirasu

**Affiliations:** Plant Immunity Research Group, RIKEN Center for Sustainable Resource Science (CSRS), Yokohama, Kanagawa 230-0045, Japan; Graduate School of Science, The University of Tokyo, Tokyo 113-8654, Japan; Institute of Plant and Microbial Biology, Zurich-Basel Plant Science Center, University of Zurich, Zurich CH-8008, Switzerland; Plant Immunity Research Group, RIKEN Center for Sustainable Resource Science (CSRS), Yokohama, Kanagawa 230-0045, Japan; The Sainsbury Laboratory, University of East Anglia, Norwich Research Park, Norwich NR4 7UH, UK; Laboratoire de Recherche en Sciences Végétales, Université de Toulouse, CNRS, UPS, Toulouse INP, Castanet-Tolosan 31326, France; Department of Cell and System Biology, Centre for the Analysis of Genome Function and Evolution, University of Toronto, Toronto, ON, Canada M5S 3B2; Plant Gene Expression, United States Department of Agriculture, Agricultural Research Service, Albany, CA 94710, USA; Department of Plant and Microbial Biology, University of California Berkeley, Berkeley, CA 94720, USA; Plant Proteomics Research Unit, RIKEN CSRS, Yokohama 230-0045, Japan; Graduate School of Environmental and Life Science, Okayama University, Okayama 700-8530, Japan; Plant Immunity Research Group, RIKEN Center for Sustainable Resource Science (CSRS), Yokohama, Kanagawa 230-0045, Japan; Plant Immunity Research Group, RIKEN Center for Sustainable Resource Science (CSRS), Yokohama, Kanagawa 230-0045, Japan; The Sainsbury Laboratory, University of East Anglia, Norwich Research Park, Norwich NR4 7UH, UK; The Sainsbury Laboratory, University of East Anglia, Norwich Research Park, Norwich NR4 7UH, UK; Plant Immunity Research Group, RIKEN Center for Sustainable Resource Science (CSRS), Yokohama, Kanagawa 230-0045, Japan; Plant Immunity Research Group, RIKEN Center for Sustainable Resource Science (CSRS), Yokohama, Kanagawa 230-0045, Japan; Plant-Microbe Symbiosis Research and Development Team, RIKEN BioResource Research Center, Tsukuba, Ibaraki 305-0074, Japan; Department of Cell and System Biology, Centre for the Analysis of Genome Function and Evolution, University of Toronto, Toronto, ON, Canada M5S 3B2; Plant Proteomics Research Unit, RIKEN CSRS, Yokohama 230-0045, Japan; Protein Mass Spectrometry, Max Planck Institute for Plant Breeding Research, Cologne 50829, Germany; College of Bioscience and Biotechnology, Chubu University, Kasugai 487-0027, Japan; The Sainsbury Laboratory, University of East Anglia, Norwich Research Park, Norwich NR4 7UH, UK; The Sainsbury Laboratory, University of East Anglia, Norwich Research Park, Norwich NR4 7UH, UK; LMU Biocentre, Ludwig-Maximilian-University of Munich, 82152 Martinsried, Germany; Department of Cell and System Biology, Centre for the Analysis of Genome Function and Evolution, University of Toronto, Toronto, ON, Canada M5S 3B2; Institute of Plant and Microbial Biology, Zurich-Basel Plant Science Center, University of Zurich, Zurich CH-8008, Switzerland; The Sainsbury Laboratory, University of East Anglia, Norwich Research Park, Norwich NR4 7UH, UK; Plant Immunity Research Group, RIKEN Center for Sustainable Resource Science (CSRS), Yokohama, Kanagawa 230-0045, Japan; Graduate School of Science, The University of Tokyo, Tokyo 113-8654, Japan

## Abstract

Plants detect pathogens using cell-surface pattern recognition receptors (PRRs) such as ELONGATION Factor-TU (EF-TU) RECEPTOR (EFR) and FLAGELLIN SENSING 2 (FLS2), which recognize bacterial EF-Tu and flagellin, respectively. These PRRs belong to the leucine-rich repeat receptor kinase (LRR-RK) family and activate the production of reactive oxygen species via the NADPH oxidase RESPIRATORY BURST OXIDASE HOMOLOG D (RBOHD). The PRR-RBOHD complex is tightly regulated to prevent unwarranted or exaggerated immune responses. However, certain pathogen effectors can subvert these regulatory mechanisms, thereby suppressing plant immunity. To elucidate the intricate dynamics of the PRR-RBOHD complex, we conducted a comparative coimmunoprecipitation analysis using EFR, FLS2, and RBOHD in *Arabidopsis thaliana*. We identified QIAN SHOU KINASE 1 (QSK1), an LRR-RK, as a PRR-RBOHD complex-associated protein. QSK1 downregulated FLS2 and EFR abundance, functioning as a negative regulator of PRR-triggered immunity (PTI). QSK1 was targeted by the bacterial effector HopF2*_Pto_*, a mono-ADP ribosyltransferase, reducing FLS2 and EFR levels through both transcriptional and transcription-independent pathways, thereby inhibiting PTI. Furthermore, HopF2*_Pto_* transcriptionally downregulated *PROSCOOP* genes encoding important stress-regulated phytocytokines and their receptor MALE DISCOVERER 1-INTERACTING RECEPTOR-LIKE KINASE 2. Importantly, HopF2*_Pto_* requires QSK1 for its accumulation and virulence functions within plants. In summary, our results provide insights into the mechanism by which HopF2*_Pto_* employs QSK1 to desensitize plants to pathogen attack.

## Teaser

A bacterial effector exploits a plant receptor kinase, a component of immune receptor complexes, and reduces immune receptor accumulation to desensitize plants to pathogen attack.

## Introduction

Plants and pathogens are in a perpetual evolutionary arms race. A fundamental aspect of the plant's defense mechanism lies in its capability to detect microbial molecules, particularly pathogen-associated molecular patterns (PAMPs) as well as endogenous danger molecules that are released from damaged or dying cells, known as damage-associated molecular patterns (DAMPs). These PAMPs and DAMPs are recognized by specialized cell-surface receptors known as pattern recognition receptors (PRRs) (Macho and Zipfel 2014; DeFalco and Zipfel 2021). Among those, leucine-rich repeat receptor kinases (LRR-RKs) play a central role in the recognition of PAMPs and DAMPs. For instance, ELONGATION Factor-TU (EF-Tu) RECEPTOR (EFR) and FLAGELLIN SENSING 2 (FLS2) detect bacterial EF-Tu and flagellin, respectively. The binding of flg22 or elf18 (the immunogenic peptides of flagellin or EF-Tu, respectively) to FLS2 and EFR induces their instant association with the coreceptor LRR-RK BRI1-ASSOCIATED RECEPTOR KINASE 1 (BAK1) and concomitant phosphorylation of both proteins to initiate PRR-triggered immunity (PTI) (Chinchilla et al. 2007; Heese et al. 2007; Roux et al. 2011). Subsequently, the PRR-BAK1 complex activates receptor-like cytoplasmic kinases such as BOTRYTIS-INDUCED KINASE 1 (BIK1) by phosphorylation (Lu et al. 2010; Zhang et al. 2010; Liu et al. 2013). PRRs further form a complex with the NADPH oxidase RESPIRATORY BURST OXIDASE HOMOLOG D (RBOHD), which is phosphorylated by activated BIK1, resulting in the rapid generation of reactive oxygen species (ROS) (Kadota et al. 2014, 2015; Li et al. 2014). In addition, phosphorylated BIK1 activates Ca^2+^ channels, including REDUCED HYPEROSMOLALITY-INDUCED [Ca^2+^] INCREASE 1.3 (OSCA1.3), CYCLIC NUCLEOTIDE-GATED CHANNEL 2 (CNGC2), and CYCLIC NUCLEOTIDE-GATED CHANNEL 4 (CNGC4), particularly under specific Ca^2+^ concentrations (Tian et al. 2019; Thor et al. 2020). This activation leads to an increase in cytoplasmic Ca^2+^ concentration, subsequently stimulating Ca^2+^-dependent protein kinases (Boudsocq et al. 2010). Furthermore, BIK1 phosphorylates the noncanonical Gα protein, EXTRA LARGE G-PROTEIN 2, facilitating its translocation to the nucleus. This phenomenon inhibits MUT9-like kinases, thereby removing the negative regulation of PTI (Liang et al. 2016; Ma et al. 2022).

The activity of PRR complex is negatively regulated by various proteins, such as protein phosphatases and LRR-RKs. PROTEIN PHOSPHATASE 2A (PP2A) constitutively associates with BAK1, keeping it dephosphorylated and inactive until PAMP perception (Segonzac et al. 2014). Similarly, in the absence of PAMPs, BIK1 and BAK1 are inactivated by PP2C38 and PP2Cs, POLTERGEIST-LIKE 4 and 5 (PLL4 and PLL5), respectively (Couto et al. 2016; DeFalco et al. 2022). Upon PRR activation by PAMPs, BIK1 phosphorylates PP2C38 and PLL4/5, causing them to dissociate from the PRR complex. The pseudokinase LRR-RKs, BAK1-INTERACTING RECEPTOR-LIKE KINASE 2 (BIR2), and BIR3 interact with BAK1 to inhibit the formation of the FLS2-BAK1 complex (Halter et al. 2014; Imkampe et al. 2017; Ma et al. 2017). Similarly, the short LRR-RKs APEX (AT5G63710) and NUCLEAR SHUTTLE PROTEIN-INTERACTING KINASE 1 also negatively regulate FLS2-BAK1 interaction (Smakowska-Luzan et al. 2018; Li et al. 2019). This intricate coordination of signal transduction within PRR complexes allows plants to rapidly and effectively mount immune responses at the site of infection.

To overcome effective plant immunity, the pathogens deploy virulence effectors to target and dampen immune signaling components (Dou and Zhou 2012). Effectors with high immunomodulatory activities, especially those that suppress early PTI responses such as ROS production, MAPK activation, and Ca^2+^ influx, often target PRRs or their associated components. For example, AvrPto, a Type III effector from *Pseudomonas syringae*, directly inhibits the kinase activity of FLS2 and EFR (Xiang et al. 2008). AvrPtoB functions as an E3 ligase, catalyzing the polyubiquitination and degradation of FLS2, BAK1, and CHITIN ELICITOR RECEPTOR KINASE 1 (Goehre et al. 2008; Gimenez-Ibanez et al. 2009; Cheng et al. 2011). HopB1 associates with FLS2 and serves as a protease, cleaving activated BAK1 (Li et al. 2016). The *Xanthomonas campestris* effector AvrAC employs a unique uridylyl-transferase activity to impede the activation of BIK1 (Feng et al. 2012). These findings highlight the utility of effectors that suppress early PTI responses as valuable tools for identifying and confirming PRR complex components. Indeed, key regulators in the PRR complex, such as BIK1 and PBS1-LIKE kinases, were originally identified as targets of the bacterial effector AvrPphB, which possesses cysteine protease activity (Zhang et al. 2010). A comprehensive investigation of PRR complex components in conjunction with virulence effectors will shed light on the essential regulatory mechanisms governing PRR complexes and uncover how pathogens manipulate the PRR complex to enhance their virulence.

In this study, we used comparative immunoprecipitation (IP) analysis of EFR, FLS2, and RBOHD followed by MS (IP-MS) to identify components of mature PRR-RBOHD complexes situated at the plasma membrane. This investigation led to the identification of QIAN SHOU KINASE 1 (QSK1), an LRR-RK, protein associated with PRR-RBOHD complexes. Intriguingly, QSK1 plays a negative regulatory role in PTI, possibly by controlling the steady-state levels of PRRs. Our interaction assays further revealed an association between the bacterial effector HopF2*_Pto_* and QSK1. HopF2*_Pto_*, a mono-ADP ribosyltransferase, reduces PRR protein levels through both transcriptional and transcription-independent mechanisms. Moreover, HopF2*_Pto_* disrupts the signaling induced by SERINE RICH ENDOGENOUS PEPTIDE (SCOOP) phytocytokines. Importantly, the accumulation and virulence activities of HopF2*_Pto_* within plants rely on QSK1. In summary, our findings provide insights into the mechanisms by which QSK1 modulates PRR abundance and how HopF2*_Pto_* exploits QSK1 to render plant cells insensitive to PAMPs, DAMPs, and SCOOP phytocytokines.

## Results

### Identification of QSK1, a component of PRR-RBOHD complexes

**Figure 1.**
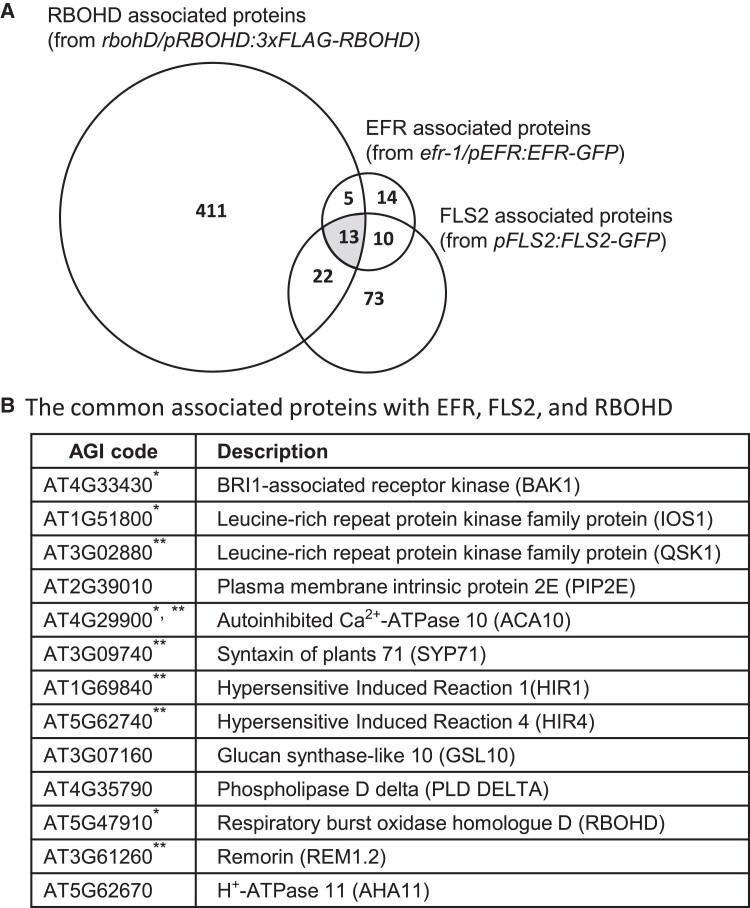
Commonly associated proteins with EFR, FLS2, and RBOHD in *A. thaliana.*  **A)** Comparison of candidate-associated proteins with EFR, FLS2, and RBOHD identified by co-IP. The Venn diagram illustrates candidate-associated proteins identified by IP of EFR-GFP, FLS2-GFP, or 3xFLAG-RBOHD from *Arabidopsis* seedlings of *efr-1/pEFR:EFR-GFP* (Kadota et al. 2014), *pFLS2:FLS2-GFP*, or *rbohD/pRBOHD:3×FLAG-gRBOHD* (Goto et al. 2024). The protein list is shown in Supplementary Data Set S1. **B)** The list of commonly associated proteins with EFR, FLS2, and RBOHD. An asterisk indicates the known components of the PRR complex, and the double asterisks indicate proteins accumulate in DRM compartments in response to flg22 (Keinath et al. 2010).

To isolate components specific to mature PRR-RBOHD complexes at the plasma membrane, we employed a comparative IP-MS strategy with EFR, FLS2, and RBOHD. Given the distinct protein structures of PRRs and RBOHD, it is likely that associated regulatory proteins involved in protein modification, maturation, transport, and degradation processes differ. Therefore, proteins that can associate with EFR, FLS2, and RBOHD are the most likely candidates to be associated with mature PRR-RBOHD complexes. To mitigate potential false positives resulting from sticky proteins, we implemented 2 different IP systems: magnetic and agarose beads. Through IP of FLS2-GFP from the *Arabidopsis* (*Arabidopsis thaliana*) *pFLS2:FLS2-GFP* line using anti-GFP magnetic beads, we identified 118 FLS2-associated candidates (Supplementary Data Set S1_1). We had previously performed an IP of EFR-GFP using anti-GFP magnetic beads from the *efr-1/pEFR:EFR-GFP* line, identifying 42 candidate EFR-associated proteins (Supplementary Data Set S1_2) (Kadota et al. 2014). Moreover, we previously identified 451 candidate RBOHD-associated proteins through IP of 3xFLAG-RBOHD from the *rbohD*/*pRBOHD:3xFLAG-RBOHD* line by using Anti-FLAG agarose and eluted 3xFLAG-RBOHD with free 3xFLAG peptides (Supplementary Data Set S1_3) (Goto et al. 2024). Venn diagram analysis of these candidates pinpointed 13 proteins commonly associated with FLS2, EFR, and RBOHD (Fig. 1; Supplementary Data Set S1_4), including known components of PRR complexes such as BAK1 (Chinchilla et al. 2007; Heese et al. 2007; Roux et al. 2011), IMPAIRED OOMYCETE SUSCEPTIBILITY 1 (IOS1) (Yeh et al. 2016), AUTOINHIBITED Ca^2+^-ATPASE 10 (ACA10) (Frei dit Frey et al. 2012), and RBOHD (Kadota et al. 2014; Li et al. 2014). Additionally, several proteins are known to accumulate in detergent-resistant membrane (DRM) compartments in response to flg22, including QSK1, ACA10, SYNTAXIN OF PLANTS 71 (SYP71), HYPERSENSITIVE INDUCED REACTION1 (HIR1), HIR4, and REMORIN 1.2 (REM1.2) (Keinath et al. 2010). These results validate the effectiveness of our comparative IP-MS approach for identifying members of mature PRR-RBOHD complexes.

**Figure 2.**
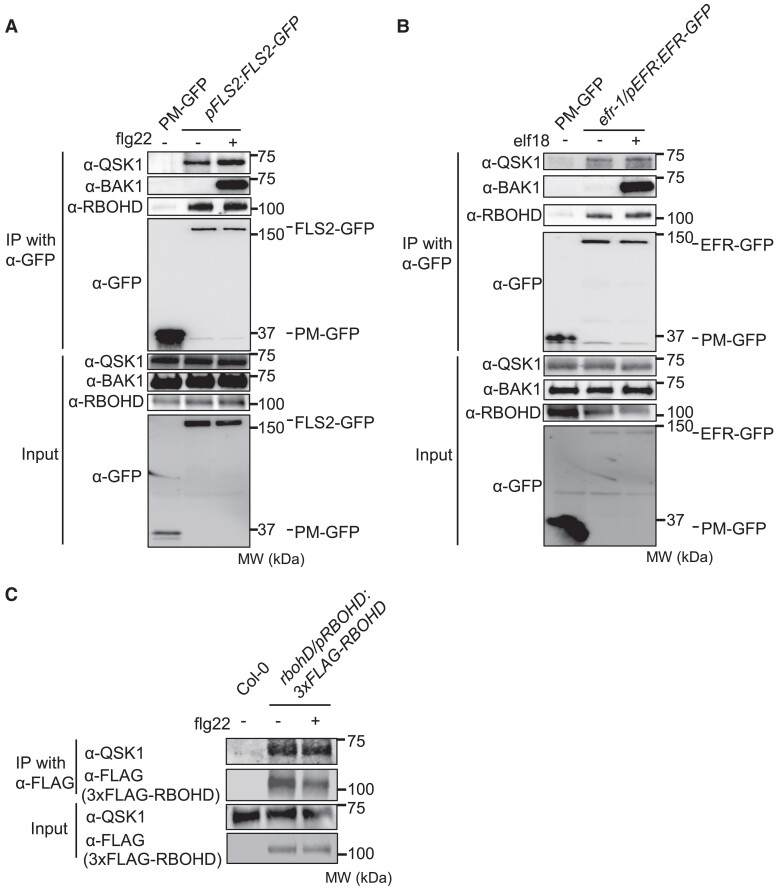
QSK1 associates with FLS2, EFR, and RBOHD in *A. thaliana*. **A**, **B)** Two-week-old *Arabidopsis* seedlings of *pFLS2:FLS2-GFP*, *efr-1/pEFR:EFR-GFP*, or PM-GFP (*p35S: LTI6b-GFP*) were treated with (+) or without (−) 1 *µ*m flg22 or 1 *µ*m elf18 for 10 min. Total proteins (input) were immunoprecipitated with α-GFP magnetic beads, followed by immunoblotting with α-GFP, α-QSK1, α-BAK1, and α-RBOHD antibodies. LTI6b, a known plasma membrane protein, was used as a control to illustrate that QSK1, RBOHD, and BAK1 do not associate with GFP at the plasma membrane. The position of the closest protein marker to the band is indicated, with its molecular weight (MW) shown in kilodaltons. **C)** Two-week-old *Arabidopsis* seedlings of rbohD/pRBOHD:3xFLAG-RBOHD or Col-0 were treated with or without 1 *µ*m flg22 for 10 min, and the total proteins were immunoprecipitated with α-FLAG magnetic beads followed by immunoblotting with α-FLAG and α-QSK1 antibodies. Col-0 plants were used as a control to illustrate that QSK1 does not associate with α-FLAG nonspecifically. All the experiments were repeated 3 times with similar results.

QSK1 (AT3G02880) is of particular importance as multiple tryptic peptides were identified in the IPs with FLS2, EFR, and RBOHD (Supplementary Data Set S2). Notably, transient expression of *QSK1-3xHA* in *Nicotiana benthamiana* led to significant reduction in flg22-induced ROS production (Goto et al. 2024) (Supplementary Fig. S1). QSK1 is an LRR-RK with 5 LRRs in its ectodomain (Isner et al. 2018; Wu et al. 2019). To independently validate the association of QSK1 with FLS2, EFR, and RBOHD in *Arabidopsis*, we generated α-QSK1 antibodies. IP of FLS2-GFP from the *pFLS2:FLS2-GFP* stable transgenic line revealed a clear ligand-independent association between FLS2-GFP and endogenous QSK1 (Fig. 2A), in contrast to the ligand-dependent FLS2-BAK1 interaction. Furthermore, we conducted IP experiments with EFR-GFP and 3xFLAG-RBOHD from *efr-1/pEFR:EFR-GFP* and *rbohD/pRBOHD:3xFLAG-RBOHD*, respectively (Fig. 2, B and C). The data reveal that RBOHD and EFR form ligand-independent association with QSK1, suggesting that QSK1 is an integral component of the PRR-RBOHD complex prior to elicitation, and this association remains stable even after PAMP treatment.

### QSK1 negatively regulates PTI

**Figure 3.**
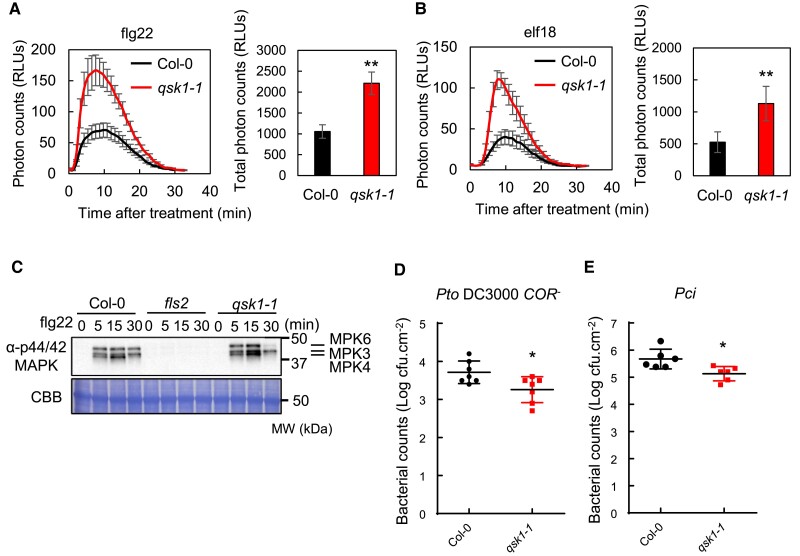
*Arabidopsis qsk1-1* mutant shows enhanced PTI responses compared to Col-0. **A**, **B)**  *qsk1-1* mutant has enhanced ROS production following treatment with flg22 and elf18. Eight leaf discs from 4- to 5-wk-old *Arabidopsis* plants were treated with 1 *µ*m flg22 **A)** or 1 *µ*m elf18 **B)**, and time course (left) and the total amount (right) of ROS production were measured by a luminol-based assay, with results shown in relative luminescence units (RLUs). Values are mean ± Se (*n* = 8). Double asterisks indicate significant differences (Student's *t*-test, ^**^P ≤ 0.01). **C)**  *qsk1-1* mutant induced enhanced MAPK activation following treatment with flg22. Ten-day-old *Arabidopsis* seedlings were treated with 1 *µ*m flg22, and phosphorylated MAPKs were detected on immunoblotting with α-phospho-p44/42 MAPK (Erk1/2) (Thr202/Tyr204) antibody. Equal loading of protein samples is shown by coomassie brilliant blue (CBB) staining. **D**, **E)**  *qsk1-1* mutant was more resistant to bacteria. *P. syringae* pv. *tomato* (*Pto*) DC3000 lacking the toxin coronatine (*COR^−^*) **D)** or *P. syringae* pv. *cilantro* (*Pci*) 0788-9 **E)** were sprayed onto leaf surfaces of 6-wk-old soil-grown *Arabidopsis* plants at a concentration of 1 × 10^5^ cfu/mL. Three-day postspray inoculation, leaves were harvested to determine bacterial growth. Values are means ± Sd from 7 plants for **D)** and 6 plants for **E)**. An asterisk indicates significant differences (Student's *t*-test, *P ≤ 0.05). All the experiments were repeated 3 times with similar results.

To elucidate the role of QSK1 in the regulation of PRR-RBOHD complexes, we conducted comprehensive characterization of the *Arabidopsis qsk1-1* mutant (SALK_ 019840) (Isner et al. 2018). The *qsk1-1* mutant harbors a T-DNA insertion within the first exon, resulting in pronounced reduction in *QSK1* transcript levels compared to Col-0 (Supplementary Fig. S2, A and B). In addition, immunoblotting with α-QSK1 antibodies failed to detect the QSK1 protein in the *qsk1-1* mutant (Supplementary Fig. S2C), indicating that *qsk1-1* is a null mutant. The *qsk1-1* mutant exhibited a significant increase in ROS production in response to flg22 and elf18 (Fig. 3, A and B). Furthermore, this mutant also showed enhanced MAPK activation 15 min following flg22 treatment (Fig. 3C). Collectively, these results indicate that QSK1 exerts a negative regulatory influence on PTI signaling pathways.

To gain further insights into the impact of QSK1 on disease resistance, we assessed the growth of the weakly virulent bacterial strain *Pto* DC3000 *COR^−^*, which lacks the toxin coronatine (COR) responsible for inducing stomatal reopening during infection (Melotto et al. 2006), and the nonadapted bacterium *Pseudomonas syringae* pv. *Cilantro* (*Pci*) 0788-9, known to exhibit poor growth on Col-0 plants (Lewis et al. 2008). Six-week-old *Arabidopsis* plants were spray inoculated with *Pto* DC3000 *COR^−^* and *Pci.* At 3 d postinoculation (dpi), *qsk1-1* demonstrated enhanced resistance compared to Col-0 (Fig. 3, D and E). We also tested the susceptibility of *qsk1-1* to *Pto* DC3000 *hrcC*^−^ upon spray inoculation and found that it showed enhanced resistance (Supplementary Fig. S3). These results highlight the role of QSK1 as negative regulator of plant resistance to bacterial disease.

To verify that the observed phenotype is due to the lack of *QSK1*, we generated the complementation line, *qsk1-1/pQSK1:QSK1-GFP*. This complementation reversed the enhanced ROS production upon treatment with flg22, elf18, and pep2 in the *qsk1-1* mutant (Supplementary Fig. S4, A to D). No morphological differences were observed among the *qsk1-1* mutant, *qsk1-1/pQSK1:QSK1-GFP* lines, and Col-0 (Supplementary Fig. S4E). These results confirm that the amplified PTI responses in the *qsk1-1* mutant are attributed to the absence of *QSK1*.

**Figure 4.**
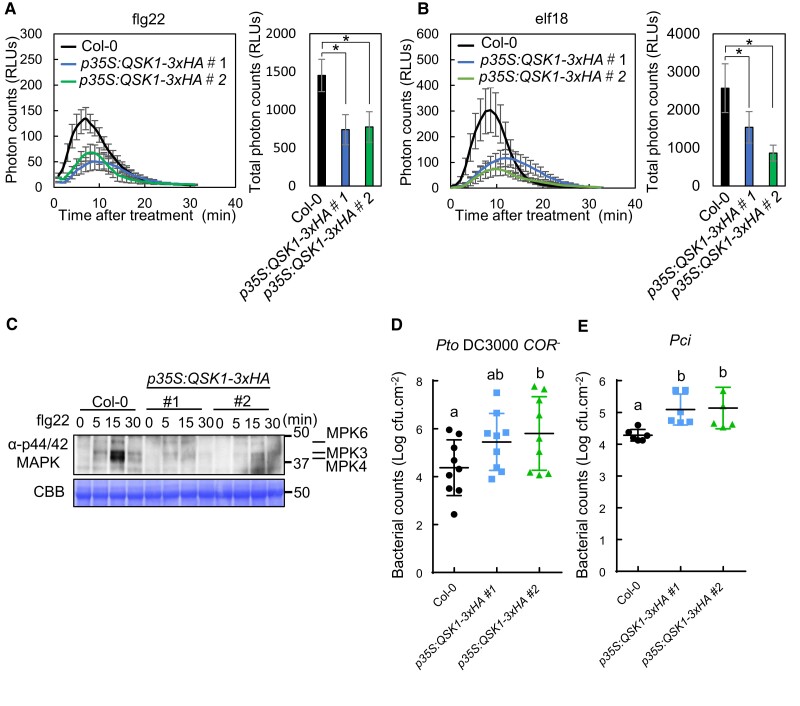
*Arabidopsis QSK1* overexpression lines (*p35S:QSK1-3xHA*) have reduced PTI responses compared to Col-0. **A**, **B)**  *p35S:QSK1-3xHA* lines showed reduced ROS production in response to flg22 and elf18. Eight 7-d-old *Arabidopsis* seedlings were treated with 1 *µ*m flg22 **A)** or 1 *µ*m elf18 **B)**, and time course (left) and the total amount (right) of ROS production were measured by a luminol-based assay. Values are mean ± Se (*n* = 8). An asterisk indicates significant differences (Student's *t*-test, *P ≤ 0.05). **C)**  *p35S:QSK1-3xHA* lines showed reduced MAPKs activation in response to flg22. Ten-day-old *Arabidopsis* seedlings were treated with 1 *µ*m flg22 and phosphorylated MAPKs were detected on immunoblotting with α-phospho-p44/42 MAPK (Erk1/2) (Thr202/Tyr204) antibody. Equal loading of protein samples is shown by CBB staining. **D**, **E)**  *p35S:QSK1-3xHA* lines were more susceptible to bacteria. *Pto* DC3000 C*OR*^−^  **D)** or *Pci*  **E)** were sprayed onto leaf surfaces of 6-wk-old soil-grown *Arabidopsis* plants at a concentration of 1 × 10^5^ cfu/mL. Three-day postspray inoculation, leaves were harvested to determine bacterial growth. Values are means ± Sd from 9 plants for **D)** and 6 plants for **E)**. Different letters indicate significantly different values at *P* ≤ 0.05 (1-way ANOVA, Tukey’s post hoc test). All the experiments were repeated 3 times with similar results.

To further investigate the role of QSK1 in modulating PRR-RBOHD complexes, we generated 2 independent *Arabidopsis* transgenic lines overexpressing *QSK1-3×HA* under the control of the *CaMV 35S* promoter (*p35S:QSK1-3×HA*). These lines exhibited markedly elevated *QSK1* transcript levels compared to Col-0 (Supplementary Fig. S5A) and produced a significantly higher amount of QSK1-3xHA protein than the endogenous QSK1 (Supplementary Fig. S5B). Morphological evaluations highlighted that the *p35S:QSK1-3×HA* lines had a marginally reduced size compared to both Col-0 and the *qsk1-1* mutant (Supplementary Fig. S5C). In stark contrast to the *qsk1-1* mutant, the *p35S:QSK1-3×HA* lines exhibited notably diminished ROS production upon treatment with flg22 and elf18 in comparison to Col-0 (Fig. 4, A and B). Additionally, *p35S:QSK1-3×HA* lines displayed attenuated MAPK activation in response to flg22 (Fig. 4C) and showed reduced resistance to *Pto* DC3000 *COR^−^* and *Pci* compared to Col-0 (Fig. 4, D and E). *p35S:QSK1-3×HA#2* line also showed reduced resistance to *Pto* DC3000 *hrcC*^−^ mutant (Supplementary Fig. S3). These results confirm that QSK1 plays an important role as a negative regulator in PTI in *Arabidopsis*. However, QSK1 is not involved in chitin-induced signaling as *qsk1-1*, *qsk1-1/pQSK1:QSK1-GFP*, and *p35S:QSK1-3×HA* lines induce similar chitin-induced ROS production compared to Col-0 (Supplementary Fig. S6). To determine the subcellular localization of QSK1 in plant cells, we transiently expressed a QSK1-GFP fusion protein in *N. benthamiana*. QSK1-GFP localizes at the plasma membrane (Supplementary Fig. S7, A and B). This subcellular localization was confirmed in *Arabidopsis* using a stable transgenic line, *qsk1-1*/*pQSK1:QSK1-GFP* (Supplementary Fig. S7C). Additionally, we examined the transcriptional response of *QSK1* to PAMP treatments. Treatment with flg22 and elf18 led to an increase in *QSK1* transcript levels (Supplementary Fig. S8), indicating its transcriptional upregulation upon PAMP recognition.

### QSK1 negatively regulates PRR protein levels

**Figure 5.**
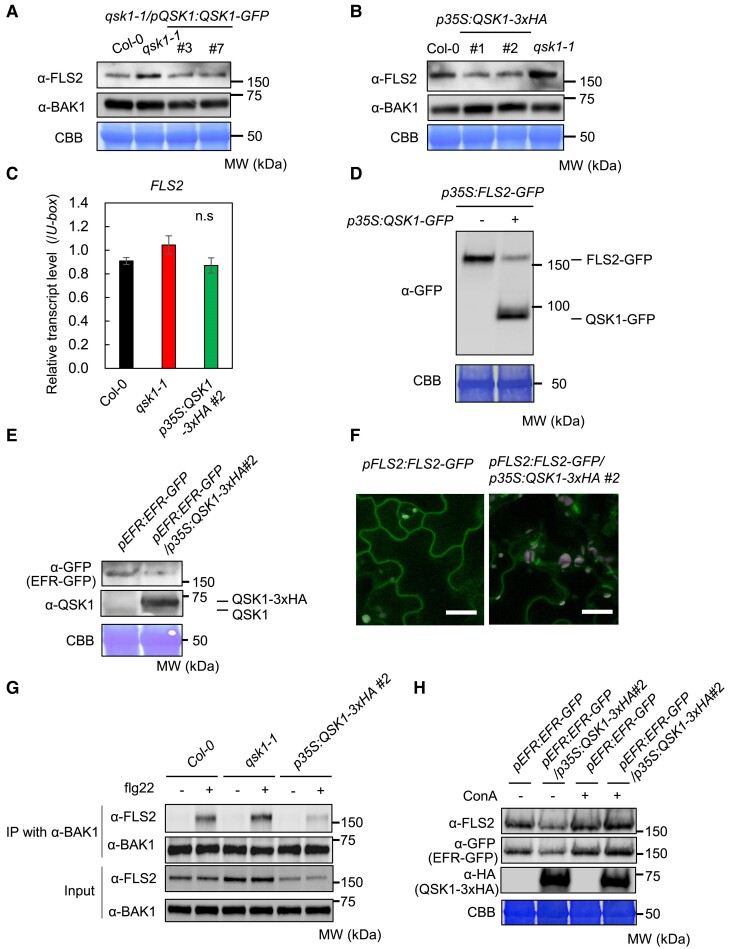
QSK1 negatively regulates FLS2 and EFR accumulation. **A)** FLS2 protein accumulates more in *qsk1-1* mutant than in Col-0 and the complementation lines (*qsk1-1/pQSK1:QSK1-GFP*). **B)** FLS2 protein accumulates less in *p35S:QSK1-3xHA* lines than in Col-0. FLS2 and BAK1 protein levels of 2-wk-old *Arabidopsis* seedlings were measured by immunoblotting with α-FLS2 and α-BAK1 antibodies. Equal loading of protein samples is shown by coomassie brilliant blue (CBB) staining. **C)** FLS2 transcript levels are not changed in Col-0, *qsk1-1* mutant, and *p35S:QSK1-3xHA* lines. Transcript levels of *FLS2* in 2-wk-old *Arabidopsis* seedlings were measured by RT-qPCR after normalization to the *U*-*box* housekeeping gene transcript (*At5g15400*). Values are presented as mean ± Se derived from 3 independent experiments, with each experiment utilizing 3 different plants. There are no significant differences at *P* ≤ 0.05 (1-way ANOVA, Tukey's post hoc test). **D)** The expression of *QSK1-GFP* reduces FLS2-GFP protein levels in *N. benthamiana.* FLS2-GFP was transiently expressed with (+) or without (−) QSK1-GFP under the control of *p35S* promoter, and their protein levels were measured 3 d after agroinfiltration by immunoblotting with α-GFP antibodies. *Agrobacterium* concentration (OD600 = 0.6) was adjusted with empty *Agrobacterium*. Equal loading of protein samples is shown by CBB staining. **E)** QSK1 reduces EFR protein levels. Protein levels of EFR-GFP and QSK1 in 2-wk-old *Arabidopsis* seedlings of *pEFR:EFR-GFP* and *pEFR:EFR-GFP*/*p35S:QSK1-3xHA#2* were measured by immunoblotting with α-GFP antibodies. Equal loading of protein samples is shown by CBB staining. **F)** QSK1 reduces FLS2 protein accumulation at the plasma membrane. The localization of FLS2-GFP in cotyledons of 10-d-old seedlings of *pFLS2:FLS2-GFP* line and *pFLS2:FLS2-GFP/p35S:QSK1-3xHA#2* line was observed by confocal microscopy. The white bars represent 30 *μ*m. **G)** QSK1 reduces flg22-inducible FLS2-BAK1 interaction. Two-week-old *Arabidopsis* seedlings of Col-0, *qsk1-1*, or *p35S:QSK1-3xHA #2* line were treated with (+) or without (−) 1 *µ*m flg22 for 10 min. Total proteins (input) were immunoprecipitated with α-BAK1 antibody, followed by immunoblotting with α-BAK1 and α-FLS2 antibodies. **H)** ConA suppresses QSK1-mediated PRR reduction. Two-week-old *Arabidopsis* seedlings of *pEFR:EFR-GFP* and *p35S:QSK1-3xHA#2*/*pEFR:EFR-GFP* lines were treated with (+) or without (−) 1 *μ*m ConA for 10 h. The protein levels of FLS2, EFR-GFP, and QSK1-3xHA were measured by immunoblotting. Equal loading of protein samples is shown by CBB staining. All the experiments were repeated 3 times with similar results.

Since QSK1 negatively regulates both ROS production and MAPK activation, 2 distinct signaling events following PAMP recognition (Xu et al. 2014), we hypothesized that QSK1 might influence the activity or stability of PRRs. Immunoblotting showed elevated FLS2 protein abundance in the *qsk1-1* mutant relative to Col-0 and the complemented *qsk1-1*/*pQSK1:QSK1-GFP* lines, while BAK1 and RBOHD levels remained unaffected (Fig. 5A; Supplementary Figs. S9A and S10). Conversely, FLS2 protein levels were reduced in QSK1 overexpression lines (*p35S:QSK1-3×HA*) compared to Col-0 (Fig. 5B; Supplementary Fig. S9B). This regulatory mechanism does not appear to operate at the transcriptional level since *FLS2* mRNA amounts were comparable among Col-0, *qsk1-1*, and *p35S:QSK1-3×HA lines* (Fig. 5C). Supporting this notion, *N. benthamiana* plants coexpressing *FLS2-GFP* and *QSK1-GFP* under the control of the *CaMV 35S* promoters exhibited reduced FLS2-GFP protein levels (Fig. 5D). Similarly, overexpression of *QSK1* led to a decline in EFR protein levels; the EFR-GFP levels in *pEFR:EFR-GFP/p35S:QSK1-3×HA* line were lower than those in *pEFR:EFR-GFP* lines (Fig. 5E; Supplementary Fig. S9C). Further investigation into the impact of QSK1 on the subcellular distribution of FLS2-GFP revealed a notable reduction in plasma membrane localization when coexpressed with *QSK1* in the *pFLS2:FLS2-GFP*/*p35S:QSK1-3xHA* line (Fig. 5F). These results suggest that QSK1 exerts a negative regulatory effect on FLS2 and EFR protein accumulation at the plasma membrane. Consequently, BAK1 interacts more with FLS2 in *qsk1-1* and less so in *p35S:QSK1-3xHA#2* upon treatment with flg22 (Fig. 5G).

FLS2 protein levels decrease 1 h after flg22 treatment due to protein degradation following endocytosis (Robatzek et al. 2006). In *qsk1-1*, basal FLS2 protein levels were increased, but flg22 treatment reduced FLS2 protein abundance (Supplementary Fig. S11A). In contrast, in *p35S:QSK1-3xHA#2*, basal FLS2 protein levels were lower, and flg22 treatment did not further reduce FLS2 protein abundance (Supplementary Fig. S11B). Similarly, basal EFR-GFP protein levels were decreased in *pEFR:EFR-GFP/p35S:GSK1-3xHA* #2, and elf18 treatment did not further reduce EFR-GFP protein abundance (Supplementary Fig. S11C). This suggests that in *p35S:QSK1-3xHA#2*, a portion of the FLS2 and EFR proteins may be nonfunctional or mislocalized, preventing their recognition of PAMPs, subsequent endocytosis, and degradation following PAMP recognition.

To elucidate the mechanism behind QSK1's modulation of FLS2 protein levels, we assessed the importance of its catalytic residue. D488N mutation in QSK1 is thought to prevent the nucleophilic attack on the gamma-phosphate of the ATP molecule, thus reducing the enzyme's activity to 0 (Aryal et al. 2023). We heterologously expressed QSK1-mCherry and QSK1(D488N)-mCherry in *N. benthamiana* and checked flg22-induced ROS production and FLS2 protein abundance after coexpression (Supplementary Fig. S12). Unexpectedly, QSK1(D488N)-mCherry was expressed at much higher levels than QSK1-mCherry, reduced FLS2 protein abundance, and inhibited flg22-induced ROS production more effectively. These results indicate that the kinase activity of QSK1 is not required for the regulation of FLS2, and the QSK1 abundance is the key factor for FLS2 reduction and inhibition of PTI.

Next, we employed a pharmacological approach, using an array of inhibitors: MG132 (proteasome inhibitor), bafilomycin A1 (vacuolar-type-H^+^-ATPase inhibitor), E-64d (cysteine protease inhibitor), TLCK (serine protease inhibitor), wortmannin (phosphatidylinositol 3-kinase inhibitor), brefeldin A (Endoplasmic reticulum-Golgi transport inhibitor), cycloheximide (protein synthesis inhibitor), and concanamycin A (ConA, vacuolar-type-H^+^-ATPase inhibitor) (Fig. 5H; Supplementary Fig. S13). Notably, ConA mitigated the QSK1-mediated reduction of both FLS2 and EFR levels (Fig. 5H). ConA is known to block vacuolar transport, thereby impeding autophagic degradation pathway as well as the endocytosis-mediated degradation pathway (Dettmer et al. 2006; Scheuring et al. 2011). These findings suggest that *QSK1* overexpression may facilitate vacuolar degradation of PRRs through the autophagy pathway or the endocytosis pathway. In contrast, without QSK1 overexpression, ConA only slightly increased FLS2 and EFR levels in *pEFR:EFR-GFP* line (Lane 1 vs Lane 3 in Fig. 5H). This is likely because QSK1-mediated negative regulation of FLS2 and EFR through vacuolar degradation is weaker in *pEFR:EFR-GFP* than in *pEFR:EFR-GFP*/*p35S:QSK1-3xHA* line due to the lower QSK1 levels.

### HopF2*_Pto_*-HA interacts with QSK1 and reduces FLS2 protein levels

**Figure 6.**
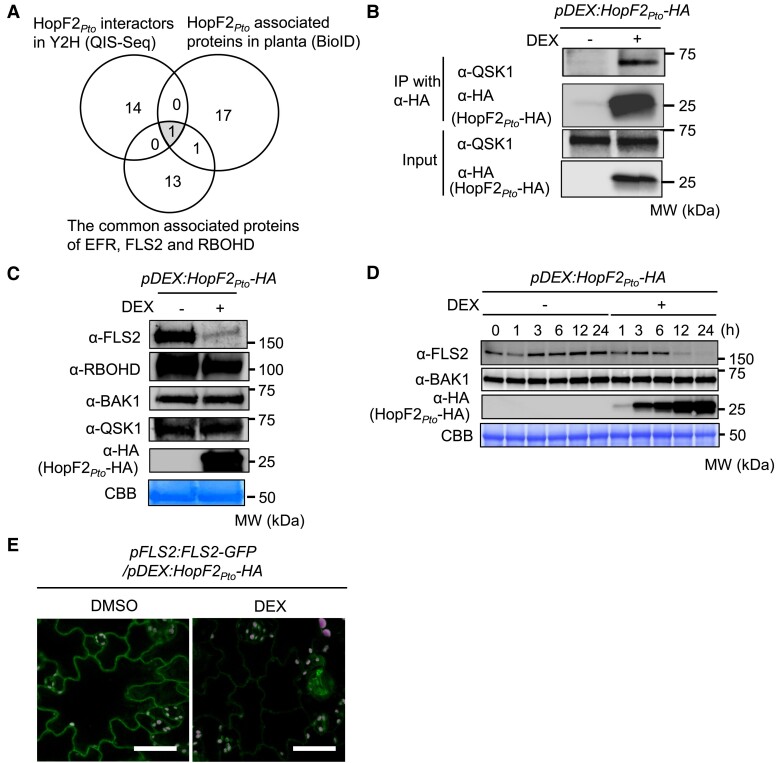
HopF2*_Pto_* associates with QSK1 and reduces FLS2 protein level. **A)** Comparison of candidate interactors of HopF2*_Pto_* and the commonly associated proteins with EFR, FLS2, and RBOHD. The Venn diagram illustrates candidate HopF2*_Pto_* interactors identified by yeast 2-hybrid screening coupled with next-generation sequencing (QIS-seq) and by proximity-dependent BioID in planta (Khan et al. 2018) with the commonly associated proteins with EFR, FLS2, and RBOHD identified in this study. **B)** HopF2*_Pto_* associates with QSK1 in vivo. Two-week-old *Arabidopsis* seedlings of *pDEX:HopF2_Pto_-HA* were treated with (+) or without (−) 30 *µ*m DEX for 24 h. Total proteins (input) were immunoprecipitated with α-HA magnetic beads followed by immunoblotting with α-HA and α-QSK antibodies. **C**, **D)** HopF2*_Pto_* specifically reduced FLS2 protein accumulation. Two-week-old *Arabidopsis* seedlings of *pDEX:HopF2_Pto_-HA* were treated with (+) or without (−) 30 *µ*m DEX and FLS2, RBOHD, BAK1, QSK1, and HopF2*_Pto_*-HA protein levels were measured by immunoblotting. Equal loading of protein samples is shown by coomassie brilliant blue (CBB) staining. **E)** HopF2*_Pto_* reduced FLS2 protein accumulation at the plasma membrane. Ten-day-old seedlings of *pFLS2:FLS2-GFP/pDEX:HopF2_Pto_-HA* line were treated with 30 *µ*m DEX or DMSO for 24 h, and the localization of FLS2-GFP in cotyledons was observed by confocal microscopy. The white bar represents 50 *μ*m. All the experiments were repeated 3 times with similar results.

QSK1 could represent a potential effector target as part of PRR complexes because plant pathogens often deploy virulence effectors to target the PRR complex to effectively suppress PTI. Our attention was drawn to HopF2*_Pto_* from *Pto* DC3000, known to be a potent inhibition of early PTI responses (Wilton et al. 2010; Wu et al. 2011; Hurley et al. 2014; Zhou et al. 2014), as a likely candidate effector targeting QSK1, for several reasons. Firstly, Khan et al. (2018) conducted enzyme-catalyzed proximity labeling of HopF2*_Pto_* (proximity-dependent biotin identification [BioID]) and identified QSK1 as one of the 19 biotinylated proteins. Secondly, we employed a combination of yeast 2-hybrid methods with next-generation sequencing, known as QIS-seq (Lewis et al. 2012), and revealed QSK1 as one of the 15 potential targets (Fig. 6A; Supplementary Data Set S3_1). Thirdly, a comparative analysis of potential HopF2*_Pto_* interactors by QIS-seq (quantitative interactor screening with next-generation sequencing) and BioID, alongside PRR complex components, using a Venn diagram (Fig. 6A), highlighted QSK1 as the sole common factor across all 3 data sets (Fig. 6A; Supplementary Data Set S3_2). This finding aligns with previous IP-MS experiments by Hurley et al. (2014), which also listed QSK1 among the proteins interacting with HopF2*_Pto_* when expressed in *Arabidopsis*.

To validate the interaction between HopF2*_Pto_*-HA and endogenous QSK1 in vivo, we employed the dexamethasone (DEX)-inducible system in transgenic *Arabidopsis* carrying the *pDEX:HopF2_Pto_-HA* construct. Our results show in vivo interaction between HopF2*_Pto_*-HA and QSK1 upon DEX treatment (Fig. 6B). To assess the impact of HopF2*_Pto_* on PRR complexes, we examined the protein levels of FLS2, RBOHD, BAK1, and QSK1 with or without expression of *HopF2_Pto_-HA* (Fig. 6C). Strikingly, HopF2*_Pto_*-HA specifically diminished the protein levels of FLS2 without affecting the other proteins. The reduction in FLS2 coincided with an increase in the levels of HopF2*_Pto_*-HA following DEX treatment (Fig. 6D; Supplementary Fig. S14). Next, we examined the effects of HopF2*_Pto_*-HA on the subcellular localization of FLS2-GFP (Fig. 6E). In the absence of HopF2*_Pto_*-HA expression, FLS2-GFP predominantly localized to the plasma membrane. However, induction of *HopF2_Pto_-HA* expression by DEX treatment led to a significant reduction of FLS2-GFP at the plasma membrane.

### The catalytic residue D175 of HopF2*_Pto_* is required for its virulence function

**Figure 7.**
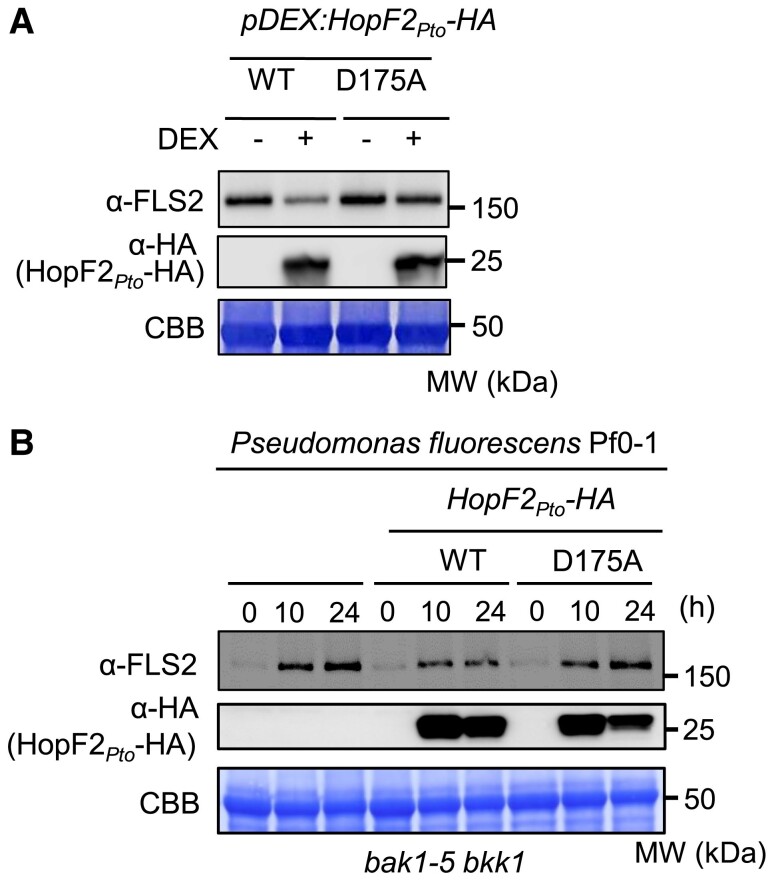
Mono-ADP ribosylation (MARylation) activity of HopF2*_Pto_* is required for the FLS2 elimination. **A)** The catalytic residue D175 for MARylation activity in HopF2_Pto_ is required for the inhibition of FLS2 accumulation. Two-week-old *Arabidopsis* seedlings of *pDEX: HopF2_Pto_-HA* and *pDEX:HopF2_Pto_* (D175A)*-HA* were treated without (−) or with (+) 5 nm and 50 *µ*m DEX for 24 h, respectively. FLS2 and HopF2*_Pto_*-HA protein levels were measured by immunoblotting. **B)** HopF2*_Pto_* inhibits FLS2 protein accumulation during infection. Immunoblotting detecting FLS2 and HopF2*_Pto_*-HA in Col-0 during bacterial infection after syringe inoculation with *P. fluorescens* Pf0-1, *P. fluorescens* Pf0-1 *HopF2_Pto_-HA*, or *P. fluorescens* Pf0-1 *HopF2_Pto_* (D175A)*-HA*. Equal loading of protein samples is shown by coomassie brilliant blue (CBB) staining. All the experiments were repeated 3 times with similar results.

A mutation in the catalytic residue D175 (D175A) of HopF2*_Pto_* leads to a significant reduction in its virulence, indicating the indispensable role of mono-ADP ribosylation (MARylation) in the functionality of HopF2*_Pto_* (Wilton et al. 2010). We compared the effect of this mutation using a transgenic *pDEX:HopF2*_Pto_*-HA* D175A line. However, the D175A protein may be unstable, or *pDEX:HopF2*_Pto_*-HA* D175A line may not produce HopF2*_Pto_*-HA (D175A) as efficiently as *pDEX:HopF2*_Pto_*-HA* wild-type line upon treatment with the same DEX concentration. To address this, we treated both lines with different DEX concentrations to find the optimal DEX concentration that induces HopF2*_Pto_*-HA protein accumulation to similar levels (Supplementary Fig. S15). Using this condition, we compared FLS2 protein abundance in *pDEX:HopF2*_Pto_*-HA* and *pDEX:HopF2*_Pto_ (D175A)*-HA* after treatment with DEX. Notably, DEX-induced expression of *pDEX:HopF2*_Pto_*-HA*, but not *HopF2_Pto_* (D175A)*-HA*, decreases FLS2 protein levels (Fig. 7A), suggesting that MARylation activity is essential for HopF2*_Pto_*'s ability to deplete FLS2. To further investigate the effects of HopF2*_Pto_* and its MARylation activity on FLS2 during infection, we introduced both the wild-type HopF2*_Pto_*-HA and its D175A mutant into the nonpathogenic bacteria *Pseudomonas fluorescens* Pf0-1 (Fig. 7B). We selected *P. fluorescens* Pf0-1 due to its absence of virulence effectors, allowing a focused examination of HopF2*_Pto_* effects. In natural infections, multiple PAMPs from bacteria may rapidly trigger PTI responses, leading to the transcriptional upregulation of *FLS2* and subsequent FLS2 accumulation. To minimize PTI-induced FLS2 accumulation during infection, we used *bak1-5 bkk1* double mutants for the infection assay, as flg22-, elf18-, and pep1-mediated PTI responses are dramatically reduced in *bak1-5 bkk1* mutant (Roux et al. 2011). Although the suppression of FLS2 accumulation in *bak1-5 bkk1* mutant after bacterial inoculation was not complete, the infection with *P. fluorescens* Pf0-1 harboring *HopF2_Pto_-HA* for 10 h resulted in increased levels of HopF2*_Pto_*-HA and concurrent suppression of FLS2 accumulation, compared to both untransformed *P. fluorescens* Pf0-1 and *P. fluorescen*s Pf0-1 harboring *HopF2_Pto_*(D175A)*-HA*. These data demonstrate that HopF2*_Pto_*-HA actively reduces FLS2 protein levels during infection and that the MARylation activity of HopF2*_Pto_* is required for this function. Next, we performed a pharmacological assay that involved a range of inhibitors, including ConA, E-64d, 3-methyladenine (PI3K inhibitor), bafilomycin A1, wortmannin, brefeldin A, MG132, and TLCK, but none of these inhibitors succeeded in counteracting the FLS2 depletion induced by HopF2*_Pto_* (Supplementary Fig. S16).

### HopF2*_Pto_* modulates the expression of immune-related genes in *Arabidopsis*

To explore the influence of HopF2*_Pto_* on plant immune responses, RNA-seq analysis was performed on *Arabidopsis* Col-0 and *pDEX:HopF2_Pto_-HA* seedlings, 24 h posttreatment with DMSO or DEX. The multidimensional scaling plot displayed consistent global gene expression patterns across all 4 biological replicates for both treatments (Supplementary Fig. S17A). Notably, the *pDEX:HopF2_Pto_-HA* line exhibited significant transcriptional changes upon DEX treatment, whereas DEX treatment in Col-0 led to only minor alterations in gene expression compared to those in the Col-0 and *pDEX:HopF2_Pto_-HA* lines treated with DMSO.

To differentiate gene expression changes induced by HopF2*_Pto_* from those solely caused by DEX, we compared the gene expression in the DEX-treated *pDEX:HopF2_Pto_-HA* line with DEX-treated Col-0. In the DEX-treated *pDEX:HopF2_Pto_-HA* line, we observed an upregulation of 1,399 genes and a downregulation of 2,708 genes by at least 2-fold, along with 330 genes upregulated and 879 genes downregulated by at least 4-fold (Supplementary Data Set S4). Gene ontology (GO) enrichment analyses conducted on highly upregulated (330 genes, log2 fold change ≥ 2, false discovery rate [FDR] ≤ 0.05) and highly downregulated (879 genes, log2 fold change ≤ −2, FDR ≤ 0.05) genes provided insights into the biological significance of these transcriptional changes (Supplementary Data Sets S5_1 and S5_2). Remarkably, both upregulated and downregulated genes were significantly associated with GO terms related to biotic stress responses and immunity, underlining HopF2*_Pto_*'s crucial role in modulating specific immune-related genes in *Arabidopsis*.

To pinpoint genes distinctively affected by *HopF2_Pto_* expression, self-organizing map (SOM) clustering was applied to the most differentially expressed genes, focusing on the top 25% based on their coefficient of variation across samples. These genes were grouped into 12 clusters, reflecting unique expression patterns in Col-0 and *pDEX:HopF2_Pto_-HA* following either DMSO or DEX treatment (Supplementary Fig. S17B and Data Set S6). Notably, genes in Cluster 1 were exclusively upregulated by HopF2*_Pto_*, whereas those in Cluster 2 were specifically downregulated. The GO enrichment analysis revealed that both clusters were enriched in GO terms associated with biotic stress responses and immunity (Supplementary Data Sets S5_3 and S5_4), and Cluster 2 exhibited a pronounced enrichment for GO terms like “membrane,” “cell periphery,” and “plasma membrane.” These observations suggest that HopF2*_Pto_* selectively modulates gene expression related to immune response and plasma membrane-associated proteins.

**Figure 8.**
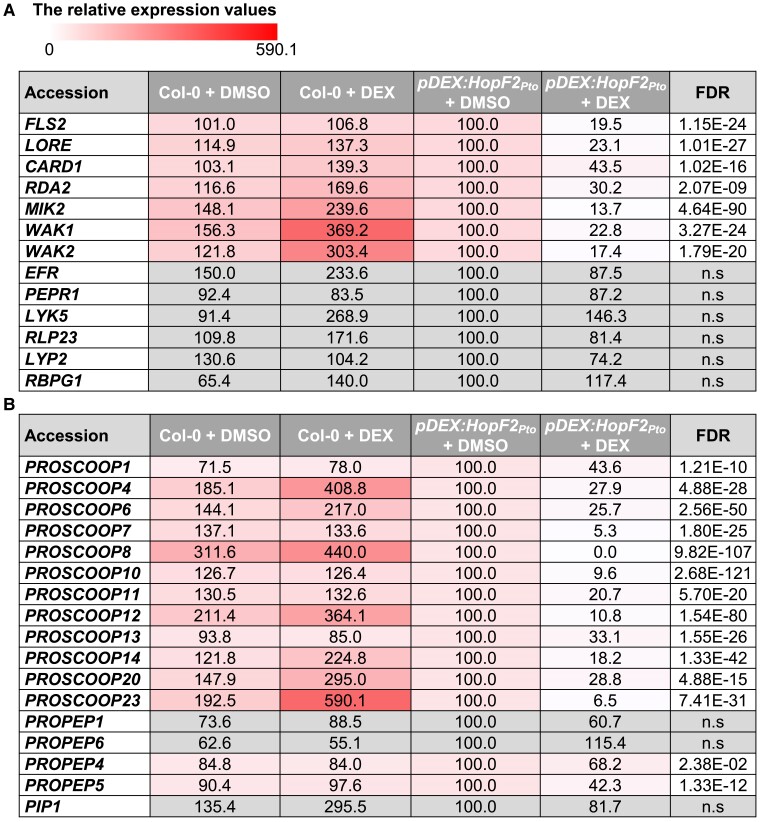
HopF2*_Pto_* reduces transcript levels of *PRRs* and *PROSCOOPs*. Transcript levels of *PRRs*  **A)**, *PROSCOOPs*, *PROPEPs*, and *PIP1*  **B)** were measured by RNA-seq in 2-wk-old seedlings of Col-0 and *pDEX:HopF2_Pto_-HA* after treatment with 30 *µ*m DEX for 24 h. The relative expression values of the genes are shown compared to the “*pDEX:HopF2_Pto_* + DMSO” control. The FDR values between “*pDEX:HopF2_Pto_* + DMSO” and “*pDEX:HopF2_Pto_* + DEX” are shown. Heatmaps indicate relative gene expression values. Gray boxes in the heat map indicate no statistically significant difference at FDR ≤ 0.05.

Given HopF2*_Pto_*'s role in diminishing FLS2 levels, we assessed the transcript levels of known *PRRs* (Fig. 8A). Notably, our data showed that HopF2*_Pto_* significantly reduces the transcript levels of certain *PRRs*, such as *FLS2*, *LIPOOLIGOSACCHARIDE-SPECIFIC REDUCED ELICITATION* (*LORE*, a PRR for bacterial fatty acid metabolite 3-OH-C10:0) (Kutschera et al. 2019), and *MALE DISCOVERER 1-INTERACTING RECEPTOR-LIKE KINASE 2* (*MIK2*, a PRR for SCOOP phytocytokines) (Hou et al. 2021; Rhodes et al. 2021; Yang et al. 2023), as well as *IOS1*, an important regulator of PRR complexes (Yeh et al. 2016) (Supplementary Fig. S18). Such reduction in transcript levels likely contributes to HopF2*_Pto_*'s suppression of PTI responses, as corroborated by our observation that HopF2*_Pto_* inhibits ROS production mediated by FLS2 and MIK2 (Supplementary Fig. S19).

### HopF2*_Pto_* reduces transcript levels of most *PROSCOOP* genes

Beyond inhibiting *PRR* gene expression, HopF2*_Pto_* also downregulates SCOOP phytocytokine signaling. SCOOP phytocytokines, exclusive to the Brassicaceae family, are a unique group of peptides that are cleaved from the C-terminus of their respective precursors, termed PROSCOOPs (Gully et al. 2019; Yang et al. 2023). Our transcriptomic analysis revealed that HopF2*_Pto_* significantly downregulates the transcript levels of multiple *PROSCOOPs*, especially *PROSCOOP7*, *8*, *10*, *12*, and *23* (Fig. 8B), while its effect on *PROPEPs* and *PROPIP1*, encoding other stress-regulated peptides, is minimal. This suggests HopF2*_Pto_*'s role in attenuating SCOOP phytocytokine signaling by downregulating both *PROSCOOPs* and *MIK2* gene expression.

### HopF2*_Pto_* reduces EFR protein levels possibly through vacuolar degradation

**Figure 9.**
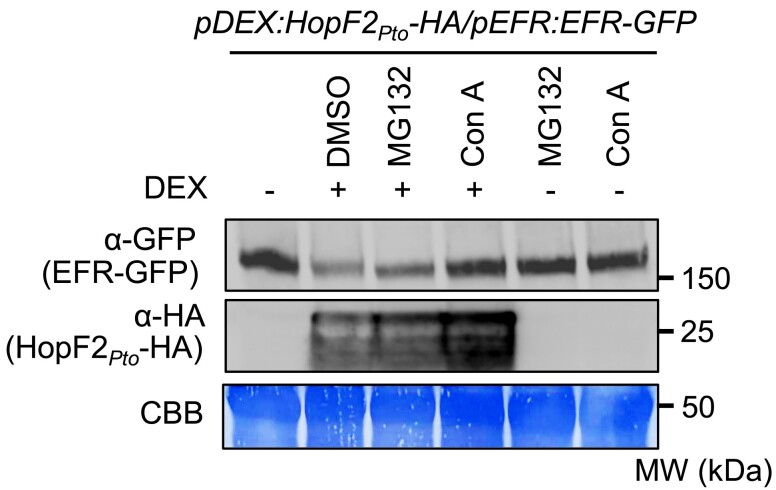
ConA inhibits HopF2*_Pto_* -mediated reduction of EFR expression. Two-week-old *Arabidopsis* seedlings of *pDEX:HopF2_Pto_-HA*/*pEFR:EFR-GFP* were treated with (+) or without (−) 30 *µ*m DEX for 24 h, followed by the treatment with DMSO, 100 *μ*m MG132, or 1 *μ*m ConA for 10 h. The protein levels of EFR-GFP and HopF2*_Pto_*-HA were measured by immunoblotting with α-GFP and α-HA antibodies. Equal loading of protein samples is shown by coomassie brilliant blue (CBB) staining. This experiment was repeated 3 times with similar results.

While HopF2*_Pto_* reduces the expression of *FLS2*, *LORE*, and *MIK2*, it does not affect the expression of other PRRs such as *EFR* and *PEPR1* (*PEP RECEPTOR1*, a PRR for Pep1 and Pep2 peptides) (Fig. 8A). Nevertheless, HopF2*_Pto_* effectively impairs ROS production and MAPK activation triggered by these PRRs (Supplementary Figs. S19 and S20), indicating that HopF2*_Pto_* may also employ a transcription-independent mechanism to inhibit PTI. This insight prompted further exploration into how HopF2*_Pto_* affects the EFR signaling pathway. We generated a homozygous *pDEX:HopF2_Pto_-HA/pEFR:EFR-GFP* line to assess the impact of *HopF2_Pto_-HA* expression on EFR-GFP levels. Remarkably, DEX-induced *HopF2_Pto_-HA* expression led to a decrease in EFR protein levels, suggesting that HopF2*_Pto_* exerts its influence on EFR protein levels via transcription-independent mechanisms (Fig. 9). Interestingly, ConA effectively countered the HopF2*_Pto_*-mediated reduction in EFR protein levels, implying that this reduction might occur via vacuolar degradation through either the autophagy pathway or the endocytosis pathway. Additionally, we assessed the effect of the proteasome inhibitor MG132, which only slightly inhibited the reduction in EFR protein levels. In contrast, both ConA and MG132 did not counter the HopF2*_Pto_*-mediated reduction in FLS2 protein levels (Supplementary Fig. S16), possibly because HopF2*_Pto_* reduces transcription levels of *FLS2* (Fig. 8A). This reduction may lead to insufficient levels for de novo synthesis of FLS2 protein, even if ConA and MG132 block QSK1-mediated FLS2 degradation.

### HopF2*_Pto_* requires QSK1 for its stabilization

**Figure 10.**
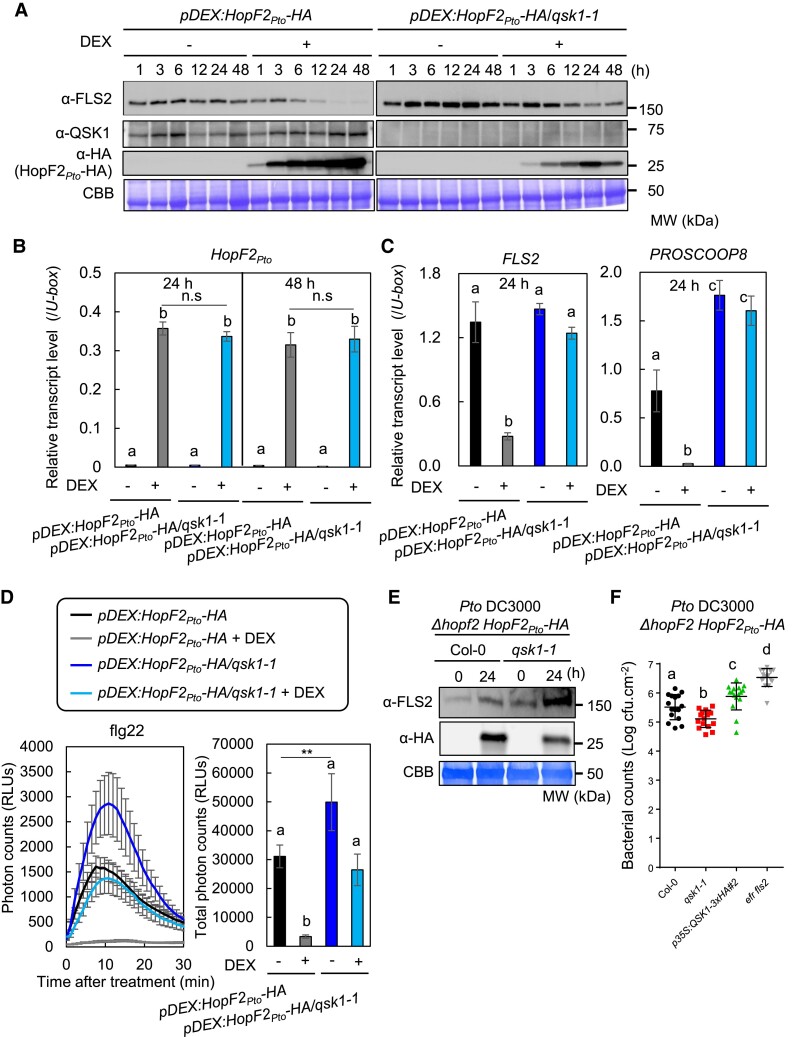
HopF2*_Pto_* requires QSK1 for its protein accumulation and function. **A)** HopF2*_Pto_* requires QSK1 for its protein accumulation and suppression of FLS2 accumulation. Two-week-old *Arabidopsis* seedlings of *pDEX:HopF2_Pto_-HA* and *pDEX:HopF2_Pto_-HA*/*qsk1-1* were treated with (+) or without (−) 30 *µ*m DEX for 1, 3, 6, 12, 24, and 48 h and FLS2, QSK1, and HopF2*_Pto_*-HA protein levels were measured by immunoblotting with α-FLS2, α-QSK1, and α-HA antibodies. Equal loading of protein samples is shown by CBB staining. **B)** QSK1 does not affect *HopF2_Pto_* transcript levels. Transcript levels of *HopF2_Pto_-HA* in 2-wk-old *Arabidopsis* seedlings of *pDEX:HopF2_Pto_-HA* and *pDEX:HopF2_Pto_-HA*/*qsk1-1* treated with (+) or without (−) 30 *µ*m DEX for 24 and 48 h were measured by RT-qPCR after normalization to the *U*-*box* housekeeping gene transcript (*At5g15400*). Values are mean ± Se of 3 biological replicates. There are no significant differences at *P* ≤ 0.05 between the 2 lines with DEX treatment (Student's *t*-test). Different letters indicate significantly different values at *P* ≤ 0.0001 (1-way ANOVA, Tukey’s post hoc test). **C)** HopF2*_Pto_* requires QSK1 for suppression of *FLS2* and *PROSCOOP8* transcript levels. The experimental condition is the same to **B)**. Values are mean ± Se from 3 plants. Different letters indicate significantly different values at *P* ≤ 0.05 (1-way ANOVA, Tukey’s post hoc test). **D)** HopF2*_Pto_* requires QSK1 to inhibit flg22-inducible ROS production. Seven-day-old *Arabidopsis* seedlings of *pDEX:HopF2_Pto_-HA* and *pDEX:HopF2_Pto_-HA*/*qsk1-1* were treated with (+) or without (−) 30 *µ*m DEX for 24 h, followed by the treated with 1 *µ*m flg22. The time course (left) and the total amount (right) of flg22-inducible ROS production were measured by a luminol-based assay. Values are mean ± Se from 16 leaf discs. Different letters indicate significantly different values at *P* ≤ 0.05 (1-way ANOVA, Tukey’s post hoc test). An asterisk indicates significant differences (Student's *t*-test, **P ≤ 0.01). **E)** HopF2*_Pto_* requires QSK1 during infection. Five-week-old *Arabidopsis* Col-0 and *qsk1-1* mutant were syringe inoculated with *Pto* DC3000 *Δhopf2 HopF2_Pto_-HA* (inoculum: 10^8^ cfu/mL). Immunoblotting detecting FLS2 and HopF2*_Pto_*-HA at 1 dpi. The similar bacterial population at 1 dpi was confirmed by the bacterial growth assay shown in Supplementary Fig. S22. Equal loading of protein samples is shown by CBB staining. **F)**  *qsk1-1* mutant is more resistant against *Pto* DC3000 *Δhopf2 HopF2_Pto_-HA*. *Pto* DC3000 *Δhopf2 HopF2_Pto_-HA* was sprayed onto leaf surfaces of 5-wk-old soil-grown *Arabidopsis* plants at a concentration of 1 × 10^5^ cfu/mL. Values are means ± Sd from 16 plants. The central horizontal line indicates the mean value. Different letters indicate significantly different values at *P* ≤ 0.05 (1-way ANOVA, Tukey’s post hoc test). The experiments were repeated 3 times with similar results.

To investigate the functional relationship between HopF2*_Pto_* and QSK1, we generated a *pDEX:HopF2_Pto_-HA/qsk1-1* homozygous line by crossing and checked the HopF2*_Pto_*-mediated reduction of FLS2 protein in a *qsk1* knockout background (Fig. 10A). Remarkably, the absence of QSK1 significantly reduces HopF2*_Pto_*'s ability to reduce FLS2 levels, showing the crucial role of QSK1 in HopF2*_Pto_* function. Intriguingly, HopF2*_Pto_*-HA protein levels were decreased in the *qsk1-1* mutant, suggesting a potential dependence of HopF2*_Pto_*-HA on QSK1 for both its accumulation and functionality in plants. Furthermore, reverse transcription quantitative PCR (RT-qPCR) analysis showed comparable DEX-induced expression of *HopF2_Pto_-HA* in both *pDEX:HopF2_Pto_-HA* and *pDEX:HopF2_Pto_-HA/qsk1-1* lines (Fig. 10B), suggesting that the dependency of HopF2*_Pto_* on QSK1 is likely at the protein level rather than transcriptionally. The absence of QSK1 also significantly reduces HopF2*_Pto_*'s ability to decrease *FLS2* and *PROSCOOP8* transcripts and inhibit ROS production upon treatment with flg22 (Fig. 10, C and D), SCOOP12, elf18, and pep2 (Supplementary Fig. S21).

To understand this functional relationship during infection, we introduced HopF2*_Pto_*-HA into the *Pto* DC3000 strain and subsequently infected both Col-0 and *qsk1-1* mutant. At 24 h postinoculation, HopF2*_Pto_*-HA accumulated more in Col-0 than in the *qsk1-1* mutant (Fig. 10E), while FLS2 levels were lower in Col-0 relative to the *qsk1-1* mutant when infected with *Pto* DC3000 harboring *HopF2_Pto_-HA*. Importantly, bacterial titers remained consistent between Col-0 and *qsk1-1* mutants at this time point (Supplementary Fig. S22). These findings strengthen our hypothesis that QSK1 is necessary for maintaining HopF2*_Pto_*'s protein stability and its ability to diminish FLS2 protein during infection. The *qsk1-1* mutant was more resistant against *Pto* DC3000 *ΔhopF2 HopF2_Pto_-HA* than Col-0 at 3 dpi, further supporting the importance of QSK1 in stabilizing and facilitating HopF2*_Pto_*'s function during infection (Fig. 10F).

## Discussion

In this study, we addressed the critical need for plants to precisely control the activity of PRR complexes, a safeguard against the detrimental outcomes of unexpected or excessive immune activation. We discovered QSK1 as a modulator of these complexes, primarily through its influence on the abundance of PRR proteins. Notably, our findings reveal an interaction between the Type III effector HopF2*_Pto_* and QSK1, which is pivotal for the stabilization of HopF2*_Pto_* within plants. Once stabilized by QSK1, HopF2*_Pto_* effectively inhibits SCOOP phytocytokine signaling and downregulates the cell's responses to PAMPs, DAMPs, and phytocytokines by reducing the accumulation of their respective PRRs (Supplementary Fig. S23).

### QSK1 associates with PRR-RBOHD complex

We observed that RBOHD forms complexes with EFR and FLS2 in the resting state, and this interaction remains unchanged upon PAMP treatment. Given that QSK1 was coimmunoprecipitated with EFR, FLS2, and RBOHD, it suggests that QSK1 could associate with EFR-RBOHD and FLS2-RBOHD complexes. Additionally, QSK1 associates with BAK1 in the resting state (Supplementary Fig. S24), indicating QSK1's involvement in multiple protein complexes. However, we cannot rule out the possibility that QSK1 might not directly interact with EFR, FLS2, or RBOHD but may exist in the same nanodomains at the plasma membrane. Previous studies have shown that FLS2 and its associated components are localized in REM1.2-positive nanodomains at the plasma membrane (Bucherl et al. 2017). These nanodomains are thought to accumulate in DRMs upon flg22 treatment (Keinath et al. 2010). Indeed, our co-IP with EFR, FLS2, or RBOHD and LC-MS/MS analyses identified proteins that accumulate in DRMs in response to flg22, including QSK1, REM1.2, ACA10, SYP71, HIR1, and HIR4 (Fig. 1B). Additionally, rice (*Oryza sativa*) RBOHB was shown to exist in DRMs in rice (Nagano et al. 2016). These results suggest our co-IP may isolate not only PRR-containing protein complexes but also components in immunity-specific nanodomains at the plasma membrane.

### QSK1 negatively regulates PTI through modulation of PRR protein levels

Phylogenetic analysis of QSK1, based on the kinase domain, demonstrates that QSK1 is widely conserved across most tracheophytes (Supplementary Fig. S25) (Ngou et al. 2024). FLS2 is also thought to be widely conserved in most angiosperms and possibly in gymnosperms (Albert et al. 2010). This relatively similar conservation pattern suggests that QSK1-mediated FLS2 regulation might be conserved across species. Indeed, a tomato (*Solanum lycopersicum*) homolog of QSK1, TOMATO ATYPICAL RECEPTOR-LIKE KINASE 1 (TARK1), acts as a negative regulator of immunity, as shown by increased resistance to pathogens in *tark1*-knockout lines and enhances susceptibility in its overexpression lines (Guzman et al. 2020). In *Arabidopsis*, QSK1-like proteins, LRR1, RECEPTOR-LIKE KINASE 1 (RKL1), and RECEPTOR-LIKE KINASES (RLK902) may also modulate PTI (Supplementary Fig. S26), as supported by elevated ROS production in *lrr1* and *rkl1* mutants in response to flg22, elf18, and pep2, a phenotype shared with the *qsk1-1* mutant. These results suggest that these homologs may function redundantly with QSK1 in PTI.

QSK1, also known as AUXIN-INDUCED LRR KINASE 1 and KINASE 7, influences channels and transporters through phosphorylation. For example, it activates the TPK1 potassium channel during stomatal closure (Isner et al. 2018) and modifies ABC TRANSPORTER G FAMILY MEMBER 36 (ABCG36), affecting the export of the auxin precursor indole-3-butyric acid and the phytoalexin camalexin (Aryal et al. 2023). Additionally, QSK1 regulates the activity of ARABIDOPSIS PLASMA MEMBRANE H^+^-ATPase 2 (AHA2) during low nitrated conditions. Low nitrate condition promotes QSK1 phosphorylation and induces ternary complex formation of QSK1, NITRATE TRANSPORTER1.1, and AHA2. This results in specific phosphorylation at inhibitory phosphorylation sites on AHA2, repressing proton efflux and nitrate-dependent lateral root growth (Zhu et al. 2024). Moreover, QSK1 is also involved in drought stress responses (Chen et al. 2021) and the regulation of callose-mediated plasmodesmata regulation and lateral root development during osmotic stress (Grison et al. 2019). Our study demonstrated an additional role for QSK1 in PTI regulation by modulating PRR abundance, distinct from its known pathways. QSK1 also functions as a coreceptor of SUCROSE-INDUCED RECEPTOR KINASE 1 (SIRK1), facilitating the phosphorylation and activation of aquaporin PLASMA MEMBRANE INTRINSIC PROTEIN 2;4 upon recognition of endogenous pep7 peptides (Wu et al. 2019; Wang et al. 2022). Our experiments showed no significant impact of this pathway on PTI responses (Supplementary Fig. S27), suggesting that FLS2 modulation by QSK1 does not depend on the pep7-SIRK1 signaling pathway.

### Mechanisms of QSK1-mediated FLS2 reduction

We observed that *QSK1* overexpression leads to a reduction of FLS2 protein levels at the plasma membrane (Fig. 5). Additionally, ConA inhibits the QSK1-mediated reduction of both EFR and FLS2, implying that QSK1 induces the vacuolar degradation of the PRRs through autophagy or endocytosis (Fig. 5H). This finding aligns with recent studies showing that the LRR-RK ROOT MERISTEM GROWTH FACTOR 1 INSENSITIVE (RGI) recognizes the phytocytokine GOLVEN2 (GLV2) and interacts with FLS2, enhancing its protein levels (Stegmann et al. 2022). The importance of RGI for the control of FLS2 protein levels is supported by the fact that *rgi1/2/3/4/5* quintuple mutant shows impaired FLS2 accumulation. Interestingly, the RGI3 ectodomain directly interacts with that of QSK1 and RLK902, and the RGI4 ectodomain interacts with that of RKL1 in vitro (Smakowska-Luzan et al. 2018). The interaction between QSK1, RGIs, and their homologs might imply a complex interplay that disrupts the GLV2-mediated interaction between RGI and FLS2, potentially leading to the degradation of GLV2-unbound FLS2 (Stegmann et al. 2022). This hypothesis is supported by the observation that the kinase activity of QSK1 is dispensable for the negative regulation of PTI (Supplementary Fig. S12) and that the strength of QSK1-mediated negative regulation depends on QSK1 abundance. Additionally, QSK1-mediated negative regulation might be upregulated following PAMP recognition, as PAMPs increase QSK1 transcript levels (Supplementary Fig. S8), suggesting that QSK1 may contribute to the shutdown of PTI after its activation. A comprehensive understanding of the intricate relationship between phytocytokine signaling, peptide hormone signaling, and FLS2 homeostasis, especially QSK1's involvement, remains a critical area for future research.

### HopF2*_Pto_* decreases plant responsiveness to PAMPs, DAMPs, and SCOOP phytocytokines by reducing PRR levels

Previous studies have established HopF2*_Pto_* as a potent inhibitor of PTI responses such as ROS production, MAPK activation, and callose deposition (Wu et al. 2011; Hurley et al. 2014; Zhou et al. 2014). Our work shows an additional role for HopF2*_Pto_* in diminishing plant response to PAMPs, DAMPs, and SCOOP phytocytokines specifically through reducing PRR levels and *PROSCOOPs* transcript levels. Interestingly, HopU1, another effector encoding a MARylation enzyme from *Pto* DC3000, also modulates FLS2 protein levels, by targeting GLYCINE-RICH RNA-BINDING PROTEIN 7, an RNA-binding protein in FLS2 translation (Fu et al. 2007; Nicaise et al. 2013). Unlike HopU1, which does not affect steady-state FLS2 levels (Nicaise et al. 2013), HopF2*_Pto_* significantly reduces both baseline (Fig. 6, C and D) and postinfection FLS2 levels (Fig. 7B). Thus, *Pto* DC3000 employs these 2 distinct MARlyation enzyme-coding effectors to manipulate FLS2 regulation in various ways. Furthermore, the pathogen uses the ubiquitin ligase AvrPtoB to degrade FLS2 by polyubiquitinating its kinase domain (Goehre et al. 2008). These strategies collectively highlight the significance of PRR suppression in the virulence mechanism of pathogens like *Pto* DC3000.

### The interplay of HopF2*_Pto_* with MIK2 and PRR expression in modulating plant immunity responses

HopF2*_Pto_* significantly reduces the transcript levels of important *PRRs*, including *FLS2*, *LORE*, *CANNOT RESPONSE TO DMBQ 1* (a RK required for perception of quinone and hydrogen peroxide), *RESISTANT TO DFPM INHIBITION OF ABA SIGNALING 2* (*RDA2*, a PRR for 9-methyl sphingoid base in fungal cerebroside), and *MIK2*, as well as a majority of *PROSCOOPs*. Intriguingly, *mik2* mutants exhibit reduced flg22-triggered ROS production (Rhodes et al. 2021), hinting at MIK2's role in maintaining baseline expression of *FLS2* and *PROSCOOPs*, through subtle activation by SCOOP peptides. This is further supported by the findings that MIK2 activation by SCOOP12 increases *FLS2* and *PROSCOOP* transcripts (Hou et al. 2021). Therefore, HopF2*_Pto_*'s impact on FLS2 levels might involve disrupting this MIK2-dependent positive feedback loop. However, the HopF2*_Pto_*-induced reduction in FLS2 cannot be solely attributed to MIK2 disruption. This is evident as HopF2*_Pto_* expression completely inhibits flg22-induced responses, whereas *mik2* mutants, although weaker, still retain some responsiveness to flg22 (Supplementary Figs. S19 and S20) (Rhodes et al. 2021).

### Distinct mechanisms of PRR degradation by HopF2*_Pto_*: exploring vacuolar degradation and transcript regulation

ConA's inhibition of the *HopF2_Pto_*-induced EFR reduction implies that HopF2*_Pto_* might target EFR for vacuolar degradation. However, ConA does not reverse HopF2*_Pto_*'s reduction of FLS2 protein (Supplementary Fig. S16A), possibly attributed to HopF2*_Pto_*'s differential effects on their transcripts: steady-state *FLS2* transcripts are diminished, while *EFR* transcripts remain unaffected. Consequently, even if ConA inhibits the vacuolar degradation of FLS2, the diminished levels of *FLS2* transcripts may still limit its protein synthesis. In contrast, EFR protein loss under HopF2*_Pto_* might be mainly through vacuolar degradation. This distinction is highlighted by the more pronounced reduction of FLS2 and FLS2-mediated MAPK activation than EFR by HopF2*_Pto_* (Figs. 6, C and D and 9; Supplementary Fig. S20). Previous studies have shown that signaling-inactive FLS2 undergoes degradation through selective autophagy with Orosomucoid (ORM) proteins as key autophagy receptors (Yang et al. 2019), while signaling-active FLS2 undergoes vacuolar degradation through endocytosis (Robatzek et al. 2006; Beck et al. 2012; Mbengue et al. 2016). HopF2*_Pto_* might exploit either pathway to diminish PRR protein levels. Despite our hypotheses, direct observation of EFR or FLS2 within autophagosomes or endosomes after expressing *HopF2_Pto_* was not feasible, likely due to the low expression levels of *EFR-GFP* in our *Arabidopsis* transgenic lines (*pEFR:EFR-GFP*) and the reduced *FLS2* transcript levels complicating detailed microscopic observation of FLS2-GFP in *pFLS2:FLS2-GFP* lines.

### MARylation activity of HopF2*_Pto_* is required for its virulence

Our finding establishes the critical role of HopF2*_Pto_*'s catalytic residue in MARylation for FLS2 protein reduction (Fig. 7). However, the exact mechanisms through which HopF2*_Pto_* influences transcriptome reprogramming changes and vacuolar degradation of PRRs via MARylation remain elusive. Previous studies have demonstrated that HopF2*_Pto_* targets key regulators of the PTI signaling pathway, such as MKK5 and BAK1 (Wang et al. 2010; Zhou et al. 2014; Han et al. 2024), as well as RPM1-INTERACTING4 (Wilton *et al.* 2010), impacting both PTI and ETI. It is plausible that HopF2*_Pto_*-mediated inhibition of MKK5 and BAK1 contributes to transcriptome reprogramming, possibly by disrupting MIK2 activation by SCOOP peptides (Hou et al. 2021; Rhodes et al. 2021; Yang et al. 2023). Additionally, HopF2*_Pto_*-mediated inhibition of MKK5 and BAK1 may also lead to the inhibition of many other peptide hormone signaling pathways, such as that induced by INFLORESCENCE DEFICIENT IN ABSCISSION (IDA)/IDA-LIKEs, C-TERMINALLY ENCODED PEPTIDEs, and RGIs, which might be involved in maintaining baseline expression of PRRs (Stegmann et al. 2022; Lalun et al. 2024; Rzemieniewski et al. 2024). Moreover, it is possible that HopF2*_Pto_* might reduce the protein levels of peptide hormone receptors, possibly through vacuolar degradation similar to EFR to shut down peptide hormone signaling pathways and reduce the expression of PRRs.

### Possible mechanisms of PRR degradation by HopF2*_Pto_*

MKK5 and BAK1 are unlikely candidates for the induction of HopF2*_Pto_*-mediated autophagy and/or endocytosis of PRRs, because both proteins are not part of a stable PRR complex in the absence of PAMP treatment (Chinchilla et al. 2007). Instead, HopF2*_Pto_* may MARylate other proteins to induce autophagy and/or endocytosis of PRRs.

We propose several hypotheses for HopF2*_Pto_* induction of PRR degradation. Firstly, HopF2*_Pto_* may MARylate and activate QSK1. This activation could inhibit RGI-FLS2 association, leading to PRR destabilization and their subsequent degradation through autophagy and/or endocytosis. This hypothesis is supported by the fact that both QSK1 and HopF2*_Pto_* induce vacuolar degradation of PRRs (Figs. 5, 6, and 9). Another hypothesis is that HopF2*_Pto_* directly MARylates PRRs, altering their structural conformation to enhance ORM protein binding and thus autophagy. In this scenario, QSK1 might serve as a scaffold, facilitating PRR MARylation. Lastly, HopF2*_Pto_* might target heteromeric G proteins, known to inhibit FLS2 autophagy (Miller et al. 2019). This is supported by the fact that bacterial toxins predominantly MARylate Gα proteins in animals (Ishiwata-Endo et al. 2020). Detecting HopF2*_Pto_*'s MARylation in vivo remains technically challenging, particularly direct observation of the MARylation of QSK1 and PRRs. Future studies should focus on identifying proteins MARylated by HopF2*_Pto_* in vivo and clarifying their roles in the vacuolar degradation of PRRs through autophagy and/or endocytosis.

### HopF2*_Pto_* requires QSK1 for its stabilization and functions

Our findings indicate that QSK1 plays a pivotal role in stabilizing HopF2*_Pto_* in plants, although its exact mechanism remains elusive. Notably, HopF2*_Pto_* is known to possess a predicted myristoylation sequence essential for plasma membrane localization and virulence (Wilton et al. 2010). This stabilization seems to occur when HopF2*_Pto_* interacts with QSK1 following myristoylation, potentially assisting HopF2*_Pto_* in targeting the PRR complex.

We found *qsk1-1* is more resistant, but *p35S:QSK1-3xHA#2* is less resistant to *Pto* DC3000 *ΔhopF2 HopF2_Pto_-HA* (Fig. 10F), suggesting the important function of QSK1 for HopF2*_Pto_* during infection. However, *qsk1-1* and *p35S:QSK1-3xHA#2* are similarly susceptible to *Pto* DC3000 compared to Col-0 (Supplementary Fig. S28). This difference in resistance may be attributed to the native HopF2*_Pto_* possessing an ATA start codon, which limits its expression. Therefore, the contribution of HopF2*_Pto_* is not as significant in *Pto* DC3000 wild type relative to *Pto* DC3000 *ΔhopF2 HopF2_Pto_-HA* (with ATG start codon). This also suggests that QSK1 may be more important for other *Pseudomonas* bacteria such as *P. syringae* pv. *phaseolicola* and *P. syringae* pv. *delphinii* whose HopF2 genes have ATG start codon (Tsiamis et al. 2000; Deng et al. 2003; Shan et al. 2004).

The complex interplay between QSK1 and HopF2*_Pto_*, while not fully understood, indicates a broader role for QSK1 and its homologs in aiding virulence effectors across various plants. For instance, XopN, a virulence factor from *X. campestris*, interacts with TARK1, a tomato homolog of QSK1 (Kim et al. 2009; Guzman et al. 2020). In *TARK1*-silenced plants, XopN's virulence function is notably reduced, suggesting that TARK1 is crucial for XopN functionality. Moreover, TARK1 may guide XopN to interact with tomato 14-3-3 isoform TOMATO FOURTEEN-THREE-THREE 1, a positive regulator of PTI in tomatoes (Taylor et al. 2012). This relationship mirrors that of HopF2*_Pto_*-QSK1-PRR, although it remains unclear if TARK1 primarily maintains XopN protein stability and facilitates its integration into the PRR complex.

## Materials and methods

### Plant materials and growth conditions

*Arabidopsis* (*A. thaliana*) ecotype Col-0 plants were grown on soil under an 8 or 16 h photoperiod at 23 °C, or in a half-strength MS medium containing 1% (*w*/*v*) sucrose under a continuous light photoperiod at 23 °C. *N. benthamiana* plants were soil grown under a 16 h photoperiod at 25 °C. The light is provided by light-emitting diodes (85 to 90 *μ*E m^−2^ s^−1^ for *N. benthamiana* and 65 to 75 *μ*E m^−2^ s^−1^ for *Arabidopsis*). The humidity was maintained at 60% to 70%.

### Vector construction and generation

To generate epiGreenB5-*p35S:QSK1-3×HA* and epiGreenB5-*p35S:QSK1-GFP*, the coding sequence region of QSK1 was amplified by PCR with KoD FX neo (Toyobo, Osaka, Japan), and the resulting PCR product was cloned into the epiGreenB5 (3*x*HA) and epiGreenB (eGFP) vectors between the *Cla*I and *Bam*HI restriction sites with an In-Fusion HD Cloning Kit (Clontech, CA, USA) (Nekrasov et al. 2009). To generate epiGreenB5-*pQSK1:QSK1-GFP,* an amplicon containing the 2,000-bp promoter upstream of the start codon and the coding regions of *QSK1* was cloned into the epiGreenB (eGFP) vectors between the *EcoRI* and *Bam*HI restriction sites with In-Fusion HD Cloning Kit. pCAMBIA2300-*pFLS2:FLS2-GFP* was described previously (Robatzek et al. 2006).

### Transgenic lines and T-DNA insertion lines

*Arabidopsis* stable transgenic lines of *p35S:QSK1-3×HA* (epiGreenB5), *p35S:QSK1-GFP* (epiGreenB5), and *qsk1-1/pQSK1:QSK1-GFP* (epiGreenB5) were generated by the floral drop and floral dip methods. T-DNA insertion mutant lines, *qsk1-1* (SALK_ 019840C), *lrr1* (WiscDsLoxHs082_03E), *rkl1* (SALK_099094C), *sirk1* (SALK_125543C), and *pep7* (SALK_025824C) were obtained from the Arabidopsis Biological Resource Center at the Ohio State University. Previously published lines were as follows: *bak1-5 bkk1* (Roux et al. 2011), *fls2*, *pFLS2:FLS2-GFP* (Robatzek et al. 2006), *efr-1/pEFR:EFR-GFP*, *rbohD/pRBOHD:3xFLAG-gRBOHD* (Kadota et al. 2014), *pDEX:HopF2_Pto_-HA*, and its variant D175A (Wilton et al. 2010). Homozygous *pFLS2:FLS2-GFP/p35S:QSK1-3xHA*, *pEFR:EFR-GFP/p35S:QSK1-3xHA*, *pDEX:HopF2_Pto_-HA/qsk1-1*, *pDEX:HopF2_Pto_-HA/pFLS2:FLS2-GFP*, and *pDEX:HopF2_Pto_*-HA*/pEFR:EFR-GFP* lines were generated by crossing homozygous lines and then selection by genotyping.

### Generation of QSK1 antibody

A polyclonal anti-QSK1 antibody was produced by immunizing rabbits with a synthetic peptide (NH2-C + EEVSHSSGSPNPVSD-COOH) originating from the C-terminal region of QSK1 (Eurofins Scientific SE, Luxembourg).

### Immunoblotting

Immunoblotting was performed with antibodies diluted in the blocking solution (5% [*w*/*v*] nonfat milk in TBS with 0.1% [*v*/*v*] Tween) at the following dilutions: α-GFP antibody (ab290, Abcam, Cambridge, UK), 1:8,000; α-HA-horseradish peroxidase (HRP) (3F10, Roche, Basel, Switzerland), 1:5,000; α-FLAG-HRP (M2 monoclonal antibody, Sigma-Aldrich, St. Louis, MO, USA), 1:2,000; α-FLS2 (Chinchilla et al. 2006), 1:1,000; α-BAK1 (Roux et al. 2011), 1:1,000; α-QSK1, 1:500; and α-rabbit-HRP conjugated antibody (NA934; GE Healthcare, Chicago, IL, USA), 1:10,000. For detection of RBOHD and EFR-GFP, α-RBOHD (AS152962; 1:1,000; Agrisera, Vännäs, Sweden) antibody and α-GFP antibody (ab290, Abcam, Cambridge, UK) were diluted in Can Get Signal Solution 1 (Toyobo, Osaka, Japan) and the α-rabbit-HRP conjugated antibody was diluted in Can Get Signal Solution 2 to enhance the signal of immunoblotting.

### Bacterial strains

*Pto* DC3000 *ΔhopF2 HopF2_Pto_-HA* was described previously (Wilton et al. 2010). It is important to note that the native HopF2*_Pto_* has an ATA start codon, which limits its expression. On the other hand, *Pto* DC3000 *ΔhopF2 HopF2_Pto_-HA* uses the more common ATG start codon, resulting in enhanced expression of *HopF2_pto_-HA* during the infection. To generate *P. fluorescens* (Pf0-1) *HopF2_Pto_-HA* and *P. fluorescens* Pf0-1*HopF2_Pto_* (D175A)-*HA*, *P. fluorescens* Pf0-1 was transformed with the expression vectors, *schF2/hopF2_Pto_  ^ATG^:HA* or *schF2/hopF2_Pto_  ^ATG^* (D175A)*:HA.*

### Statistical analysis

Statistical significances based on *t*-test and 1-way ANOVA were determined with GraphPad Prism6 software (GraphPad Software, San Diego, CA, USA). Statistical data are provided in Supplementary Data Set S11.

### Other methods

Chemical inhibitors were described in Supplementary Methods S1. Protein extraction, IP, protein identification by LC-MS/MS, ROS burst assay, MAPK activation assay, bacterial infection assays, phylogenetic analyses, transient expression in *N*. *benthamiana,* confocal microscopy analyses, RT-qPCR assay, QIS-seq analyses, RNA-seq and differential gene expression analyses, PCA with SOM clustering, and GO term enrichment analyses were performed as described previously (Lewis et al. 2012; Kadota et al. 2014; Goto et al. 2020, 2024) with minor modifications detailed in Supplementary Methods S1. All primers used in this study are listed in Supplementary Data Set S12.

### Accession numbers

*Arabidopsis* genes studied can be found in the TAIR database (https://www.arabidopsis.org) under the following accession numbers: QSK1 (AT3G02880), RBOHD (AT5G47910), EFR (AT5G20480), FLS2 (AT5G46330), BAK1 (AT4G33430), MIK2 (AT4G08850), PROSCOOP8 (AT5G44575), LRR1 (AT5G16590), RKL1 (AT1G48480), and RLK902 (AT3G17840), SIRK1 (AT5G10020), and PEP7 (AT5G09978). Sequence data for the bacterial protein HopF2*_Pto_* can be found in the EMBL database under the accession number AAO54046.

## Supplementary Material

koae267_Supplementary_Data

## Data Availability

The data underlying this article are available in the article and in its online Supplementary material.

## Supplementary data

The following materials are available in the online version of this article.

**Supplementary Figure S1.** Heterologous expression of *QSK1-3xHA* reduces flg22-induced ROS production in *N. benthamiana.*

**Supplementary Figure S2.** T-DNA insertion and expression in the *qsk1-1* mutant.

**Supplementary Figure S3.** Growth of *Pto* DC3000 *hrcC^−^* in *qsk1-1* and *p35S:QSK1-3xHA* #2.

**Supplementary Figure S4.** Phenotype recovery in *qsk1-1* complementation lines.

**Supplementary Figure S5.**  *QSK1* overexpression lines are slightly smaller than Col-0 and the *qsk1-1* mutant.

**Supplementary Figure S6.** QSK1 does not affect chitin-induced ROS production.

**Supplementary Figure S7.** QSK1 localizes at the plasma membrane.

**Supplementary Figure S8.** flg22 and elf18 induce *QSK1* transcript accumulation.

**Supplementary Figure S9.** Quantification of PRR levels in *qsk1-1* and *p35S:QSK1-3xHA #2*.

**Supplementary Figure S10.** QSK1 does not affect RBOHD protein level.

**Supplementary Figure S11.** PRR protein levels after PAMP treatment in *qsk1-1* and *p35S:QSK1-3xHA #2*.

**Supplementary Figure S12.** The crucial residue for QSK1 kinase activity is not required for the negative regulation of FLS2.

**Supplementary Figure S13.** Pharmacological analyses of QSK1-induced FLS2 reduction.

**Supplementary Figure S14.** Quantification of PRR levels in *pDEX:HopF2-HA*.

**Supplementary Figure S15.** The DEX concentration dependency for the expression of *pDEX:HopF2_Pto_-HA* (wild type) and *pDEX:HopF2_Pto_-HA* (D175A).

**Supplementary Figure S16.** Pharmacological analyses of HopF2*_Pto_*-induced FLS2 reduction.

**Supplementary Figure S17.** Multidimensional scaling plot with SOM clustering of genes affected by HopF2*_Pto_*.

**Supplementary Figure S18.** HopF2*_Pto_* affects some transcript levels of commonly associated proteins with EFR, FLS2, and RBOHD.

**Supplementary Figure S19.** HopF2*_Pto_* inhibits PAMP-induced ROS production.

**Supplementary Figure S20.** HopF2*_Pto_* inhibits PAMP-induced MAPK activation.

**Supplementary Figure S21.** HopF2*_Pto_* requires QSK1 to inhibit PAMP-inducible ROS production.

**Supplementary Figure S22.** The population of *Pto* DC3000 *Δhopf2 HopF2_Pto_-HA* remains consistent between Col-0 and *qsk1-1* 24 h after syringe infiltration.

**Supplementary Figure S23.** A model of HopF2*_Pto_* virulence function in suppressing PTI and its relationship with QSK1.

**Supplementary Figure S24.** QSK1 associates with BAK1 in vivo.

**Supplementary Figure S25.** QSK1 homologs are widely conserved across most tracheophytes.

**Supplementary Figure S26.**  *lrr1* and *rkl1* mutants have higher ROS production in response to flg22, elf18, and pep2.

**Supplementary Figure S27.** SIRK1 and PEP7 do not affect PAMP-induced ROS production.

**Supplementary Figure S28.** Bacterial growth in *qsk1-1* and *p35S:QSK1-3xHA* lines.

**Supplementary Methods S1.** Additional methods.

**Supplementary Data Set S1_1.** FLS2-associated proteins.

**Supplementary Data Set S1_2.** EFR-associated proteins.

**Supplementary Data Set S1_3.** RBOHD-associated proteins.

**Supplementary Data Set S1_4.** Associated proteins shared amongst FLS2, EFR, and RBOHD.

**Supplementary Data Set S2_1.** Peptide counts of QSK1 in FLS2-GFP co-IP analysis.

**Supplementary Data Set S2_2.** Peptide counts of QSK1 in EFR-GFP co-IP analysis.

**Supplementary Data Set S2_3.** Peptide counts of QSK1 in FLAG-RBOHD co-IP analysis.

**Supplementary Data Set S3_1.** Enrichment score of HopF2*_Pto_* interactors in Quantitative interactor screening with next-generation sequencing (QIS-seq).

**Supplementary Data Set S3_2.** In planta proximity-dependent BioID of HopF2*_Pto_* associated proteins.

**Supplementary Data Set S4_1.** Normalized expression values of genes in *Arabidopsis* seedlings of Col-0 or *pDEX:HopF2_Pto_-HA* lines treated with DMSO or DEX.

**Supplementary Data Set S4_2.** Genes upregulated by DEX treatment compared to DMSO treatment in Col-0 (log2 fold change ≥ 1, FDR ≤ 0.05).

**Supplementary Data Set S4_3.** Genes downregulated genes by DEX treatment compared to DMSO treatment in Col-0 (log2 fold change ≤ −1, FDR ≤ 0.05).

**Supplementary Data Set S4_4.** Genes upregulated by the expression of *HopF2_Pto_-HA* (*pDEX:HopF2_Pto_-HA + DEX* vs Col-0 + DEX) (log2 fold change ≥ 1, FDR ≤ 0.05).

**Supplementary Data Set S4_5.** Genes downregulated by the expression of *HopF2_Pto_-HA* (*pDEX:HopF2_Pto_-HA* + DEX vs Col-0 + DEX) (log2 fold change ≤ −1, FDR ≤ 0.05).

**Supplementary Data Set S4_6.** Genes upregulated by DEX treatment in *pDEX:HopF2_Pto_-HA* (*pDEX:HopF2_Pto_-HA* + DEX vs *pDEX:HopF2_Pto_-HA* + DMSO) (log2 fold change ≥ 1, FDR ≤ 0.05).

**Supplementary Data Set S4_7.** Genes downregulated by DEX treatment in *pDEX:HopF2_Pto_-HA* (*pDEX:HopF2_Pto_-HA* + DEX vs *pDEX:HopF2_Pto_-HA* + DMSO) (log2 fold change ≤ −1, FDR ≤ 0.05).

**Supplementary Data Set S5_1.** GO enrichment analysis of the genes upregulated by HopF2*_Pto_*-HA.

**Supplementary Data Set S5_2.** GO enrichment analysis of the genes downregulated by HopF2*_Pto_*-HA

**Supplementary Data Set S5_3.** GO enrichment analysis of the genes in Cluster 1.

**Supplementary Data Set S5_4.** GO enrichment analysis of the genes in Cluster 2.

**Supplementary Data Set S6.** Genes in SOM clusters.

**Supplementary Data Set S7.** Phylogenetic tree annotation for Supplementary Fig. S25A.

**Supplementary Data Set S8.** Phylogenetic tree newick for Supplementary Fig. S25A.

**Supplementary Data Set S9.** Phylogenetic tree annotation for Supplementary Fig. S25B.

**Supplementary Data Set S10.** Phylogenetic tree newick for Supplementary Fig. S25B.

**Supplementary Data Set S11.** Results of statistical analysis.

**Supplementary Data Set S12.** Primers used in this paper.

## Dive Curated Terms

The following phenotypic, genotypic, and functional terms are of significance to the work described in this paper:

ACA10 Gramene: AT4G29900
ACA10 Araport: AT4G29900

AHA2 Gramene: AT4G30190
AHA2 Araport: AT4G30190

SOM Gramene: AT1G03790
SOM Araport: AT1G03790

SYP71 Gramene: AT3G09740
SYP71 Araport: AT3G09740

PLL4 Gramene: AT2G28890
PLL4 Araport: AT2G28890

PLL5 Gramene: AT1G07630
PLL5 Araport: AT1G07630

TPK1 Gramene: AT1G02880
TPK1 Araport: AT1G02880

At5g15400 Gramene: AT5G15400
At5g15400 Araport: AT5G15400

rkl1 Gramene: AT1G48480
rkl1 Araport: AT1G48480

PEPR1 Gramene: AT1G73080
PEPR1 Araport: AT1G73080

BIK1 Gramene: AT2G39660
BIK1 Araport: AT2G39660

lrr1 Gramene: LOC_Os06g47650
lrr1 Araport: LOC_Os06g47650

PTI Gramene: pattern-triggered immunity
PTI Araport: pattern-triggered immunity

ROS Gramene: reactive oxygen species
ROS Araport: reactive oxygen species

MKK5 Gramene: AT3G21220
MKK5 Araport: AT3G21220

BIR2 Gramene: AT3G28450
BIR2 Araport: AT3G28450

ETI Gramene: Effector-triggered immunity
ETI Araport: Effector-triggered immunity

QSK1 Gramene: AT3G02880
QSK1 Araport: AT3G02880
