# Supplementary material for: The leucine-rich repeat receptor kinase QSK1 regulates PRR-RBOHD complexes targeted by the bacterial effector HopF2Pto
Source: Plant Cell. 2024 Oct 21;36(12):4932–51. doi: 10.1093/plcell/koae267 (PMC11641854; doi:10.1093/plcell/koae267)
Supplement: koae267_Supplementary_Data [file koae267_supplementary_data.zip › Sup Figures and Methods_Final.pdf]

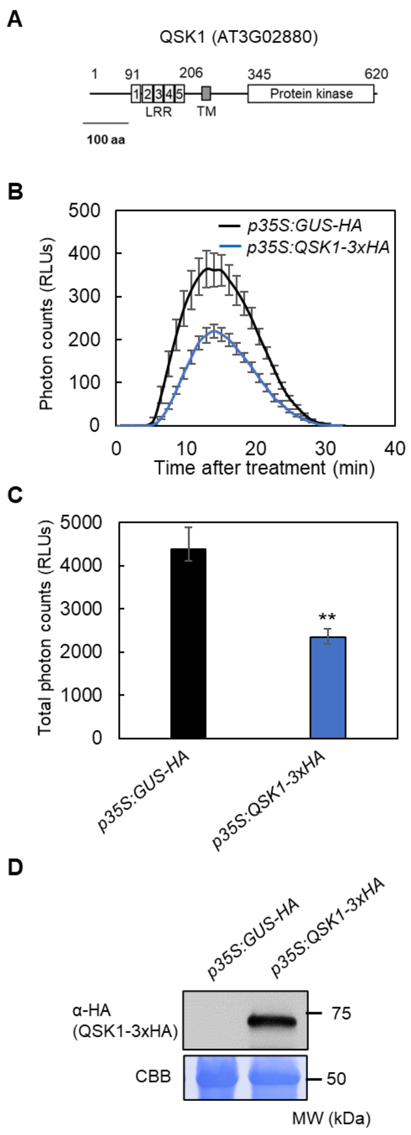

Supplementary Figure S1. Heterologous expression of *QSK1-3xHA* reduces flg22-induced ROS production in *Nicotiana benthamiana.* (Supports Figure 1)
A) QSK1 protein domain structure. **B and C)** QSK1-3xHA (*p35S:QSK1-3xHA*) and free GUS-HA (*p35S:GUS-HA*) were expressed in the same leaf by agroinfiltration, and flg22-induced ROS was measured three days later by a luminol-based assay , with results shown in relative luminescence units (RLUs). The time-course (**B**) and total amount (**C**) of ROS production induced by 1 μM flg22. Values are mean ± standard error (SE) from 8 leaf discs. Asterisks indicate a significant difference based on Student's t-test (**$p \leq 0.01$). **D)** The protein expression of *QSK1-3xHA* was confirmed by immunoblot analysis with α-HA antibody. Equal loading of protein samples is shown by Coomassie Brilliant Blue (CBB) staining. The position of the closest protein marker to the band is indicated, with its molecular weight (MW) shown in kilodaltons (kDa).The experiments were repeated three times with similar results.

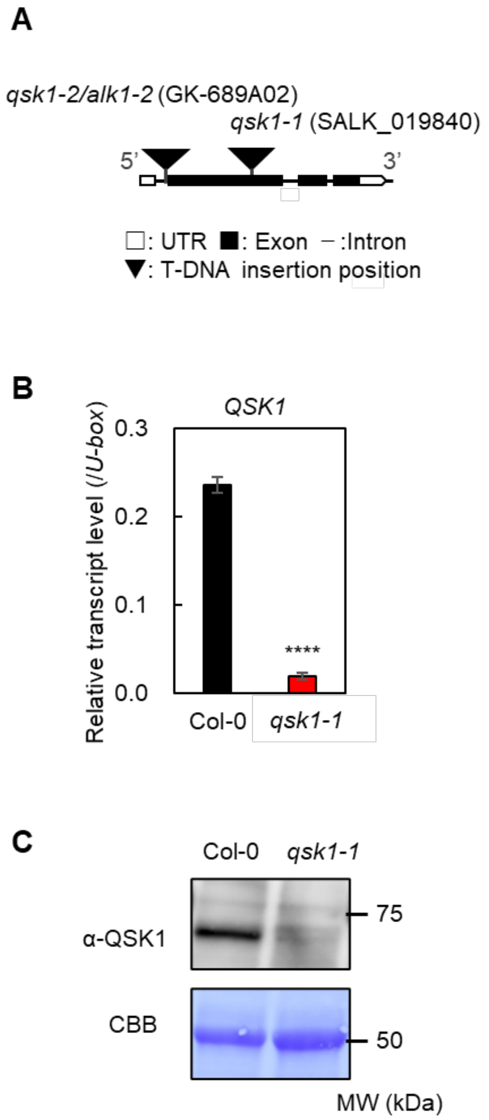

Supplementary Figure S2. T-DNA insertion and expression in *qsk1-1* mutant. (Supports Figure 3)
A) Positions of T-DNA insertions within the *QSK1* locus in *qsk1-1* mutant (SALK_019840). **B)** Transcript levels of *QSK1* in two-week-old seedlings of Col-0 and *qsk1-1* mutant were measured by RT-qPCR after normalization to the *U-box* housekeeping gene transcript (*At5g15400*). Values are mean ± SE from three different plants. Asterisks indicate a significant difference based on Student's t-test ($^{****}p \leq 0.001$). The data for *qsk1-1* are shown in red for clarity.**C)** The QSK1 protein level in two-week-old seedlings of Col-0 and *qsk1-1* mutant were measured by immunoblotting with α-QSK1 antibody. Equal loading of protein samples is shown by CBB staining. The experiments were repeated three times with similar results.

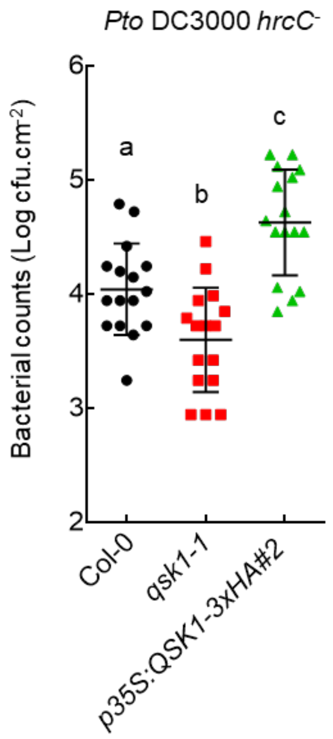

Supplementary Figure S3. Growth of *Pto* DC3000 *hrcC*$^-$ in *qsk1-1* and *p35S:QSK1-3xHA#2*. (Supports Figure 3 and Figure 4)
qsk1-1 mutant was more resistant to *Pto* DC3000 *hrcC*$^-$ mutant, but *p35S:QSK1-3xHA#2* line was more susceptible compared to Col-0. Values are means ± standard deviation from 16 plants. Different letters indicate significant differences at $p \leq 0.05$ (one-way ANOVA, Tukey's post hoc test). *Pto* DC3000 *hrcC*$^-$ were sprayed onto leaf surfaces of six-week-old soil-grown Arabidopsis plants at a concentration of $1x10^6$ cfu (colony-forming units)/mL. Three days post-spray-inoculation, leaves were harvested to determine bacterial growth. All the experiments were repeated three times with similar results. The data for *qsk1-1* and *p35S:QSK1-3xHA#2* are shown as red squares and green triangles, respectively for clarity.

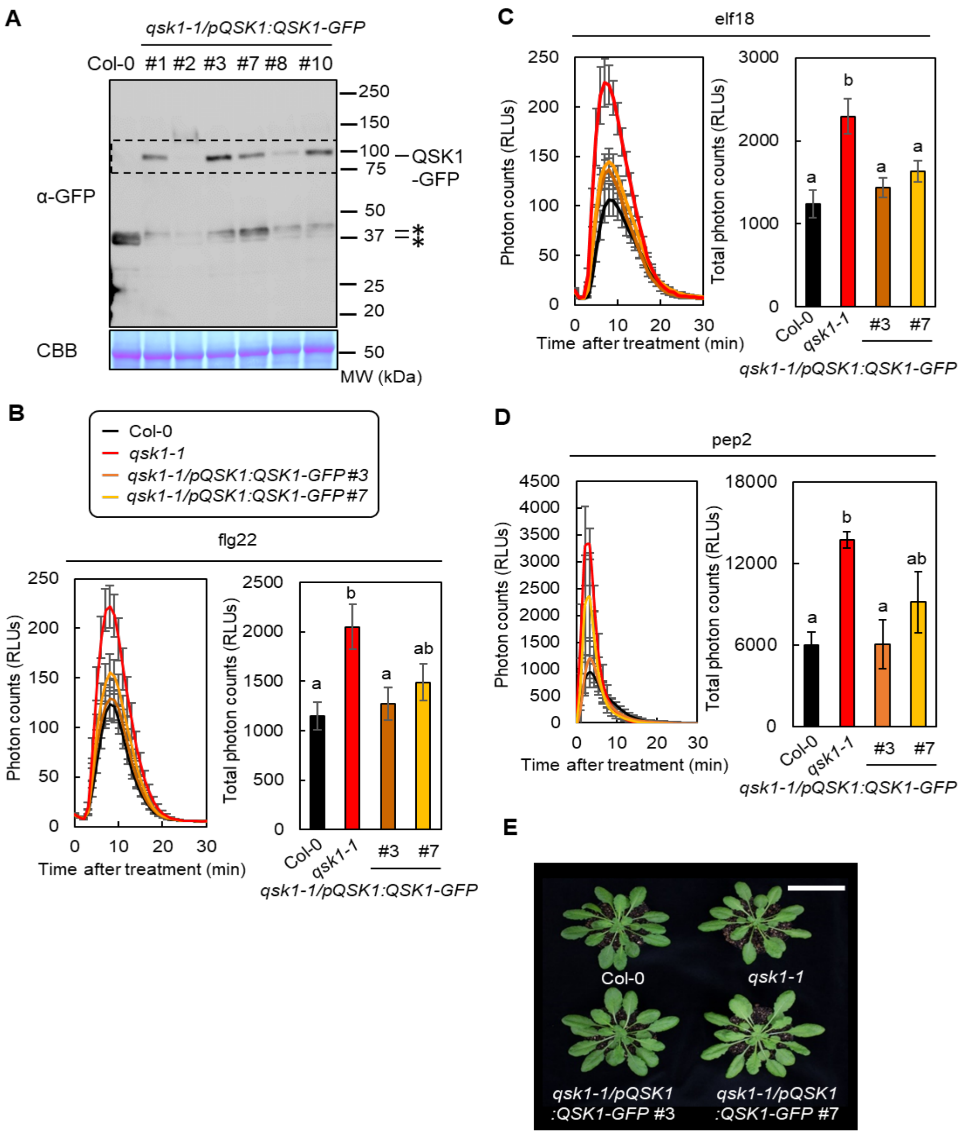

Supplementary Figure S4. Phenotype recovery in *qsk1* complementation lines. (Supports Figure 3)

A) The QSK1-GFP protein levels in two-week-old seedlings of *qsk1-1/pQSK1:QSK1-GFP* lines were measured by immunoblotting with α-GFP antibody. Equal loading of protein samples is shown by CBB staining. Asterisks indicate non-specific bands present also in Col-0. **B-D)** *qsk1-1/pQSK1:QSK1-GFP* lines induce a similar level of ROS production upon treatment with flg22 (**B**), elf18 (**C**), and pep2 (**D**) to Col-0. 16 leaf discs from four- to five-week-old Arabidopsis plants were treated with 1 µM flg22, 1 µM elf18, or 1 µM pep2, and the time-course (left), and total amount (right) of ROS production were measured by a luminol-based assay. Values are mean ± SE (n=16). Different letters indicate significant differences at $p \leq 0.05$ (one-way ANOVA, Tukey's post hoc test). The data of *qsk1-1/pQSK1:QSK1-GFP#3* and *#7* are shown in brown and yellow, respectively for clarity, while the data of *qsk1-1* is shown in red. **E)** The growth phenotype of six-week-old Col-0, *qsk1-1 mutant*, and *qsk1-1/pQSK1:QSK1-GFP* lines. The white bar represents 5 cm. The experiments were repeated three times with similar results.

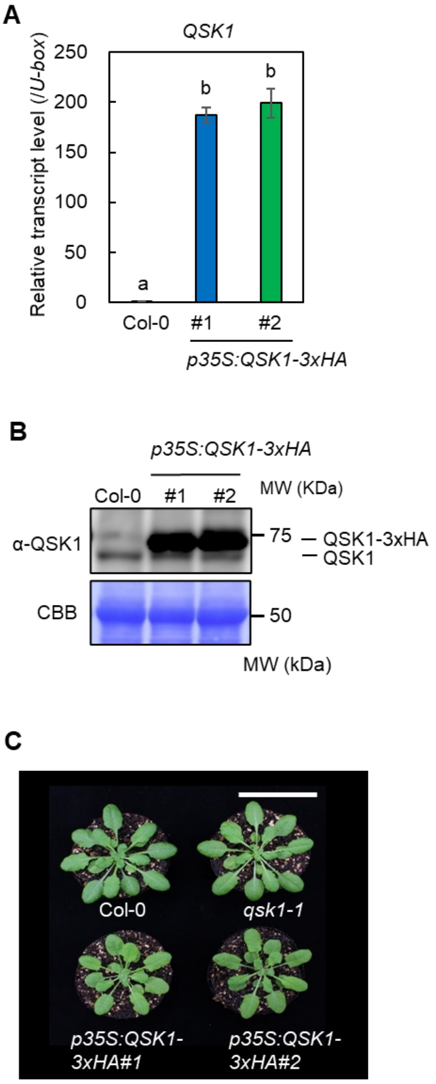

Supplementary Figure S5. *QSK1* overexpression lines are slightly smaller than Col-0 and *qsk1-1* mutant. (Supports Figure 4)
A) Transcript levels of *QSK1* in *p35S:QSK1-3×HA* lines. Transcript levels of *QSK1* in two-week-old seedlings of Col-0 and *p35S:QSK1-3×HA* lines were measured by RT-qPCR after normalization to the *U-box* housekeeping gene transcript (*At5g15400*). Values are mean ± SE from three different plants. Different letters indicate significant differences at $p \leq 0.01$ (one-way ANOVA, Tukey's post hoc test). The data of *p35S:QSK1-3xHA#1* and #2 are shown in light blue and green, respectively for clarity. **B)** QSK1 protein levels in *p35S:QSK1-3×HA* lines. QSK1 protein levels in two-week-old Arabidopsis seedlings of Col-0 and *p35S:QSK1-3×HA* lines were measured by immunoblotting with α-QSK1 antibody. Equal loading of protein samples is shown by CBB staining. **C)** *p35S:QSK1-3×HA* lines are slightly smaller than Col-0 and *qsk1-1 mutant*. The growth phenotype of six-week-old Col-0, *qsk1-1 mutant*, and *p35S: QSK1-3xHA* lines. The white bar represents 50 μm. The experiments were repeated three times with similar results.

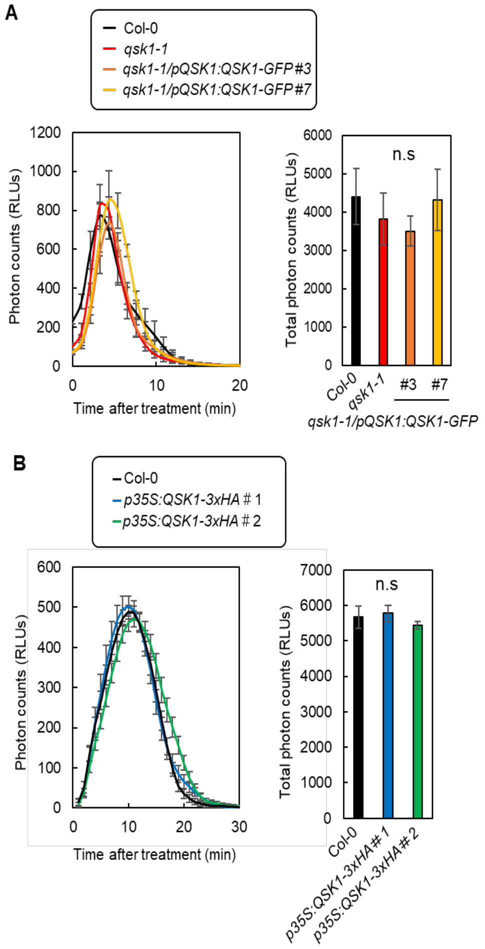

Supplementary Figure S6. QSK1 does not affect chitin-induced ROS production. (Supports Figure 3 and Figure 4)

A) *qsk1-1* and *qsk1-1/pQSK1:QSK1-GFP* lines induce similar levels of ROS production to Col-0 upon treatment with chitin. Eight leaf discs from four- to five-week-old Arabidopsis plants were treated with 10 μM $(GlucNAc)_7$, and the time-course (left) and total amount (right) of ROS production were measured by a luminol-based assay. Values are mean ± SE from three independent experiments. There is no significant (n.s) difference (n.s) at $p \leq 0.05$ (one-way ANOVA, Tukey's post hoc test). The data of *qsk1-1/pQSK1:QSK1-GFP#3* and *#7* are shown in brown and yellow, respectively for clarity, while the data of *qsk1-1* is shown in red. **B)** *p35S:QSK1-3xHA* lines induce similar level of ROS production to Col-0 upon treatment with chitin. Thirty two leaf discs from four- to five-week-old Arabidopsis plants were treated with 10 μM $(GlucNAc)_7$, and time-course (left) and the total amount (right) of ROS production were measured by a luminol-based assay. Values are mean ± SE from 32 leaf discs. There is no significant difference at $p \leq 0.05$ (one-way ANOVA, Tukey's post hoc test). The experiment was repeated three times with similar results. The data of *p35S:QSK1-3xHA#1* and *#2* are shown in light blue and green, respectively for clarity.

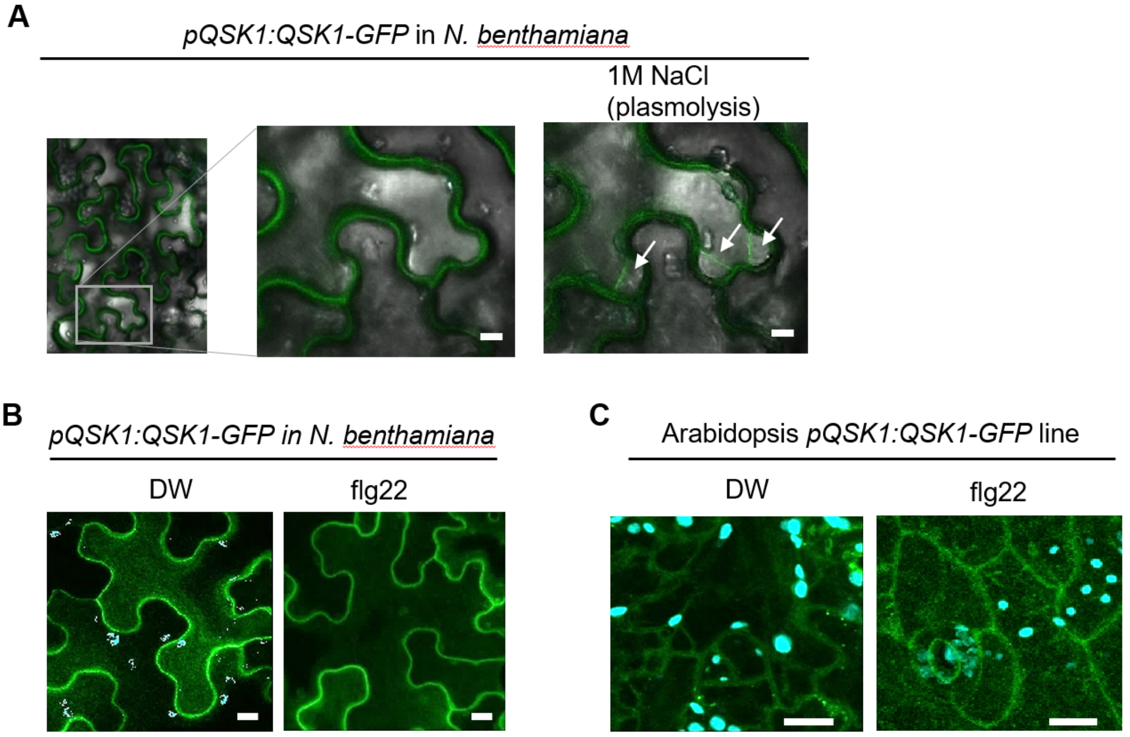

Supplementary Figure S7. QSK1 localizes at the plasma membrane. (Supports Figure 4)

A) Subcellular localization of QSK1-GFP expressed under the control of its native promoter (*pQSK1:QSK1-GFP*) in *N. benthamiana.* The localization of QSK1-GFP before or after plasmolysis was analyzed by confocal microscopy. The leaf disks were mounted in the presence of 1M NaCl for 10 min to induce plasmolysis. The white arrows indicate the position of the plasma membrane. All of t he white bars represent 5 µm. **B and C)** flg22 treatment does not change QSK1-GFP localization at the plasma membrane. The localization of QSK1-GFP expressed in *N. benthamiana* (**B**) and stable Arabidopsis *pQSK1:QSK1-GFP* line (**C**) was observed after treatment with 1 µM flg22 for 1 h. Two-week-old Arabidopsis seedlings of *pQSK1:QSK1-GFP* line were used for microscopic analysis. All of t he white bars represent 30 µm (B and C). The experiments were repeated three times with similar results.

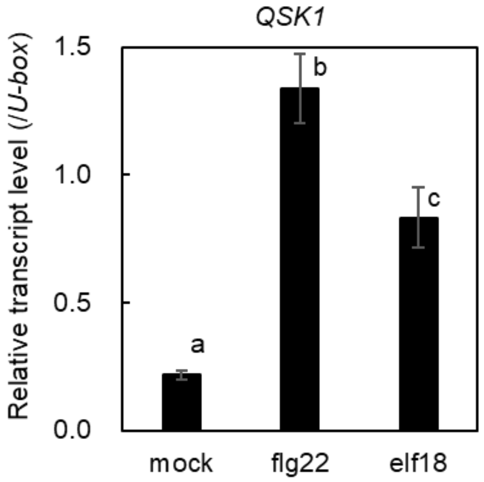

Supplementary Figure S8. flg22 and elf18 induce *QSK1* transcript accumulation (Supports Figure 4)

Transcript levels of *QSK1* in ten-day-old Arabidopsis seedlings after treatment with DW (mock), 1 μM flg22, or 1 μM elf18 for 6 h were measured by RT-qPCR after normalization to *U-box* housekeeping gene transcript (*At5g15400*). Values are presented as mean ± SE derived from three independent experiments, with each experiment utilizing three different plants. Different letters indicate significant differences based on one-way ANOVA and Tukey's post hoc test ($p \leq 0.05$). The experiments were repeated three times with similar results.

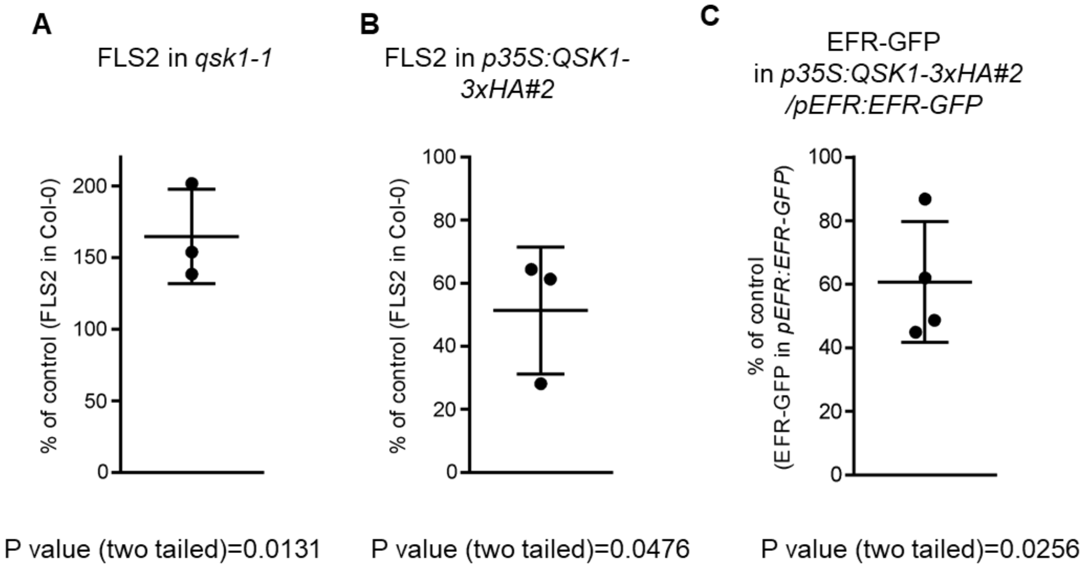

Supplementary Figure S9. Quantification of PRR levels in *qsk1-1* and *p35S:QSK1-3xHA#2*. (Supports Figure 5)
A) FLS2 protein level in *qsk1-1* compared to that in Col-0. **B)** FLS2 protein level in *p35S:QSK1-3xHA#2* compared to that in Col-0. **C)** EFR-GFP protein level in *p35S:QSK1-3xHA#2/pEFR:EFR-GFP* line compared to that in *pEFR:EFR-GFP*. FLS2 and EFR-GFP protein levels of two-week-old Arabidopsis seedlings were measured by immunoblotting with α-FLS2 and α-GFP antibodies, respectively. The signals of the bands were quantified with ImageJ software. Values are mean (center line) ± standard deviation (SD) of relative FLS2 or EFR-GFP protein levels compared to the control (FLS2 in Col-0 or EFR-GFP in *pEFR:EFR-GFP*) from three independent experiments for **A** and **B,** and four independent experiments for **C.** Individual data points are shown as dots. Statistical analysis was performed using a two-tailed one-sample t-test.

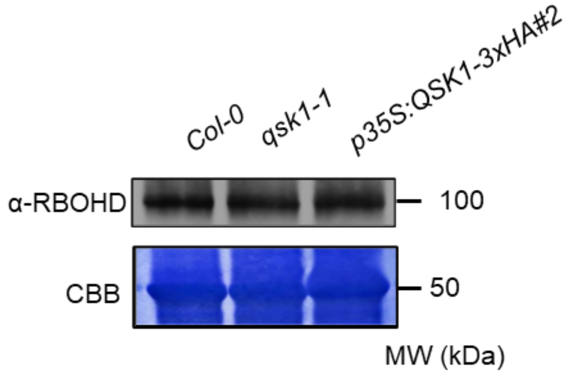

Supplementary Figure S10. QSK1 does not affect RBOHD protein level (Supports Figure 5)
RBOHD protein levels are similar in Col-0, *qsk1-1,* and *p35S:QSK1-3xHA* lines. RBOHD protein levels of two-week-old Arabidopsis seedlings were measured by immunoblotting with α-RBOHD antibody. Equal loading of protein samples is shown by CBB staining. The experiment was repeated three times with similar results.

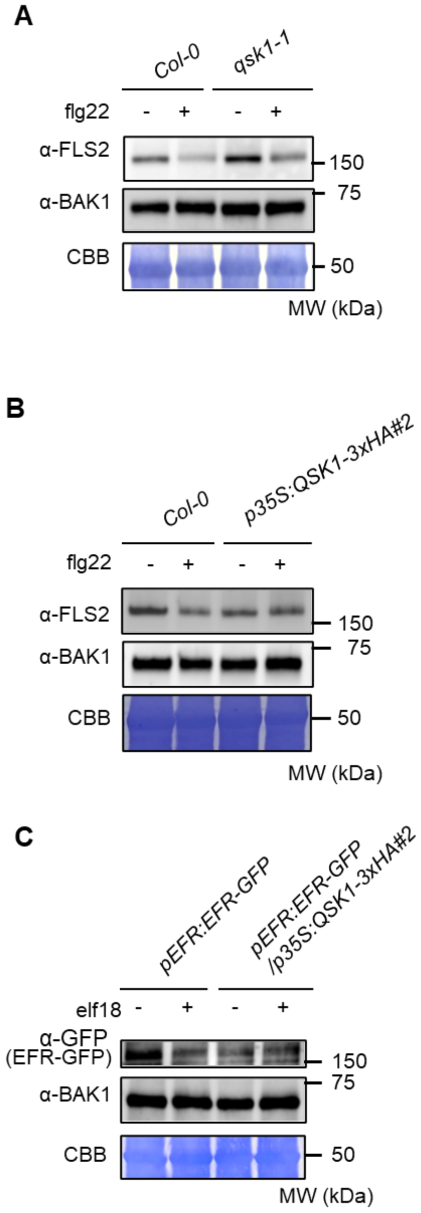

Supplementary Figure S11. PRR protein levels after PAMP treatment in *qsk1-1* and *p35S:QSK1-3xHA#2* (Supports Figure 5)

A) FLS2 protein levels after flg22 treatment in Col-0 and *qsk1-1*. **B**) FLS2 protein levels after flg22 treatment in Col-0 and *p35S:QSK1-3xHA#2* line. **C**) EFR-GFP protein levels after elf18 treatment in *pEFR:EFR-GFP* or *pEFR:EFR-GFP/p35S:QSK1-3xHA#2* line. FLS2, EFR-GFP, and BAK1 protein levels of two-week-old Arabidopsis seedlings after treatment with (+) or without (-) 10 μM flg22, or 10 μM elf18 for 1 h were measured by immunoblotting with α-RBOHD, α-GFP, and α-BAK1 antibodies. Equal loading of protein samples is shown by CBB staining. The experiments were repeated three times with similar results.

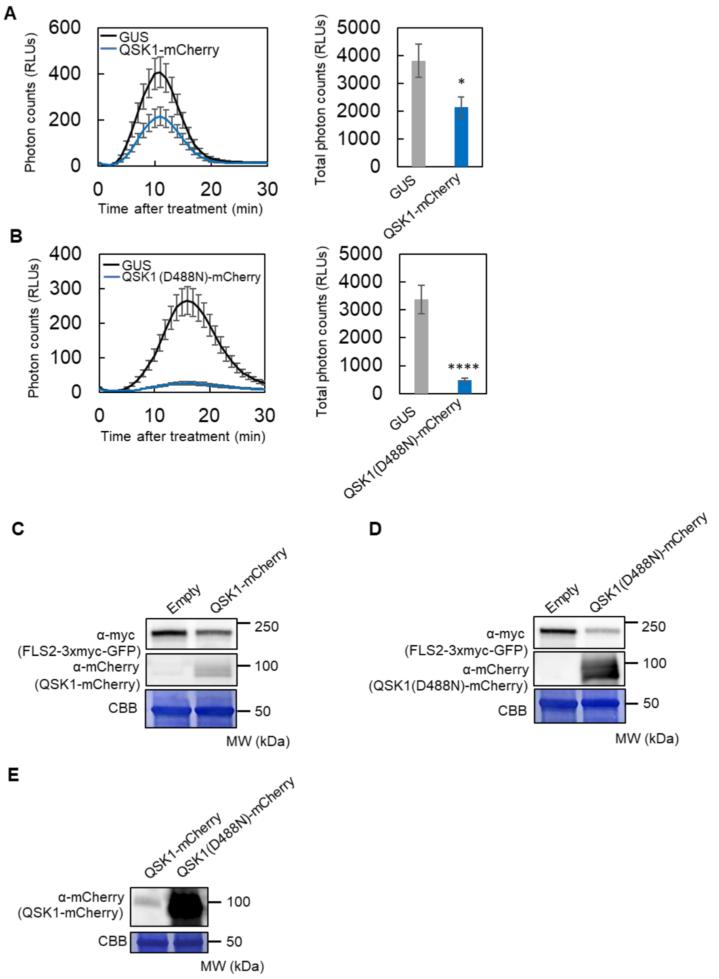

Supplementary Figure S12. The crucial residue for kinase activity of QSK1 is not required for the negative regulation of FLS2 (Supports Figure 5)

The expression of QSK1-mCherry (**A**) and QSK1(D488N)-mCherry (**B**) reduces flg22-induced ROS burst in N. benthamiana. QSK1-mCherry proteins and GUS (negative control) were transiently expressed in the same leaves under the control of p35S promoter, and flg22-induced ROS production was measured three days after agroinfiltration. The time-course (left) and total amount (right) of ROS production were measured by a luminol-based assay. Values are mean ± SE from 16 leaf discs. Asterisks indicate a significant difference based on Student's t-test (*p ≤ 0.05, ****p ≤ 0.001). The expression of QSK1-mCherry (**C**) and QSK1(D488N)-mCherry (**D**) reduces FLS2-3xmyc-GFP levels in N. benthamiana. FLS2-3xmyc-GFP and QSK1-mCherry proteins were transiently expressed under the control of p35S promoter, and their protein levels were measured three days after agroinfiltration by immunoblotting with α-mCherry and α-myc antibodies. For positive control, only FLS2-3xmyc-GFP was expressed in the same leaves, and Agrobacterium concentration (OD600=0.6) was adjusted with empty Agrobacterium. **E**) QSK1(D488N)-mCherry is expressed in a much higher amount than QSK1-mCherry in N. benthamiana. QSK1-mCherry and QSK1(D488N)-mCherry were transiently expressed in the same leaves under the control of p35S promoter, and the protein levels were measured three days after agroinfiltration by immunoblotting with α-mCherry antibody. Equal loading of protein samples is shown by CBB staining. The experiments were repeated three times with similar results.

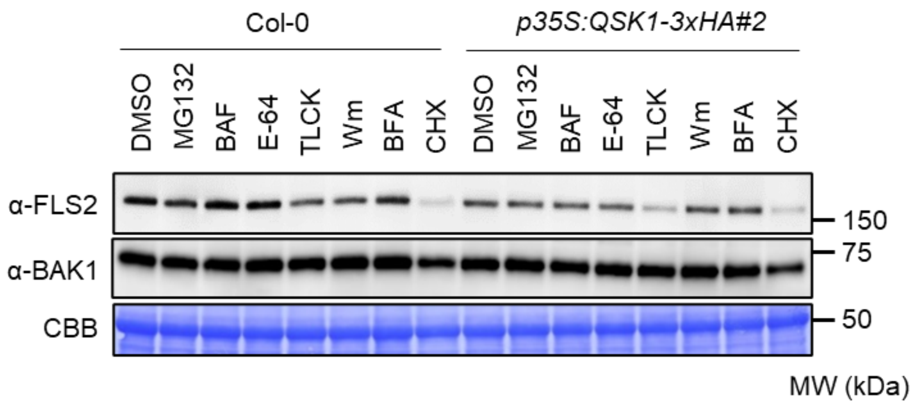

Supplementary Figure S13. Pharmacological analyses of QSK1-induced FLS2 reduction. (Supports Figure 5)
Two-week-old seedlings of Col-0 and p35S:QSK1-3×HA#2 line were treated with DMSO, 100 μM MG132, 10 μM Bafilomycin A1 (BAF), 33 μM Wortmannin (Wm) and 100 μg/mL Brefeldin A (BFA), 20 μM E-64d, 1 mM N-α-Tosyl-L-Lysinyl-chloromethyl ketone (TLCK) and 100 μM Cycloheximide (CHX) for 3 h and FLS2 and BAK1 protein levels were analyzed by immunoblotting with α-FLS2 and α-BAK1 antibodies. Equal loading of protein samples is shown by CBB staining. The experiments were repeated three times with similar results.

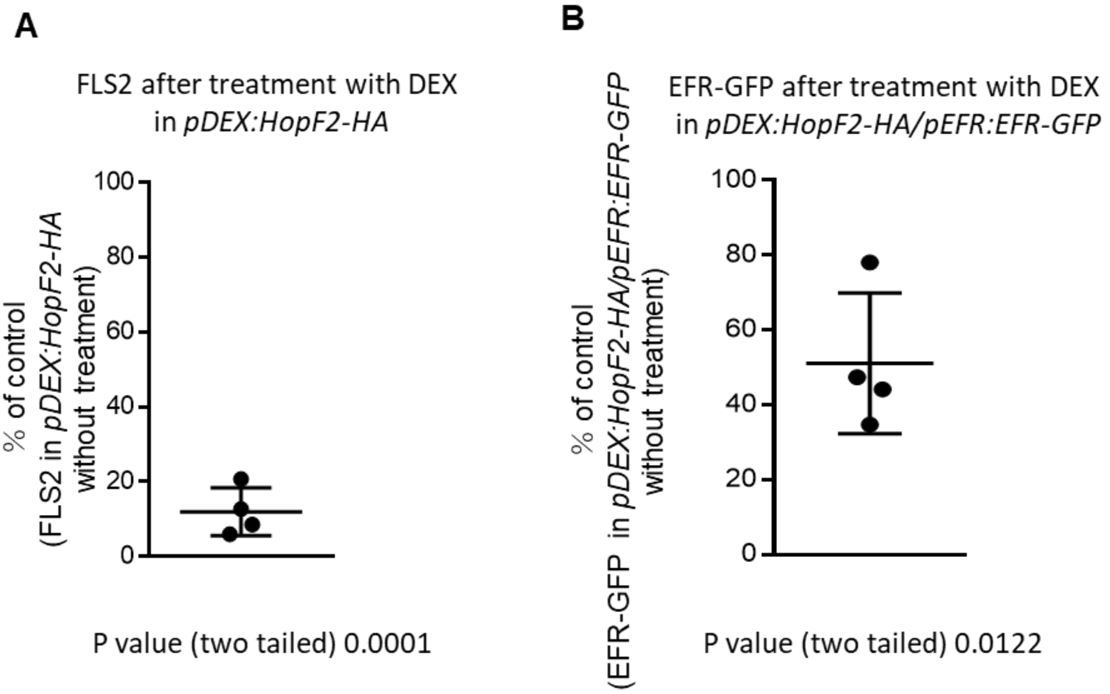

Supplementary Figure S14. Quantification of PRR levels in pDEX:HopF2-HA . (Supports Figure 6)

A) FLS2 protein level in pDEX:HopF2-HA at 24 h after DEX treatment compared to that without treatment. **B**) EFR-GFP protein level in pDEX:HopF2-HA /pEFR:EFR-GFP at 24 h after DEX treatment compared to that without treatment. Two-week-old Arabidopsis seedlings of pDEX:HopF2Pto-HA or pDEX:HopF2Pto-HA/pEFR:EFR-GFP were treated with or without 30 μM DEX, and FLS2 and EFR-GFP protein levels were measured by immunoblotting with α-FLS2 and α-GFP antibodies, respectively. The signals of the bands were quantified with ImageJ software. Values are mean (center line) ±SD of relative FLS2 or EFR-GFP protein levels compared to the control (FLS2 in pDEX:HopF2Pto-HA without DEX treatment, or EFR-GFP in *pDEX:HopF2*$_{Pto}$*-HA/pEFR:EFR-GFP* without DEX treatment) from four independent experimens. Individual data points are shown as dots. Statistical analysis was performed using a two-tailed one-sample t-test.

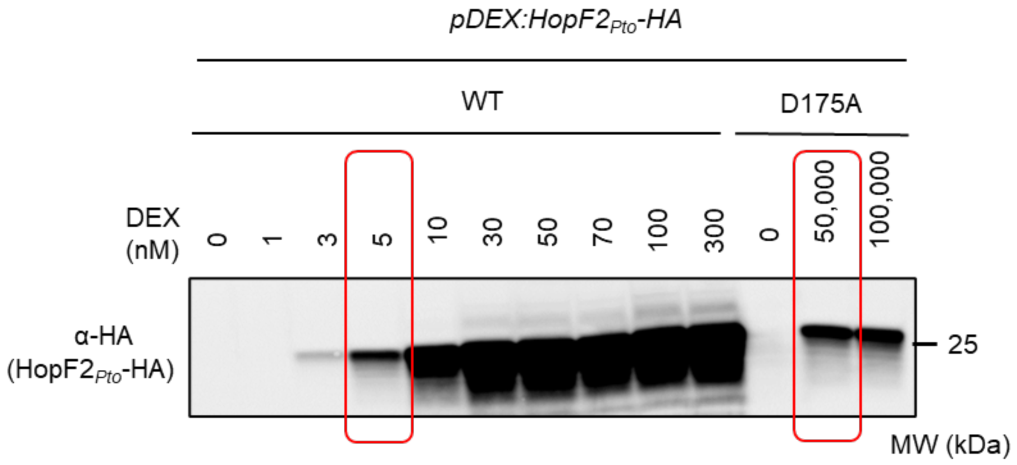

Supplementary Figure S15. The DEX concentration dependency for the expression of *pDEX:HopF2$_{Pto}$-HA* (WT) and *pDEX:HopF2$_{Pto}$-HA* (D175A). (Supports Figure 7)

Two-week-old Arabidopsis seedlings of *pDEX:HopF2$_{Pto}$-HA* (WT) and *pDEX:HopF2$_{Pto}$-HA* (D175A) were treated with or without different concentration of DEX and HopF2$_{Pto}$-HA protein levels were measured by immunoblotting with α-HA antibody. The protein levels of HopF2$_{Pto}$-HA in *pDEX:HopF2$_{Pto}$-HA* (WT) after treatment with 5 nM DEX and in *pDEX:HopF2$_{Pto}$-HA* (D175A) after treatment with 50 μM DEX are similar as highlighted by red boxes.

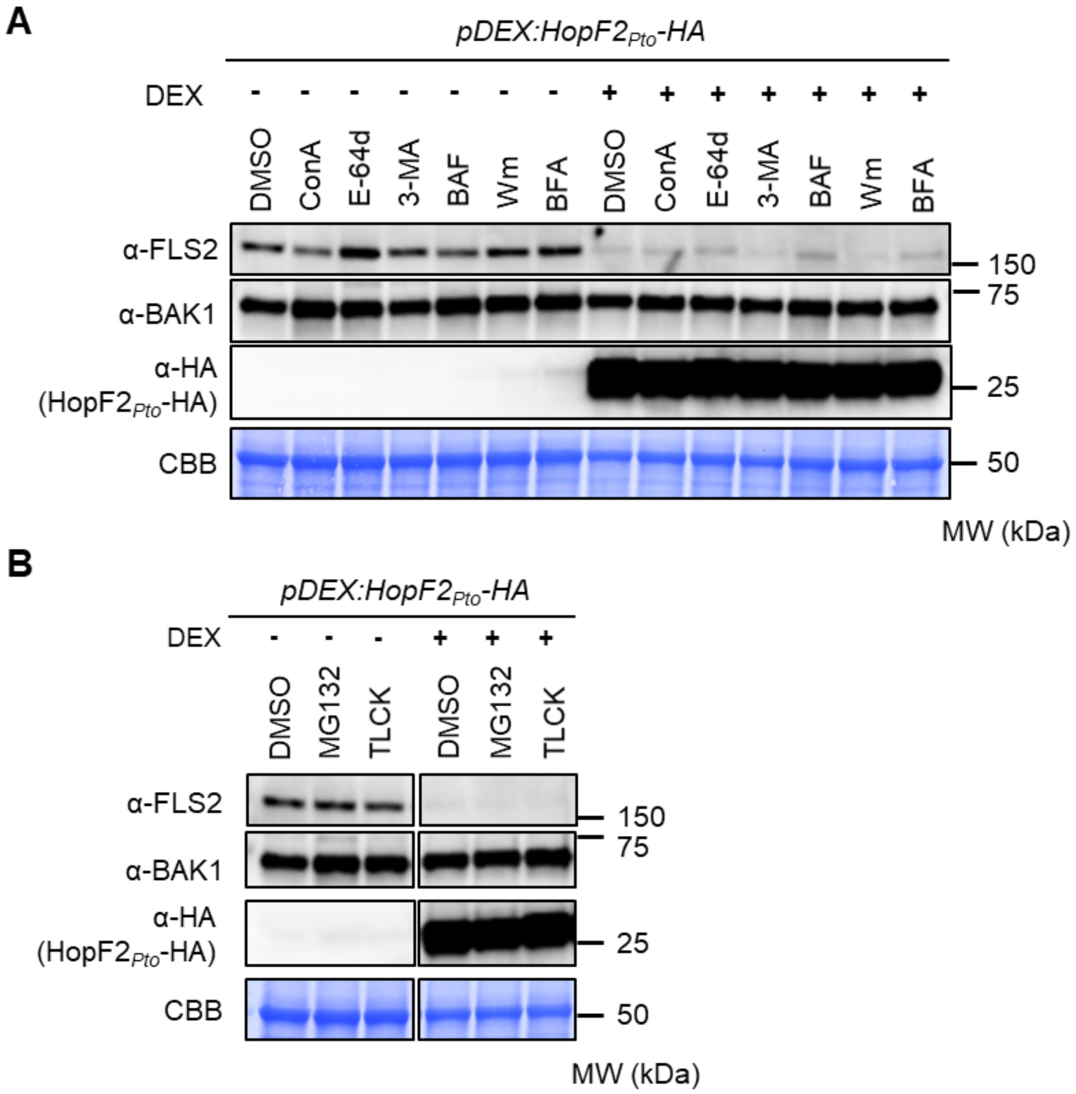

Supplementary Figure S16. Pharmacological analyses of HopF2$_{Pto}$-induced FLS2 reduction. (Supports Figure 7)

A-B) Two-week-old seedlings of *pDEX:HopF2$_{Pto}$-HA* line was treated with (+) or without (-) 30 μM DEX for 24 h, followed by the treatment with 1 μM ConA, 20 μM E-64d, 20 nM 3-MA, 10 μM Bafilomycin A1 (BAF), 33 μM Wortmannin (Wm), 100 μg/mL Brefeldin A (BFA) (shown in **A**), 100 μM MG132, and 1 mM N-α-Tosyl-L-Lysinyl-chloromethyl ketone (TLCK) (shown in **B**) for 3 h. FLS2, BAK1, and HopF2$_{Pto}$-HA protein levels were analyzed by immunoblotting with α-FLS2, α-BAK1, α-HA antibodies. Equal loading of protein samples is shown by CBB staining. The experiments were repeated three times with similar results.

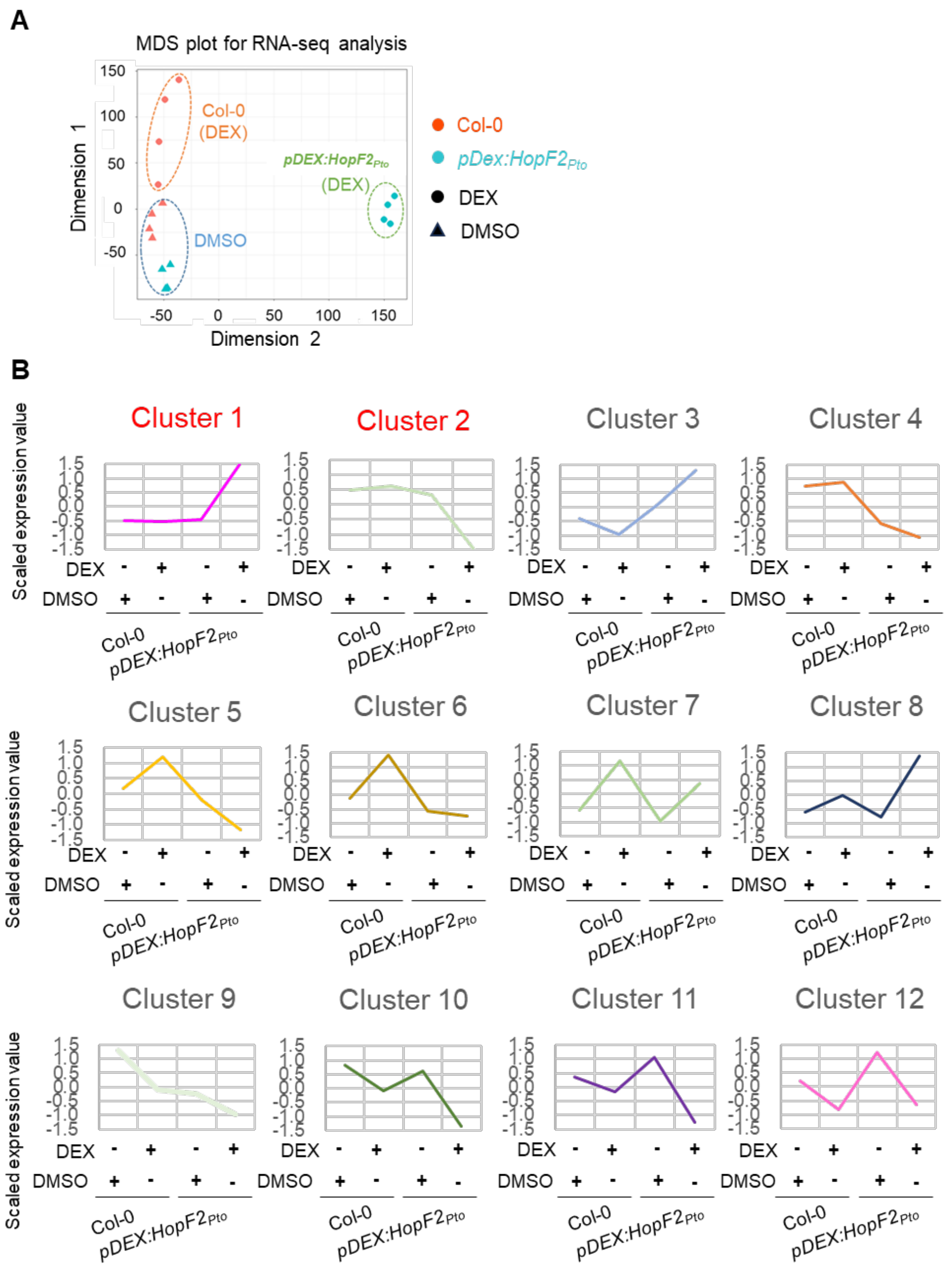

Supplementary Figure S17. Multidimensional scaling (MDS) plot with self-organizing map (SOM) clustering of genes affected by HopF2*Pto*. (Supports Figure 8)

A) MDS plot of global gene expression of Col-0 and *pDEX:HopF2Pto-HA* after treatment with (-) or without (+) DMSO or 30 µM DEX. Two-week-old seedlings of Col-0 and *pDEX:HopF2Pto-HA* were treated with DMSO or 30 µM DEX for 24 h. The transcript levels were analyzed by RNA-seq. **B)** Scaled expression profiles of genes in each of the twelve clusters. The scaled expression values represent the average principal component scores of all genes within a cluster. Genes in clusters 1 and 2 were specifically induced and reduced by HopF2*Pto*, respectively. The list of genes in each cluster is shown in Supplementary Data Set S4.

The relative expression values

0 403.5

Accession	Col-0 + DMSO	Col-0 + DEX	*pDEX:HopF2$_{Pto}$* + DMSO	*pDEX:HopF2$_{Pto}$* + DEX	FDR
IOS1	205.5	342.9	100.0	7.2	2.03E-51
QSK1	121.2	131.6	100.0	135.6	1.82E-03
SYP71	106.9	114.6	100.0	126.1	1.43E-02
HIR1	114.2	140.1	100.0	159.1	1.66E-07
HIR4	105.1	157.5	100.0	403.5	1.39E-57
GSL10	94.2	106.6	100.0	125.6	1.80E-02
PLD DELTA	96.7	101.0	100.0	134.7	1.22E-03
RBOHD	39.2	70.2	100.0	129.4	2.20E-21
Remorin	1394.7	922.4	100.0	1364.6	n.s
AHA11	111.2	85.0	100.0	112.8	n.s
BAK1	217.8	264.2	100.0	247.5	n.s
PIP2E	2008.5	1774.7	100.0	813.6	n.s
ACA10	104.0	135.9	100.0	134.9	n.s

Supplementary Figure S18. HopF2$_{Pto}$ affects some transcript levels of commonly associated proteins with EFR, FLS2, and RBOHD. (Supports Figure 8)
Transcript levels of the commonly associated proteins with EFR, FLS2, and RBOHD were measured by RNA-seq in two-week-old seedlings of Col-0 and *pDEX:HopF2$_{Po}$-HA* after treatment with 30 μM DEX for 24 h. The relative expression values of the genes are shown compared to the "*pDEX:HopF2$_{Pto}$* + DMSO" control. The FDR values between "*pDEX:HopF2$_{Pto}$* + DMSO" and "*pDEX:HopF2$_{Pto}$* + DEX" are shown. Heatmaps indicate relative gene expression values. Grey boxes in the heat map indicate no statistically significant difference at FDR ≤ 0.05.

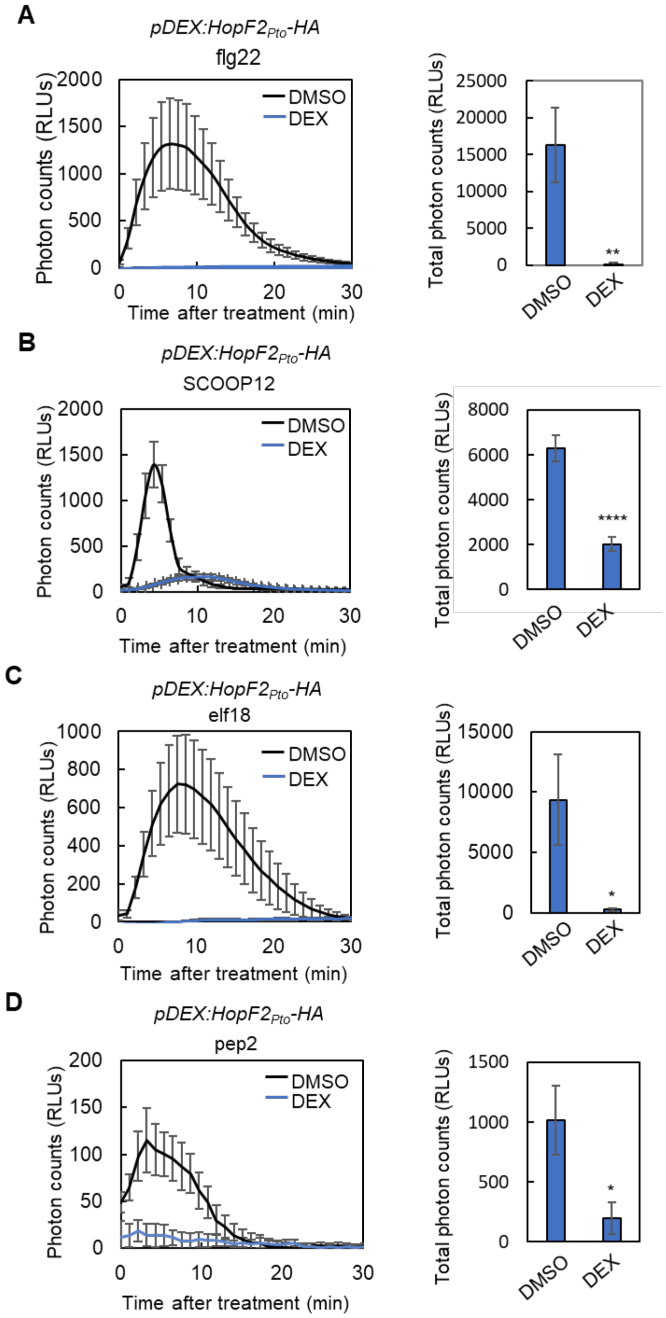

Supplementary Figure S19. HopF2$_{Pto}$ inhibits PAMP-induced ROS production. (Supports Figure 9)

Ten-day-old Arabidopsis seedlings of *pDEX:HopF2$_{Pto}$-HA* were treated with DMSO or 30 μM DEX for 24 h and the time-course of ROS production was monitored upon treatment with 1 μM flg22 (**A**), 1 μM SCOOP12 (**B**), 1 μM elf18 (**C**), and 1 μM pep2 (**D**). Eight seedlings were used for each treatment. and the time-course (left) and total amount (right) of ROS production were measured by a luminol-based assay. Values are mean ± SE from 8 seedlings. Asterisks indicate a significant difference based on Student's t-test ($^{*}p \le 0.05$, $^{**}p \le 0.01$, $^{****}p \le 0.001$). The experiments were repeated three times with similar results.

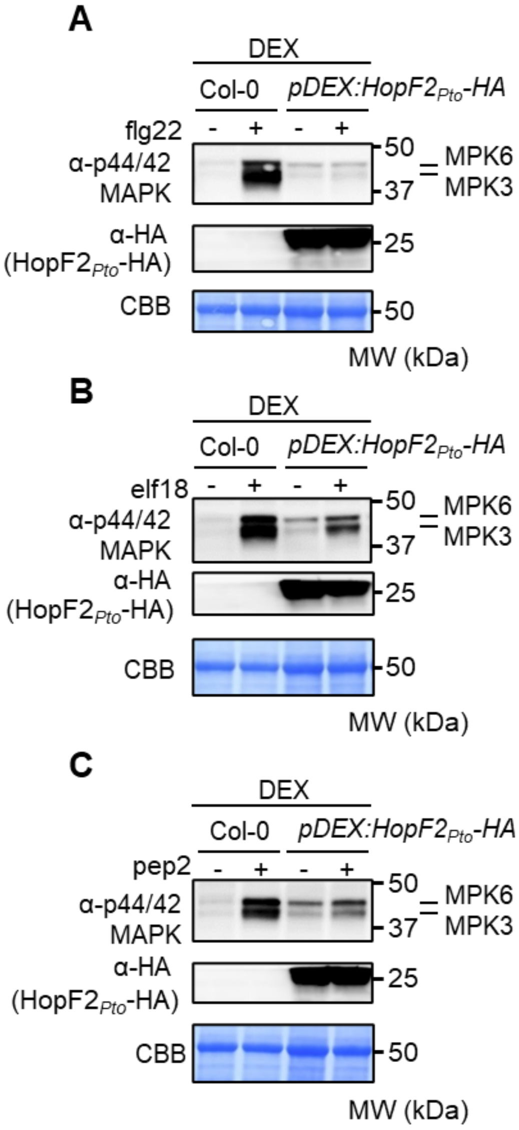

Supplementary Figure S20. HopF2*Pto* inhibits PAMP-induced MAPK activation. (Supports Figure 9)

Ten-day-old Arabidopsis seedlings of Col-0 and *pDEX:HopF2Pto-HA* were treated with 30 μM DEX for 24 h and then treated with (+) or without (-) 1 μM flg22 (**A**), 1 μM elf18 (**B**), or 1 μM pep2 (**C**) for 15 min. Phosphorylated MAPKs were detected by immunoblotting with α-phospho-p44/42 MAPK (Erk1/2) (Thr202/Tyr204) antibody. Equal loading of protein samples is shown by CBB staining. These experiments were repeated three times with similar results.

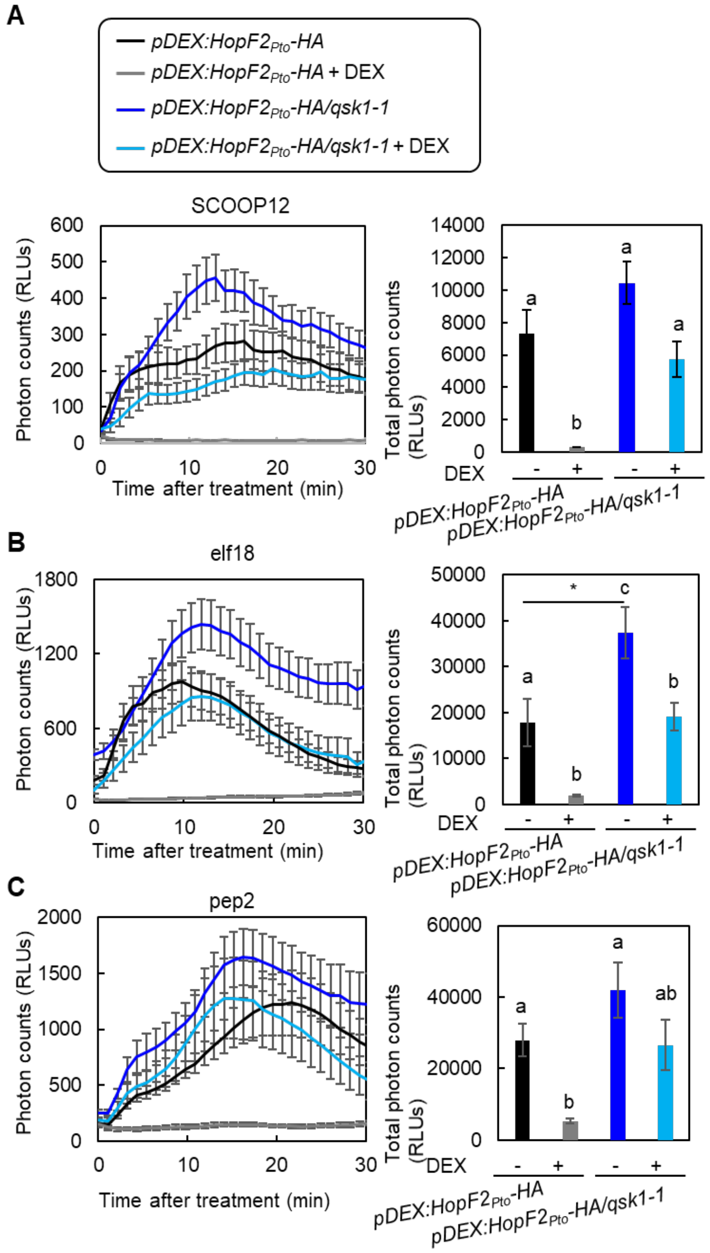

Supplementary Figure S21. HopF2*Pto* requires QSK1 to inhibit PAMP-inducible ROS production. (Supports Figure 10)

Eight seven-day-old Arabidopsis seedlings of *pDEX:HopF2Pto-HA* and *pDEX:HopF2Pto-HA*/*qsk1-1* were treated with 30 μM DEX for 24 h, followed by the treated with (+) or without (-) 1 μM SCOOP12 (**A**), 1 μM elf18 (**B**), or 1 μM pep2 (**C**). The time-course (left) and the total amount (right) of flg22-inducible ROS production were measured by a luminol-based assay. Values are mean ± SE from 16 leaf discs. Data for *pDEX:HopF2Pto-HA* and *pDEX:HopF2Pto-HA /qsk1-1* without treatment are shown in black and blue, respectively, while data with DEX treatment are represented in gray and light blue, respectively for clarity. Different letters indicate significant differences based on one-way ANOVA and Tukey's post hoc test ($p \le 0.05$). Asterisks indicate a significant difference between indicated samples based on Student's t-test ($^{*}p \le 0.05$). These experiments were repeated three times with similar results.

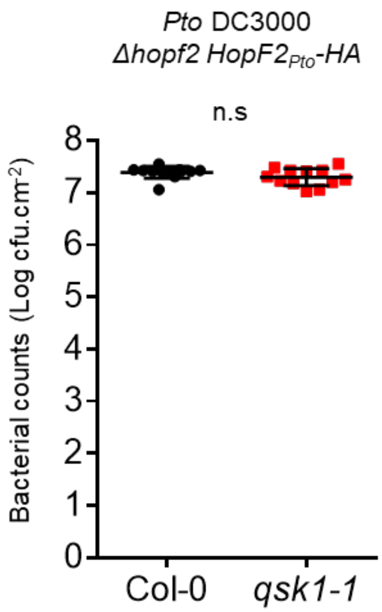

Supplementary Figure S22. The population of *Pto* DC3000 *Δhopf2 HopF2$_{Pto}$-HA* remains consistent between Col-0 and *qsk1-1* mutant after 24 h syringe infiltration. (Supports Figure 10)

The experimental condition is the same as that in Fig.**10C**. Five-week-old Arabidopsis Col-0 and *qsk1-1* mutant were syringe-inoculated with *Pto* DC3000 *Δhopf2 HopF2$_{Pto}$-HA* (inoculum: 10^8 cfu/ml). Values are means ± standard deviation from 12 plants. The central horizontal line indicates the mean value. There is no statistically significant difference at $p \leq 0.05$ (Student's t-test). This experiment was repeated three times with similar results. The data for Col-0 are shown as black circles and the data for *qsk1-1* are shown as red squares for clarity.

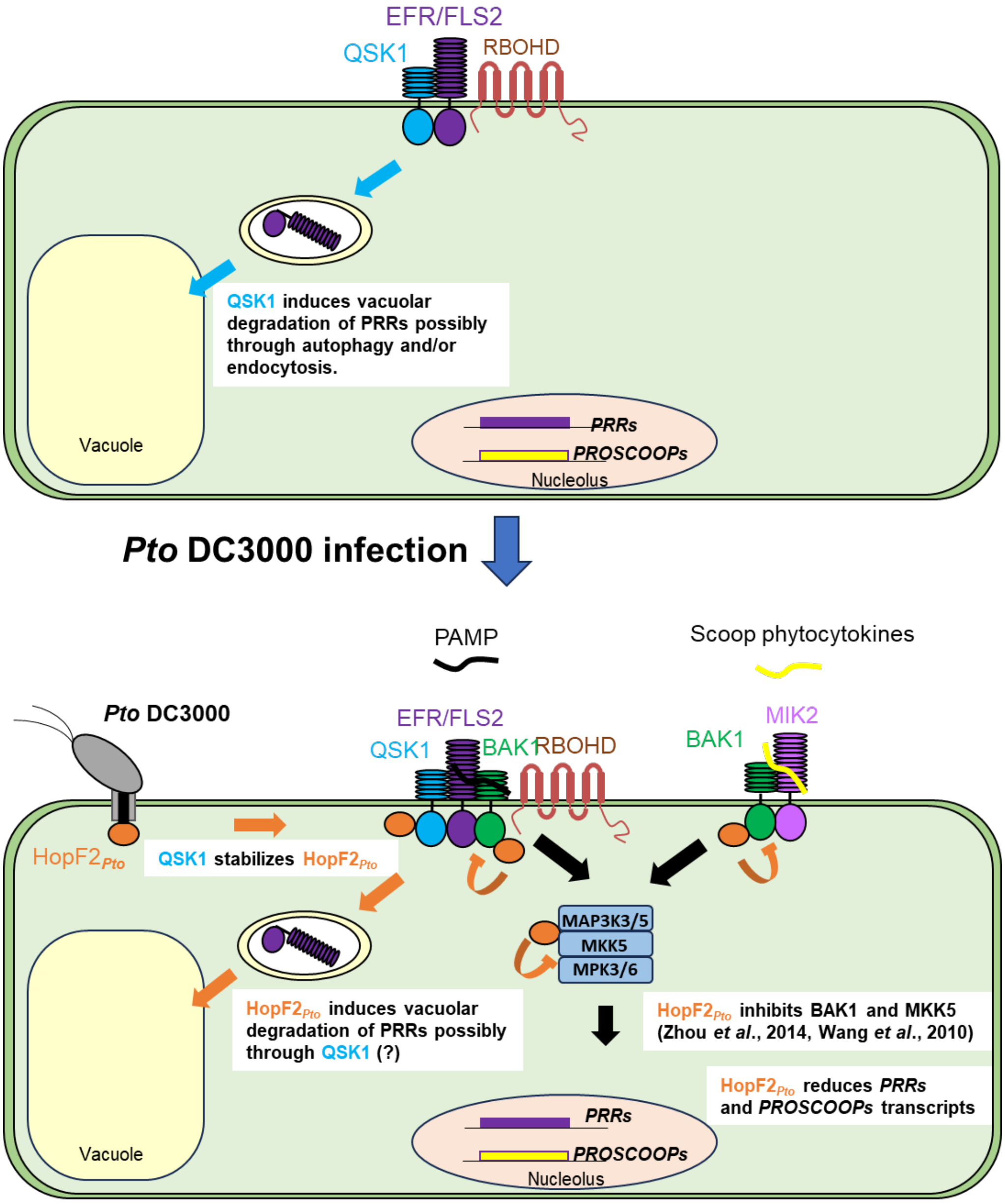

Supplementary Figure S23. A model of HopF2$_{Pto}$ virulence function in suppressing PTI and its relationship with QSK1.

Upon injection into plant cells through the Type III secretion system, HopF2$_{Pto}$ initiates interactions with QSK1, leading to the stabilization of HopF2$_{Pto}$. This interaction might also facilitate HopF2$_{Pto}$'s targeting of the FLS2 and EFR complexes at the plasma membrane. Consequently, HopF2$_{Pto}$ reduces PRR protein levels possibly through autophagy and/or endocytosis-dependent protein degradation. Additionally, HopF2$_{Pto}$ reduces the expression of defense-related genes, such as *PRRs* and *PROSCOOPs*. HopF2$_{Pto}$ was also shown to target BAK1 and MKK5, which might be involved in HopF2$_{Pto}$-mediated transcriptome reprogramming of plant cells (Wang *et al.*, 2010; Zhou *et al.*, 2014). These sophisticated strategies deployed by HopF2$_{Pto}$ effectively shut down the recognition of PAMPs, DAMPs, and SCOOP phytocytokines during pathogen attacks.

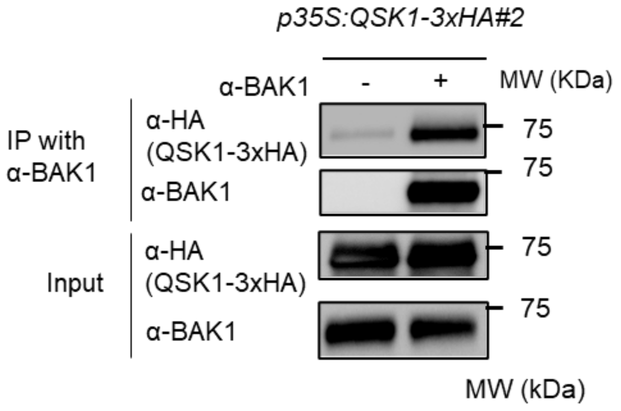

Supplementary Figure S24. QSK1 associates with BAK1 *in vivo*.
Total protein (input) from two-week-old Arabidopsis seedlings of *p35S:QSK1-3xHA#2* was immunoprecipitated with (+) α-BAK1 antibody, followed by immunoblotting with α-BAK1 and α-HA antibodies. For the negative control, the same immunoprecipitation procedure was performed without (-) α-BAK1 antibody. The experiments were repeated three times with similar results.

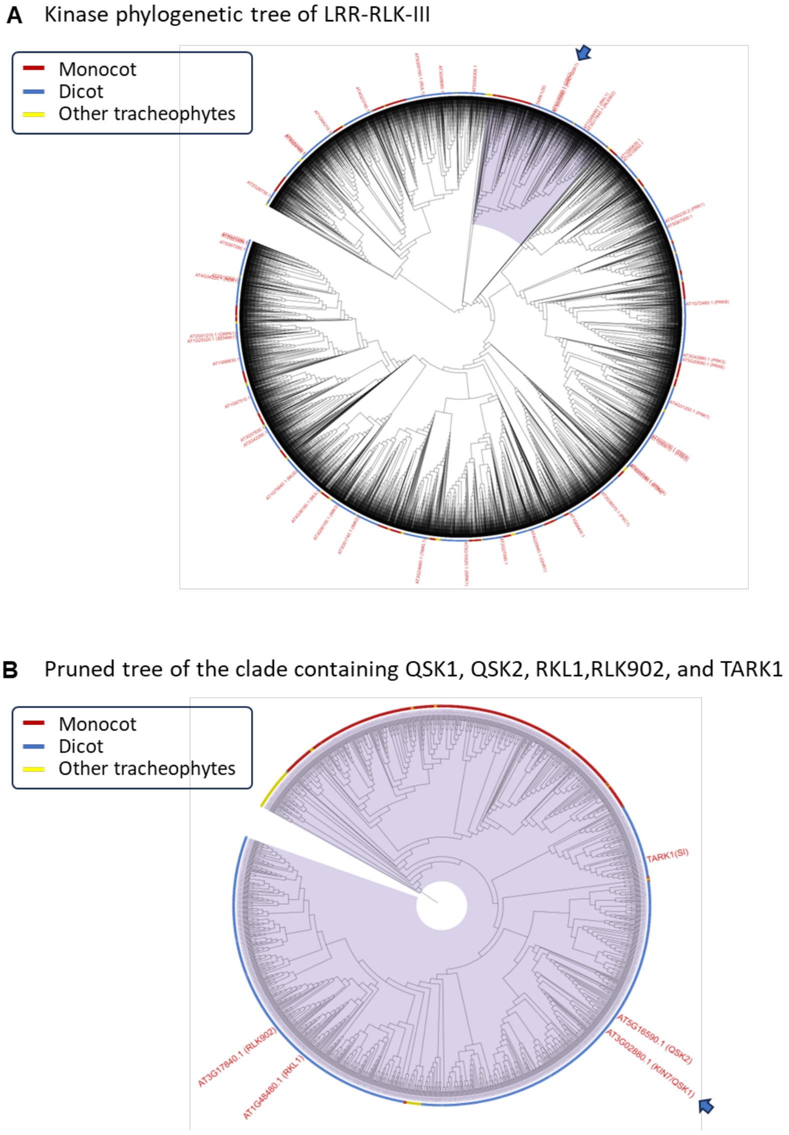

Supplementary Figure S25. QSK1 homologs are widely conserved across most tracheophytes.

A) Kinase phylogenetic tree of LRR-RLK-III identified in annotated proteomes from 350 publicly available genomes (14,080 members) (Ngou *et al.*, 2022). The outer ring represents the classification of species (red indicates monocots, blue indicates dicots, yellow indicates other tracheophytes including lycophytes, polypodiophyta, and gymnosperms). Arabidopsis members and tomato TARK1 are labeled in red characters. Arabidopsis QSK1 is shown by blue arrows. **B**) The pruned tree corresponds to the purple area in the upper tree (**A**). TARK1, AT5G16590 (QSK2), AT3G02880 (QSK1), AT1G48480 (RKL1), and AT3G17840 (RLK902) cluster within the purple clade, with monocot, dicot, other tracheophyte orthologs (1,306 members). Arabidopsis QSK1 is shown by blue arrows. Phylogenetics trees and annotation files from both (A) and (B) are provided as supplementary files (Supplementary Data Set S7-10). Tree branch lengths, bootstrap values, and other relevant information can be obtained from the tree files.

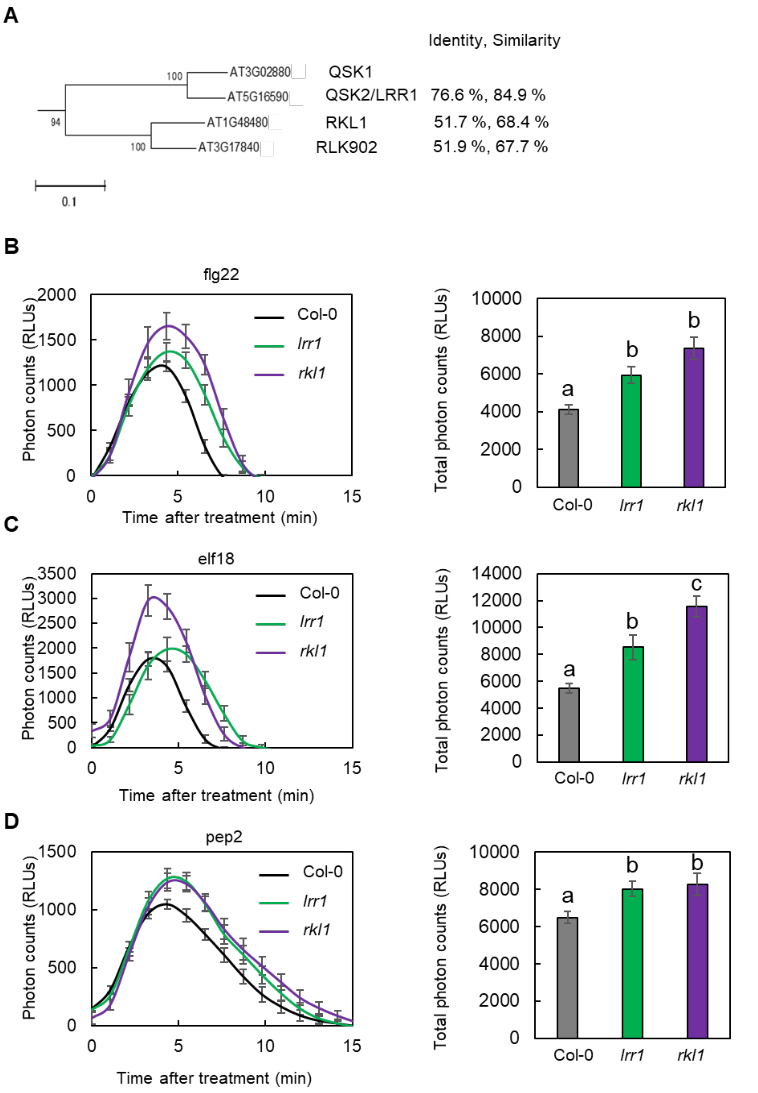

Supplementary Figure S26. *lrr1* and *rkl1* mutants have higher ROS production in response to flg22, elf18, and pep2.

A) Phylogenetic tree of full-length proteins of QSK1, LRR1, RKL1 and RLK902. QSK1 belongs to the LRR-RLK III subgroup and there are three similar proteins to QSK1: LRR1 (AT4G16590) with 83.8% similarity and 75.4% identity to QSK1, RKL1 (AT5G16590) with 81.0% similarity and 50.0% identity, and RLK902 (AT3G17840) with 80.0% similarity and 51.0% identity. The length of the branches indicates the phylogenic differences between the proteins and are in the units of the number of amino acid substitutions per site. The percentage of replicate trees in which the associated taxa clustered together in the bootstrap test (10000 replicates) are shown next to the branches. **B, C, and D)** *lrr1* and *rkl1* mutants induce higher ROS production than Col-0 upon treatment with 1 μM flg22 (**B**), 1 μM elf18 (**C**), or 1 μM pep2 (**D**). Eight leaf discs from four- to five-week-old Arabidopsis plants were used for ROS assays. The time-course (left) and total amount (right) of ROS production were measured by a luminol-based assay. Values are mean ± SE from 8 leaf discs. Different letters indicate significant differences based on one-way ANOVA and Tukey's post hoc test ($p \leq 0.05$). The data for *lrr1* and *rkl1* are shown in green and purple, respectively, for clarity. The experiments were repeated three times with similar results.

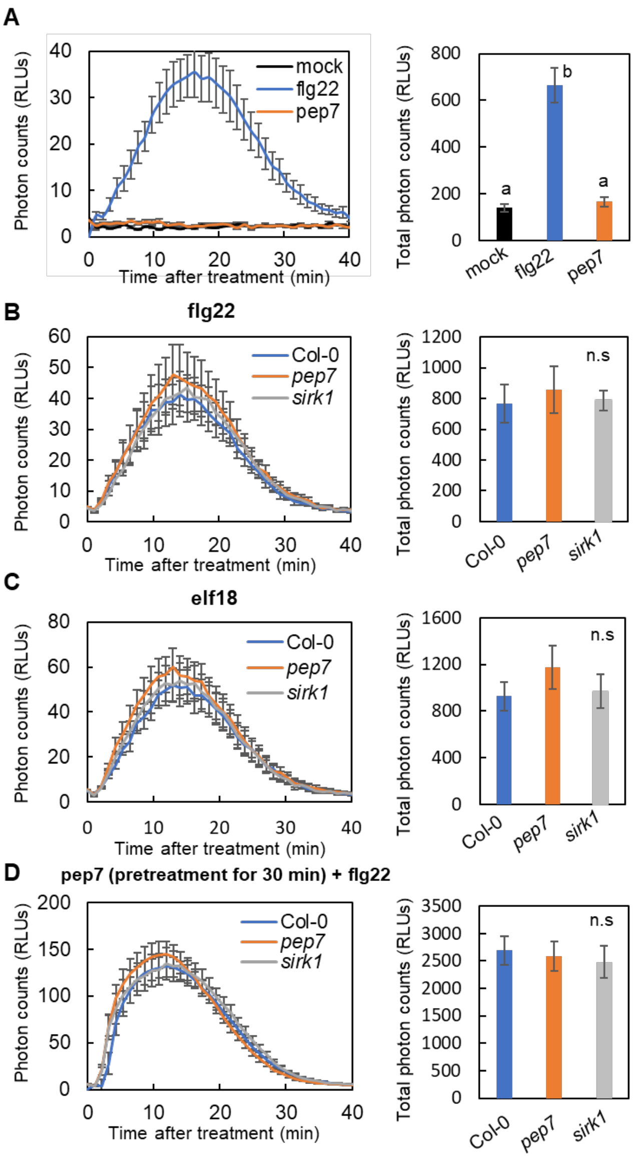

Supplementary Figure S27. SIRK1 and PEP7 do not affect PAMP-induced ROS production.

A) The treatment with 1 μM pep7 does not induce ROS production in Col-0 plants. The data of flg22 and pep7 are shown in light blue and orange, respectively for clarity. **B and C)** *pep7* and *sirk1* mutants induce similar ROS production to Col-0 in response to 1 μM flg22 (**B**) and 1 μM elf18 (**C**). **D)** Pretreatment with 1 μM pep7 peptide for 30 min does not affect ROS production induced by 1 μM flg22. Eight leaf discs from four five-week-old Arabidopsis soil-grown plants were used for ROS assays. The time-course (left) and total amount (right) of ROS production were measured by a luminol-based assay. Values are mean ± SE from 8 leaf discs for **A-C**, and 16 leaf discs for **D**. Different letters in **A** indicate significant differences based on one-way ANOVA and Tukey's post hoc test ($p \leq 0.05$). There is no statistically significant difference (n.s) at $p \leq 0.05$ (ANOVA and Tukey's post hoc test) for **B-D**. The data of *pep7* and *sirk1* are shown in orange and gray, respectively for clarity. The experiments were repeated three times with similar results.

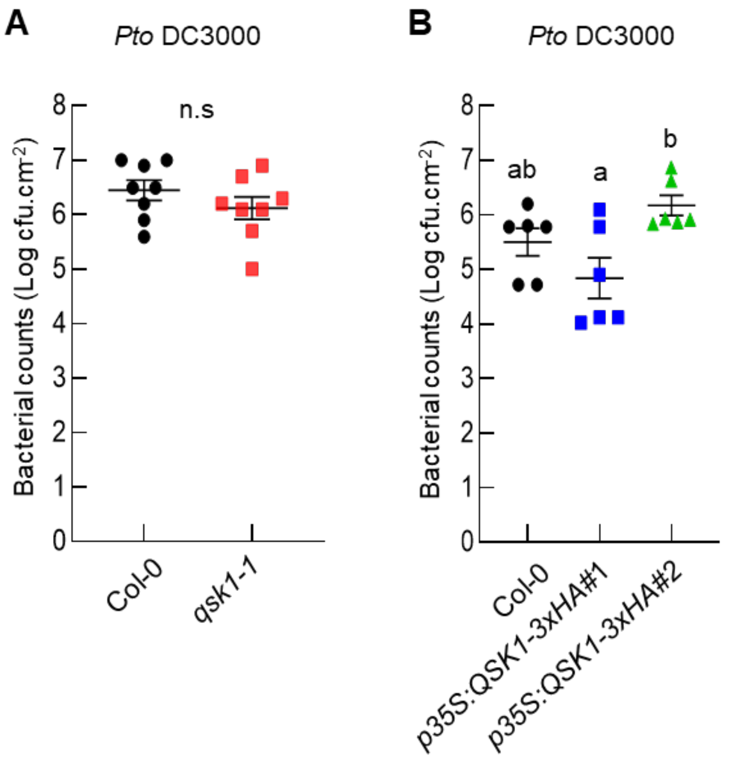

Supplementary Figure S28. Bacterial growth in *qsk1-1* and *p35S:QSK1-3xHA* lines.

(Supports Figure 3 and Figure 4)

A) The growth of *Pseudomonas syringae* pv. *tomato* (*Pto*) DC3000 in *qsk1-1* was similar to that in Col-0. Values are means ± standard deviation from 8 plants. There is no significant difference (n.s) at $p \leq 0.05$ (Student's t-test). The data of *qsk1-1* is shown in red for clarity. **B**) The growth of *Pto* DC3000 in *p35S:QSK1-3xHA* lines was similar to that in Col-0. Values are means ± standard deviation from 6 plants. Different letters indicate significant differences at $p \leq 0.05$ (one-way ANOVA, Tukey's post hoc test). *Pto* DC3000 was sprayed onto leaf surfaces of six-week-old soil-grown Arabidopsis plants at a concentration of $1x10^5$ cfu /mL. Three days post-spray-inoculation, leaves were harvested to determine bacterial growth. The data for *p35S:QSK1-3xHA#1* and *#2* are shown as light blue squares and green triangles, respectively, for clarity. All the experiments were repeated three times with similar results.

Supplementary Methods S1 Additional methods

Chemical inhibitors

Ten- to fourteen-day-old Arabidopsis seedlings grown in MS liquid media were treated with DMSO, 100 µM MG132, 10 µM Bafilomycin A1 (BAF, Funakoshi, Tokyo, Japan), 33 µM Wortmannin (Wm, FUJIFILM Wako Pure Chemical Corporation, Osaka, Japan), 100 µg/mL Brefeldin A (BFA, Sigma-Aldrich, St. Louis, MO, USA), 20 µM E-64d (Sigma-Aldrich), 1 mM N-α-Tosyl-L-Lysinyl-chloromethyl ketone (TLCK, FUJIFILM Wako), 100 µM Cycloheximide (CHX, FUJIFILM Wako), 1 µM Concanamycin A (ConA, Sigma-Aldrich), and 20 nM 3-methyladenine (3-MA Sigma-Aldrich) for 3 h.

Protein extraction and IP

Large-scale IP of EFR-GFP from *efr-1/pEFR:EFR-GFP* and 3xFLAG-RBOHD from *rbohD/pRBOHD:3xFLAG-RBOHD* were described previously (Kadota *et al.*, 2014; Goto *et al.*, 2023). IP of FLS2-GFP from *pFLS2:FLS2-GFP* was performed using the same protocol as that for EFR-GFP from *efr-1/pEFR:EFR-GFP*. α-GFP magnetic beads (Miltenyi Biotec, Bergisch-Gladbach, Germany) were used for capturing the proteins, and boiled SDS sample buffer was used for the elution. IP of RBOHD-3xFLAG from *rbohD/pRBOHD:3xFLAG-RBOHD* was performed using agarose beads of α-FLAG (ANTI-FLAG M2 affinity gel, Sigma-Aldrich), and 3xFLAP peptides were used for the elution. For small-scale co-IP with EFR-GFP, FLS2-GFP, QSK1-GFP, and 3xFLAG-RBOHD*,* we used two grams of Arabidopsis seedlings and followed the same protocol. α-GFP magnetic beads, α-FLAG magnetic beads (Miltenyi Biotec), and α-GFP agarose beads (ChromoTek, Bayern, Germany) were used for capturing the proteins, and boiled SDS sample buffer was used for the elution. For co-IP with BAK1 shown in Fig.5G, α-rabbit Trueblot agarose beads (eBioscience) coupled with α-BAK1 were used and anti-rabbit IgG-HRP Trueblot (Rockland, 18-8816-31) was used for the detection.

Protein identification by LC-MS/MS

The identification of proteins by LC-MS/MS was performed as previously described (Kadota *et al.*, 2014). In brief, proteins were separated by SDS-PAGE (Invitrogen NuPAGE precast gel, Thermo Fisher Scientific, Waltham, MA USA) and after staining with Coomassie Brilliant Blue (CBB) (Invitrogen SimplyBlue™ stain, Thermo Fisher Scientific), the proteins were excised from the gel and digested with trypsin. LC-MS/MS analysis was performed using an LTQ-Orbitrap mass spectrometer (Thermo Fisher Scientific) and a nanoflow-HPLC system (nanoAcquity; Waters, Milford, MA, USA). The entire TAIR10 database was searched (www.Arabidopsis.org) using Mascot algorithm (v 2.3.02, Matrix Science, Boston, MA, USA) (with the inclusion of sequences of common contaminants, such as keratins and trypsin). The Scaffold program (Proteome Software, Portland, OR, USA) was used to validate MS/MS-based peptide and protein identifications and to annotate spectra.

ROS burst assay

Eight or sixteen leaf discs (4 mm in diameter) were collected from four- to six-week-old Arabidopsis plants or five-week-old *N. benthamiana*. For ROS burst assay in seedlings, eight seven-day-old Arabidopsis seedlings were used. The leaf discs were then floated overnight on sterile water. The next day, the water was replaced with a solution containing 40 µM Luminol (FUJIFILM Wako, Tokyo, Japan), 20 µg/mL horseradish peroxidase (HRP) (Sigma-Aldrich, St. Louis, MO, USA), and 1 µM flg22 or 1 µM elf18 for the detection of flg22- or elf18-induced ROS production. For the ROS burst assay using *pDEX:HopF2*$_{pto}$*-HA* line, ten-day-old seedlings grown in 96 well plates and a solution

containing 1 µM L-012 (Wako, Japan) and 20 µg/mL HRP were used. Luminescence was measured using a Tristar2 multimode reader (Berthold Technologies, Bad Wildbad, Germany).

MAPK activation assay

MAPK activation assays were performed as described previously (Goto *et al.*, 2020). Ten-day-old Arabidopsis seedlings were flash-frozen with liquid nitrogen, and proteins were extracted in protein extraction buffer (50 mM Tris-HCl pH 7.5, 150 mM NaCl, 10% glycerol, 2 mM EDTA, 5 mM DTT, 1 × EDTA-free Complete Protease Inhibitor Cocktail [Roche, USA], 0.1% IGEPAL CA630, 0.5 mM PMSF, 1 mM Na_2MoO_4, 1 mM NaF, 0.5 mM Na_3VO_4, 20 mM β-glycerophosphate). The extract was then centrifuged at 16,000 × *g* to remove insoluble material, and the protein concentration of the supernatant was measured using the Bradford method (Bio-Rad Laboratories, USA). Total proteins were separated by SDS-PAGE and transferred onto a PVDF membrane according to the manufacturer's instructions (Transblot, Bio-Rad Laboratories, CA, USA). The membrane was blocked overnight at 4°C in a solution of 5% (w/v) skim milk (Wako, Japan) in Tris-buffered saline with 0.05% (v/v) Tween 20 (TBS-T). Phosphorylated MAPKs were detected using α-phospho-p44/42 MAPK (Erk1/2) (Thr202/Tyr204) (D13.14.4E) rabbit monoclonal antibody (1:2000, Cell Signaling Technology, USA) for 1 h at room temperature in a solution of 5% (w/v) BSA (Sigma-Aldrich, Japan) in TBS-T, followed by incubation with α-rabbit IgG-HRP-conjugated secondary antibodies (1:10000, Roche, Basel, Germany) for 1 h at room temperature in a solution of 5% skim milk in TBS-T. The HRP-conjugated antibody signal was detected using Super Signal West Femto Maximum Sensitivity Substrate (Thermo Fisher Scientific, MA, USA) with a LAS 4000 system (GE Healthcare, USA). The PVDF membranes were stained with Coomassie Brilliant Blue (CBB) to verify equal loading.

Bacterial infection assays

Bacterial infection assays were performed using *Pseudomonas syringae pv. tomato* (*Pto)* DC3000 COR-, *P. syringae pv. cilantro* (*Pci*) 0788-9, and *Pto* DC3000 *ΔhopF2 HopF2pto-HA* as described previously (Zipfel *et al.*, 2004; Goto *et al.*, 2020). The bacterial strains were grown overnight in LB medium supplemented with 500 µg/mL kanamycin and 100 µg/mL rifampicin. The bacteria were harvested by centrifugation, and pellets were re-suspended in 10 mM $MgCl_2$ to 1.0×10^7 CFU/mL. Immediately before spraying, 0.02% (v/v) Silwet L-77 was added to bacterial suspensions, and bacteria were sprayed onto leaf surfaces of five- to six-week-old plants. Leaf discs were collected three days after inoculation from three leaves per plant and six plants per genotype. The leaf discs were ground in 10 mM $MgCl_2$, diluted, and plated on LB agar with appropriate selection. The plates were incubated at 28°C and the number of colonies was counted two days later. In the bacterial infection assays shown in Fig. 10E and Fig.S22, five-week-old Arabidopsis Col-0 and *qsk1-1* mutant were syringe inoculated with *Pto* DC3000 *Δhopf2 HopF2*$_{Pto}$*-HA* (inoculum: 10^8 cfu/mL). Leaf discs were collected 24 h after inoculation for colony counting.

Phylogenetic analyses

The phylogenetic trees in Fig. S25 were drawn using the data published previously and visualised and pruned, and figures were generated with iTOL (Ngou *et al.*, 2022; Ngou *et al.*, 2024). For alignment and phylogeny methods, refer to https://github.com/MWSchmid/Ngou-et-al.-2022. Alignment files and tree files were taken from https://doi.org/10.5281/zenodo.7017981. The phylogenetic tree in Fig. S26 was drawn using the Neighbor-joining method (Saitou & Nei, 1987) based on amino acid

sequences of full-length proteins. The percentage of replicate trees in which the associated taxa clustered together in the bootstrap test (10,000 replicates) are shown next to the branches (Felsenstein, 1985). The tree is drawn to scale, with branch lengths in the same units as those of the distances used to infer the phylogenetic tree. The distances were computed using the Poisson correction method and are in the units of the number of amino acid substitutions per site. The phylogenetic analyses were performed in MEGA11version 11.0.13 (Tamura *et al.*, 2021).

Transient expression in *N. benthamiana*

Agrobacterium tumefaciens AGL1 strains carrying the binary expression vectors (epiGreenB5-*p35S:QSK1-3×HA*, epiGreenB5-*p35S:QSK1-GFP* and pCAMBIA2300-pFLS2:gFLS2-GFP) were grown in LB medium supplemented with the appropriate antibiotics. The cultures were then centrifuged to collect the cell pellets, which were subsequently re-suspended in a buffer containing 10 mM $MgCl_2$, 10 mM MES pH 5.6, and 100 µM acetosyringone and adjusted the concentration at OD_{600}=0.6 and incubated for 3 h at room temperature before infiltration. *Agrobacterium* strains were syringe-infiltrated into the leaves of *N. benthamiana*.

Confocal microscopy analyses

Four-week-old *N. benthamiana* leaves were used to observe the subcellular localization of FLS2-GFP, and QSK1-GFP. The fluorescence signals of GFP were recorded using confocal laser scanning microscopy (Leica TCS SP5, Leica Microsystems GmbH, Wetzlar, Germany) after excitation at 488 nm for GFP with an argon laser. Fluorescence emission was collected between 500-540 nm for GFP. The Photograph was processed using LAS X version; 3.3.0.16799 and Fiji software (Schindelin *et al.*, 2012)

RT-qPCR assay

RT-qPCR was performed as described previously (Kadota *et al.*, 2019). Total RNA was extracted from Arabidopsis seedlings using an RNeasy Plant Mini Kit (Qiagen, Hilden, Germany) according to the manufacturer's instructions. RNA was reverse transcribed with a ReverTraAce qPCR RT Kit (Toyobo) according to the manufacturer's instructions. One µg of total RNA was used as a template for cDNA synthesis. RT-qPCR was carried out using Thunderbird SYBR qPCR Mix (Toyobo) with a Stratagene mx 3000p real-time thermal cycler (Agilent, CA, USA). Relative transcript levels were calculated against a standard curve with normalization to the expression of *PLANT U-BOX PROTEIN1* (*PUB1*) gene (*AT5G15400,* Azevedo *et al*., 2001). Primers used for the RT-qPCR are listed in Table S1.

QIS-Seq to identify interactors of HopF2*Pto*

HopF2Pto was amplified with *Pfu* polymerase (Fermentas, Waltham, MA, USA) with an in-frame HA epitope, a polybasic region (K6), and a CAAX box, flanked by *Sfi*I restriction sites. The polybasic region and CAAX box were used to tether bait proteins to the membrane and minimize autoactivation (Hancock *et al.*, 1990; Lewis *et al.*, 2012). *HopF2Pto* was cloned into the pBT3-N vector (Dualsystems Biotech, Zurich, Switzerland) under the weak CYC1 promoter using *Sfi*I, and the orientation of the gene was validated. QIS-Seq was performed as previously described (Lewis *et al.*, 2012; Gong *et al.*, 2017).

RNA-Seq and differential gene expression analyses

Ten-day-old seedlings of Col-0 and *pDEX:HopF2Pto-HA* line grown in liquid culture were treated with DMSO or 30 µM DEX for 24 h with four biological replicates. Transcript levels

were then analyzed by RNA-Seq. RNA-Seq library preparation was carried out using BradSeq protocol (Townsley *et al.*, 2015). The 50-bp single-end reads were sequenced on an Illumina NextSeq 500 platform. The FASTX toolkit 0.0.13.2 (Hannonlab) was used for quality filtering. The first 6 bp were trimmed from the reads to remove barcode sequences. Low-quality nucleotides (quality scores < 30) were removed from the 3′ ends and short reads (< 39 bp) and reads showing at least 95% of nucleotides with quality scores < 20 were removed. Filtered reads were mapped to the Arabidopsis genome TAIR10.30 by tophat v2.1.0 (Center for Computational Biology at Johns Hopkins University) (Trapnell *et al.*, 2009). Read counts were extracted by HTSeq, version 0.6.0 (Anders *et al.*, 2015). The downstream bioinformatic analysis was performed as described previously (Ichihashi *et al.*, 2018). The trimmed mean of M values (TMM) normalized reads (Table S5_1), derived using the Bioconductor package edgeR 3.22.3 (Robinson *et al.*, 2010), were used to create MDS plots and perform the differentially expressed gene analyses. MDS plots were made using the R stats package cmdscale. The differentially expressed gene data (FDR ≤ 0.05) were extracted using edgeR. Sequencing reads were deposited in DNA Data Bank of Japan (DDBJ) database under accession number DRA017478 (BioProject:PRJDB16845 (PSUB021573)).

PCA with SOM clustering and GO term enrichment analyses

TMM-normalized counts were used to perform SOM clustering as described previously (Chitwood *et al.*, 2013). After selecting differentially expressed genes within the top 25% of the coefficient of variation across samples, scaled expression values were used for multilevel three-by-four hexagonal SOM clustering. One hundred training interactions were used during the clustering, over which the α learning rate was decreased from 0.0036 to 0.0023. The final assignment of genes to winning units formed the basis of the gene clusters. GO term enrichment analyses were performed using PANTHER from the Gene Ontology Consortium.

Quantification of PRR Levels

The intensities of bands in immunoblotting images were quantified using ImageJ software (https://imagej.net/ij/), and statistical analyses were performed with GraphPad Prism 6 software (GraphPad Software, San Diego, CA, USA).

References

Anders S, Pyl PT, Huber W. 2015. HTSeq-a Python framework to work with high-throughput sequencing data. *Bioinformatics* **31**(2): 166-169.

Chitwood DH, Maloof JN, Sinha NR. 2013. Dynamic Transcriptomic Profiles between Tomato and a Wild Relative Reflect Distinct Developmental Architectures. *Plant Physiology* **162**(2): 537-552.

Felsenstein J. 1985. Confidence Limits on Phylogenies: An Approach Using the Bootstrap. *Evolution* **39**(4): 783-791.

Gong Y, Desveaux D, Guttman DS, Lewis JD. 2017. A Practical Guide to Quantitative Interactor Screening with Next-Generation Sequencing (QIS-Seq). *Methods Mol Biol* **1613**: 1-20.

Goto Y, Maki N, Ichihashi Y, Kitazawa D, Igarashi D, Kadota Y, Shirasu K. 2020. Exogenous Treatment with Glutamate Induces Immune Responses in Arabidopsis. *Mol Plant Microbe Interact* **33**(3): 474-487.

Goto Y, Maki N, Sklenar J, Derbyshire P, Menke FLH, Zipfel C, Kadota Y, Shirasu K. 2023. The phagocytosis oxidase/Bem1p domain-containing proteinPB1CP negatively regulates the NADPH oxidase RBOHD inplant immunity. *New Phytologist*: 2023 accepted.

Hancock JF, Paterson H, Marshall CJ. 1990. A polybasic domain or palmitoylation is required in addition to the CAAX motif to localize p21ras to the plasma membrane. *Cell* **63**(1): 133-139.

Ichihashi Y, Kusano M, Kobayashi M, Suetsugu K, Yoshida S, Wakatake T, Kumaishi K, Shibata A, Saito K, Shirasu K. 2018. Transcriptomic and Metabolomic Reprogramming from Roots to Haustoria in the Parasitic Plant, Thesium chinense. *Plant and Cell Physiology* **59**(4): 729-738.

Kadota Y, Liebrand TWH, Goto Y, Sklenar J, Derbyshire P, Menke FLH, Torres MA, Molina A, Zipfel C, Coaker G, et al. 2019. Quantitative phosphoproteomic analysis reveals common regulatory mechanisms between effector- and PAMP-triggered immunity in plants. *New Phytol* **221**: 2160–2175.

Kadota Y, Sklenar J, Derbyshire P, Stransfeld L, Asai S, Ntoukakis V, Jones JD, Shirasu K, Menke F, Jones A, et al. 2014. Direct regulation of the NADPH oxidase RBOHD by the PRR-associated kinase BIK1 during plant immunity. *Mol Cell* **54**(1): 43-55.

Lewis JD, Wan J, Ford R, Gong Y, Fung P, Nahal H, Wang PW, Desveaux D, Guttman DS. 2012. Quantitative Interactor Screening with next-generation Sequencing (QIS-Seq) identifies Arabidopsis thaliana MLO2 as a target of the Pseudomonas syringae type III effector HopZ2. *BMC Genomics* **13**: 8.

Ngou BPM, Heal R, Wyler M, Schmid MW, Jones JDG. 2022. Concerted expansion and contraction of immune receptor gene repertoires in plant genomes. *Nat Plants* **8**(10): 1146-1152.

Ngou BPM, Wyler M, Schmid MW, Kadota Y, Shirasu K. 2024. Evolutionary trajectory of pattern recognition receptors in plants. *Nat Commun* **15**(1): 308.

Robinson MD, McCarthy DJ, Smyth GK. 2010. edgeR: a Bioconductor package for differential expression analysis of digital gene expression data. *Bioinformatics* **26**(1): 139-140.

Saitou N, Nei M. 1987. The neighbor-joining method: a new method for reconstructing phylogenetic trees. *Mol Biol Evol* **4**(4): 406-425.

Schindelin J, Arganda-Carreras I, Frise E, Kaynig V, Longair M, Pietzsch T, Preibisch S, Rueden C, Saalfeld S, Schmid B, et al. 2012. Fiji: an open-source platform for biological-image analysis. *Nat Methods* **9**(7): 676-682.

Tamura K, Stecher G, Kumar S. 2021. MEGA11 Molecular Evolutionary Genetics

Analysis Version 11. *Molecular Biology and Evolution* **38**(7): 3022-3027.

Townsley BT, Covington MF, Ichihashi Y, Zumstein K, Sinha NR. 2015. BrAD-seq: Breath Adapter Directional sequencing: a streamlined, ultra-simple and fast library preparation protocol for strand specific mRNA library construction. *Frontiers in Plant Science* **6**.

Trapnell C, Pachter L, Salzberg SL. 2009. TopHat: discovering splice junctions with RNA-Seq. *Bioinformatics* **25**(9): 1105-1111.

Wang Y, Li J, Hou S, Wang X, Li Y, Ren D, Chen S, Tang X, Zhou JM. 2010. A Pseudomonas syringae ADP-ribosyltransferase inhibits Arabidopsis mitogen-activated protein kinase kinases. *Plant Cell* **22**(6): 2033-2044.

Zhou J, Wu S, Chen X, Liu C, Sheen J, Shan L, He P. 2014. The Pseudomonas syringae effector HopF2 suppresses Arabidopsis immunity by targeting BAK1. *Plant J* **77**(2): 235-245.

Zipfel C, Robatzek S, Navarro L, Oakeley EJ, Jones JD, Felix G, Boller T. 2004. Bacterial disease resistance in Arabidopsis through flagellin perception. *Nature* **428**(6984): 764-767.
