# Supplementary material for: The leucine-rich repeat receptor kinase QSK1 regulates PRR-RBOHD complexes targeted by the bacterial effector HopF2Pto
Source: Plant Cell. 2024 Oct 21;36(12):4932–51. doi: 10.1093/plcell/koae267 (PMC11641854; doi:10.1093/plcell/koae267)
Supplement: koae267_Supplementary_Data [file tpc.00647.2024-s17.pdf › tpc.00647.2024-s17.pdf]

THE PLANT CELL

The leucine-rich repeat receptor kinase QSK1 is a novel regulator of PRR-RBOHD complex and is employed by the bacterial effector HopF2Pto to modulate plant immunity

Yukihisa Goto, Yasuhiro Kadota, Malick Mbengue, Jennifer Lewis, Hidenori Matsui, Noriko Maki, Bruno Pok Man Ngou, Jan Sklenar, Paul Derbyshire, Arisa Shibata, Yasunori Ichihashi, David Guttman, Hirofumi Nakagami, Takamasa Suzuki, Frank Menke, Silke Robatzek, Darrell Desveaux, Cyril Zipfel, and Ken Shirasu

Review Timeline:

Submission Date:	12-Dec-2023
Editorial Decision:	28-Jan-2024
Resubmission Received:	19-Jul-2024
Editorial Decision:	11-Aug-2024
Revision Received:	13-Aug-2024
Accepted:	13-Aug-2024

Prof. Ken Shirasu
Rikagaku Kenkyujo
Center for Sustainable Resource Science
Tsukuba 230-0045
Japan

Dear Dr. Shirasu:

Thank you for choosing to send your manuscript entitled "The leucine-rich repeat receptor kinase QSK1 is a novel regulator of PRR-RBOHD complex and is employed by the bacterial effector HopF2Pto to modulate plant immunity" for consideration at The Plant Cell. Your submission has been evaluated by members of the editorial board as well as expert reviewers in your field, and we regret to inform you that we are not able to recommend the publication of this manuscript in its current form. We have had input from multiple scientists and we have solicited post-review comments.

That said, we appreciate the discovery of QSK1 as a novel PTI regulator, and we would be willing to re-consider a revised manuscript that fully addressed the concerns raised by the three reviewers. Most importantly, the revision should address whether QSK1 affects RBOHD levels and ROS production, and whether it interacts with BAK1.

If you decide to resubmit a revision, it may be treated as a new submission, but we would attempt to use at least some of the same reviewers. Nevertheless, reviewers will be asked to assess as a new manuscript (i.e. are the claims fully supported by the data and do the results presented move the field forward?), and not only whether previous reviewer comments have been addressed.

It will be important to convince the editors and reviewers that the study adds significant new understanding(s) of mechanisms or processes, or otherwise represents a significant advance in the field, and that the major claims made are fully justified by the data presented. This includes careful consideration and explanation of the various controls used in experiments, the extent and manner of replication, and the statistical analyses used. Sampling methods and the nature of "biological replicates" should be described precisely (i.e. different plants, parts of plants, pooled tissue, independent pools of tissue, sampled at different times, etc.), along with a clear description of and rationale for any statistical analyses conducted. The reader should know exactly what was sampled; what forms the basis of the calculation of any means and other statistical variables and parameters reported. This is also necessary to ensure that proper statistical analysis was conducted.

The Plant Cell now requires authors to complete and submit an author revisions checklist upon submission of a revised manuscript. The aim of the checklist is to aid authors in preparing a high-quality manuscript, facilitate the review and assessment of revised manuscripts, and help to ensure that journal standards are maintained across the board. If your manuscript is accepted, the completed checklist will be published as supplemental material attached to the article online. Please download a copy of the checklist (a fillable PDF form) at this link, for submission with your revised manuscript: https://tpc.msubmit.net/html/Author_Revisions_Checklist.pdf.

Note also that supplemental materials should be restricted to large datasets and tables, presentation of replicates, and validation of reagents, methods, or genotypes. Any data that are used to support the main claims must be in the main manuscript. Supplemental figure legends must indicate what figure in the main manuscript is supported by the supplemental data presented.

We thank you for your interest in and support of The Plant Cell. We hope we will be able to render a more positive decision on future work.

Sincerely,

The Plant Cell Editorial Board

-- Reviewer comments:

Reviewer #1 (Comments for the Author):

In this manuscript, "The leucine-rich repeat receptor kinase QSK1 is a novel regulator of PRR-RBOHD complex and is employed by the bacterial effector HopF2Pto to modulate plant immunity", the authors identified a negative regulator of PTI, QSK1, which could control the abundance of FLS2 and EFR. The authors also found that the bacterial effector HopF2Pto employed QSK1 to decrease plant immunity. Overall, this manuscript provides an interesting new component of the PRRs complex. However, I

have a few comments which might be helpful to improve the current version of this manuscript.

1. Compared with the wild type, qsk1 mutants displayed considerably higher ROS production after flg22 and elf18 treatment (Figures 3A and 3B). In contrast, only slightly increased MAPK phosphorylation was observed in qsk1 mutants after flg22 treatment (Figure 3C), suggesting that QSK might mainly function on ROS production, especially the authors found that QSK1 interacted with RBOHD too. To test it, I will suggest adding one more elicitor, for example, chitin, which is recognized by another type of PRR, lysM-RLK. If QSK regulates ROS production by mediating FLS2 degradation, chitin-induced ROS will not be increased in qsk1 mutants.
2. Since QSK1 was identified by isolating the proteins that simultaneously interact with PRRs (FLS2/FER) and RBOHD, I think it is worth determining whether QSK1 regulates RBOHD protein abundance.
3. To determine whether QSK1-regulated PTI signaling contributed to disease resistance, the authors inoculated plants with Pto DC3000 COR- and Pci strains (Figures 3D and 3E). Why did not use Pto DC3000 hrcC, a strain without ETI? Did qsk1 mutants show enhanced resistance to Pto DC3000 and Pto DC3000 hrcC?
4. Figure S3, the authors detected the pep1-induced ROS burst in qsk1/pQSK1:QSK1-GFP lines to validate whether QSK1-GFP transgene complemented qsk1 phenotype. I will suggest adding the results about flg22- and efl18-induced ROS production to be consistent with those in Figure 3.
5. To investigate whether QSK1 exerted a negative regulatory effect on PRR protein accumulation, the authors detected the FLS2 protein abundance in qsk1 mutants without elicitor treatment (Figure 5A). It would be better to determine further whether the increase of FLS2 abundance in qsk1 mutants was altered or not after flg22 treatment. Same for EFR, does QSK1 play a negative role in EFR abundance after elf18 treatment?
6. Figure 5G, the authors conclude that QSK1 overexpression may facilitate vacuolar degradation of PRRs through the autophagy pathway or the endocytosis pathway, according to the results with Con A treatment. If FLS2 and EFR proteins were regulated by vacuolar degradation, why did not conA treatment increase their protein levels (line 3 vs line 1 in Figure 5G)?
7. Flg22-induced QSK transcripts increase a lot (Figure S6). Why didn't flg22 induce their protein levels (Figure 2A input)?
8. Figure 7A, compared with the wild type HopF2pto, why did D175A mutation significantly reduce HopF2Pto protein abundance? When HopF2PtoD175A-HA levels were low, it was hard to tell whether the D175A mutation reduced HopF2Pto suppression on FLS2 protein abundance.
9. If HopF2Pto-mediated FLS2 protein degradation depended on QSK1 (Figure 10A), why conA did not block HopF2Pto-mediated FLS2 degradation (Figure S8), but it blocked QSK1-mediated FLS2 degradation (Figure 5G).
10. Figure 10C, if HopF2Pto-HA mediated FLS2 protein degradation, why were the FLS2 protein levels higher 24 h after inoculation with Pto DC3000 ΔHopF2 HopF2Pto-HA?
11. Figure 10D, to determine whether HopF2Pto-mediated FLS2 protein degradation contributed to disease resistance, the authors need to detect the bacterial growth of Pto DC3000 ΔHopF2 HopF2Pto-HA in fls2 mutants in addition to qsk1 mutants.

Reviewer #2 (Comments for the Author):

Authors identified that QSK1, which is a component of the PRR-RBOH complex, negatively regulates pattern-triggered immunity by downregulating the accumulation of PRRs. Moreover, the pathogen's effector, HopF2pto targets QSK1 and contributes to their virulence indicating QSK1 plays a pivotal role in plant immune responses.

Although I found the report to be very rich and full of data, however, I have a couple of questions about molecular mechanisms underlying the virulence function of HopF2pto through QSK1. I feel that the following details make the conclusions clearer.

1. The phenotype of mutants lacking QSK1 does not seem to affect plant growth. Why is this gene, which can be categorize into S gene, conserved in many plants?

2. While overexpression or deficit of QSK1 did not affect the transcript level of FLS2 (Fig, 5C), Dex-induced expression of HopF2pto downregulates at the transcript level (Fig. 8). In this manuscript, authors only confirmed that QSK1 is required for its accumulation and virulence function (Fig. 10A). Please investigate the transcript level of FLS2 when HopF2pto expressed in qsk1 mutant background in order to confirm that transcriptional effect by HopF2pto required QSK1.

3. The result of the pharmacological assay indicated that HopF2pto reduces EFR accumulation via vacuolar degradation through either the autophagy pathway or the endocytosis pathway, but not FLS2 accumulation (Fig. 9). Please investigate the accumulation of EFR when HopF2pto expressed in qsk1 mutant background in order to confirm that EFR protein degradation required QSK1.

Reviewer #3 (Comments for the Author):

In this manuscript, the authors employed a comparative Immunoprecipitation-Mass Spectrometry (IP-MS) approach to discern proteins interacting with the Pattern Recognition Receptors (PRRs) FLS2, EFR, and RBOHD. They successfully identified a novel Leucine-Rich Repeat Receptor Kinase (LRR-RK) named OSK1, elucidating its negative regulatory role in plant PAMP-Triggered Immunity (PTI) signaling. Subsequent investigations uncovered the interaction between the bacterial effector HopF2 and OSK1, resulting in the stability of OSK1 protein. Consequently, this stability led to diminished transcription of PRR receptors FLS2 and EFR, thereby attenuating plant PTI signaling. However, the manuscript lacks an exploration of how OSK1 modulates the function of the PRR-RBOHD complex concerning its formation, separation, or signal transduction (see comments 4-6). Nevertheless, this is a very good study identifying a new negative regulator OSK1 in PTI signaling. The study represents another elegant example of new key immune component in PTI signaling, and provides evidence to show how phytopathogens exploit effectors to manipulate plant immunity. Furthermore, the manuscript is well organized and written, and the main conclusions are supported by multiple data and complementary approaches.

Several comments have been provided for the authors' consideration to further improve the manuscript:
1. In certain Western Blot (WB) images, the quantification of FLS2 and EFR protein levels in different genetic backgrounds appears qualitative rather than quantitative. Please quantify the protein amount of FLS2 and EFR.
2. In Figure 3C, there is an observed increase in MAPK activation at 15 minutes, whereas it is reduced at 30 minutes in osk1 mutant. Why?
3. Why the authors exclusively assessed the Pst Cor- and Pci-related disease phenotypes of the osk1 mutant? How about wild-type Pst DC3000?
4. Does OSK1 have kinase activity? If yes, 1) whether the kinase activity is required for OSK1-mediated immune responses, such as ROS, MAPK, and disease? 2) whether OSK1 can phosphorylate RBOHD?
5. Does OSK1 interact with BAK1? Or does OSK1 affect FLS2-BAK1 interaction? Because FLS2 and BAK1 also form a complex in the resting state, which is similar to OSK1-FLS2.
6. In the previous model, RBOHD was not complexed with FLS2 and EFR. BIK1 is released from the activated PRR complex and then binds to and phosphorylates RBOHD. Here, OSK1 can bind FLS2, EFR and RBOHD in the resting state, so do the authors think that OSK1 forms a trimer with RBOHD and FLS2 or EFR?
7. Since HopF2 mono-ADP-transferase enzymatic activity is required for plant immunity, HopU1 is another mono-ADP-transferase that regulate FLS2 transcripts via blocking the binding of receptor FLS2's mRNA with RNA-binding proteins. So did OSK1 is also required for HopU1-mediated FLS2 transcripts?
8. Since HopF2 regulates a subset of genes transcripts, but the mechanism is unknown. Compared to HopU1, how HopF2 regulates gene transcripts should be discussed in the discussion section.
9. Did the authors isolate another osk1 T-DNA mutants, which shows similar immune phenotypes?
10. Protein sizes should be included in each WB images.

Dear Prof. Xing Li, Senior Editor
Dear Prof. Blake Meyers, Editor-in-Chief, The Plant Cell

We sincerely appreciate the valuable comments and suggestions from you and the reviewers, which have greatly contributed to improving the quality of our manuscript. We have carefully considered each comment and have made the necessary revisions as outlined below. We think that these revisions adequately address comments from you and the reviewers.

Thank you for choosing to send your manuscript entitled "The leucine-rich repeat receptor kinase QSK1 is a novel regulator of PRR-RBOHD complex and is employed by the bacterial effector HopF2Pto to modulate plant immunity" for consideration at The Plant Cell. Your submission has been evaluated by members of the editorial board as well as expert reviewers in your field, and we regret to inform you that we are not able to recommend the publication of this manuscript in its current form. We have had input from multiple scientists and we have solicited post-review comments.
That said, we appreciate the discovery of QSK1 as a novel PTI regulator, and we would be willing to re-consider a revised manuscript that fully addressed the concerns raised by the three reviewers. Most importantly, the revision should address whether QSK1 affects RBOHD levels and ROS production, and whether it interacts with BAK1.

→ To address whether QSK1 affects RBOHD levels or not, we performed the following experiments. First, we checked the chitin-induced ROS production in *qsk1-1* mutant, the complementation lines (New Fig. S6A), and *p35S:QSK1-3xHA* lines (New Fig. S6B). As shown in the figure below, QSK1 does not affect chitin-induced ROS production, which argue against a general defect in the ROS-producing machinery (e.g. RBOHD accumulation).

New Fig.S6A

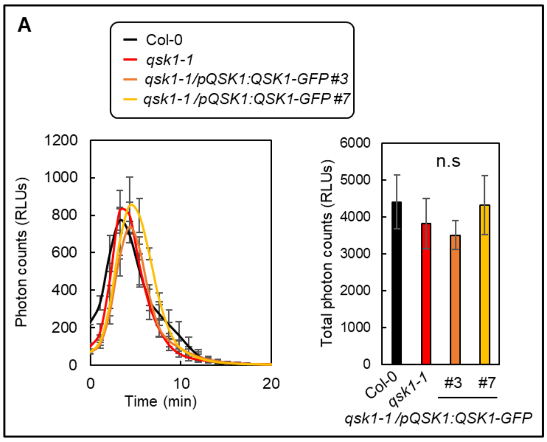

New Fig.S6B

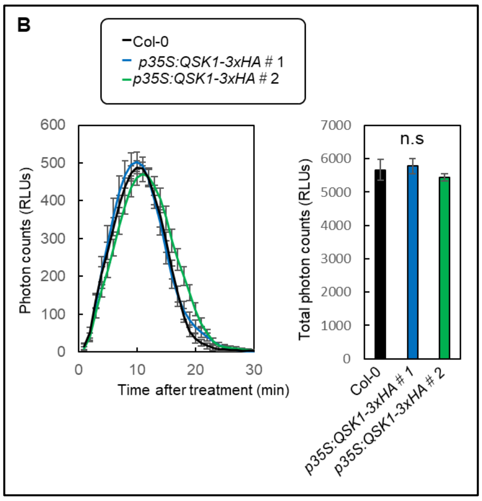

Next, we checked the RBOHD protein level in *qsk1-1* and *p35S:QSK1-3xHA#2* line. As shown in the figure below (New Fig. S10), there is no difference in RBOHD protein levels between these lines. Based on these results, we concluded that QSK1 does not regulate RBOHD protein levels.

New Fig. S10

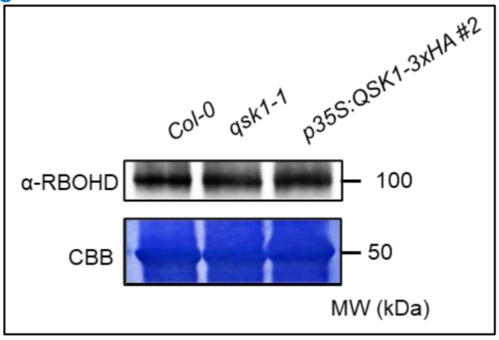

As requested by the editor and Reviewer 3, we checked the interaction of QSK1 with BAK1 using a co-IP assay (New Fig. S25). As shown in the figure, QSK1-3xHA is immunoprecipitated with BAK1, while QSK1 does not affect BAK1 protein levels (Figs. 5A and 5B). The interaction appears weak since longer exposure times are required to detect the QSK1 signal by western blotting. In cases where the interaction is strong, we observe a strong signal with minimal background noise. However, because the QSK1-BAK1 interaction is weak, longer exposure times are necessary, which sometimes results in increased nonspecific interactions (QSK1 interaction with beads rather than BAK1) despite extensive washing.

New Fig. S25

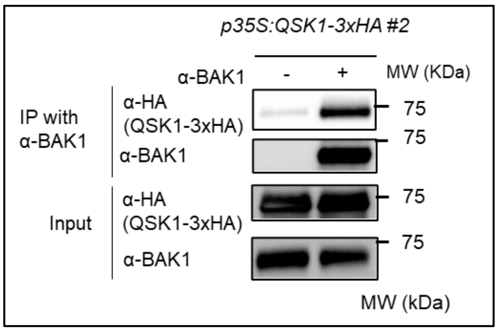

Nevertheless, we found that QSK1 is immunoprecipitated with EFR, FLS2, RBOHD, as well as BAK1 (new data). Thus, we hypothesize that the co-IP with FLS2, EFR, and RBOHD may isolate not only direct interactors but also the plasma membrane nanodomains where these proteins reside. Previous studies have shown that FLS2 and its associated components are localized in nanodomains at the plasma membrane (Bücherl et al., eLife, 2017; Gronnier et al., eLife, 2022), and at least some components of these nanodomains accumulate in detergent-resistant membranes (DRMs) upon flg22 treatment (Keinath et al., J Biol Chem, 2010). Indeed, our co-IP with EFR, FLS2, or RBOHD identified such flg22-inducible DRM proteins (REM1.2, QSK1, ACA10, SYP71, HIR1, and HIR4) independent of flg22 treatment (Figure 1B), suggesting the isolation of PRR-containing nanodomains. Thus, we toned down the mention of QSK1 as “an associated protein” rather than “an interactor”. We have carefully discussed this

point in the discussion section (Line 498-514).

We hope the revised manuscript satisfies you and all reviewers, and that our manuscript will be accepted for publication in The Plant Cell.

Thank you very much for your consideration.

Best regards,
Yasuhiro Kadota, on behalf of all the authors

Response to Reviewer #1

In this manuscript, "The leucine-rich repeat receptor kinase QSK1 is a novel regulator of PRR-RBOHD complex and is employed by the bacterial effector HopF2Pto to modulate plant immunity", the authors identified a negative regulator of PTI, QSK1, which could control the abundance of FLS2 and EFR. The authors also found that the bacterial effector HopF2Pto employed QSK1 to decrease plant immunity. Overall, this manuscript provides an interesting new component of the PRRs complex. However, I have a few comments which might be helpful to improve the current version of this manuscript.

→ We sincerely appreciate the valuable comments provided by Reviewer 1, which have greatly contributed to improving the quality of our manuscript. We have carefully considered each comment and have made the necessary revisions as outlined below. We think that these revisions adequately address Reviewer 1's comments.

1. Compared with the wild type, qsk1 mutants displayed considerably higher ROS production after flg22 and elf18 treatment (Figures 3A and 3B). In contrast, only slightly increased MAPK phosphorylation was observed in qsk1 mutants after flg22 treatment (Figure 3C), suggesting that QSK might mainly function on ROS production, especially the authors found that QSK1 interacted with RBOHD too. To test it, I will suggest adding one more elicitor, for example, chitin, which is recognized by another type of PRR, lysM-RLK. If QSK regulates ROS production by mediating FLS2 degradation, chitin-induced ROS will not be increased in qsk1 mutants.

→Thanks very much for pointing this out. As you can see in the response to the editor, we checked the chitin-induced ROS production in *qsk1-1* mutant, the complementation lines (New Fig. S6A), and *p35S:QSK1-3xHA#2* line (New Fig. S6B). The data shows that QSK1 does not affect chitin-induced ROS production, thus arguing against a general defect in the ROS-producing machinery and against a potential role in BAK1-independent PRR signaling.

2. Since QSK1 was identified by isolating the proteins that simultaneously interact with PRRs (FLS2/FER) and RBOHD, I think it is worth determining whether QSK1 regulates RBOHD protein abundance.

→Thanks very much for pointing this out. As you can see in the response to the editor, we checked RBOHD protein level in *qsk1-1* and *p35S:QSK1-3xHA#2* line (New Fig. S10), and found that there is no difference in RBOHD protein levels between these lines.

3. To determine whether QSK1-regulated PTI signaling contributed to disease resistance, the authors inoculated plants with Pto DC3000 COR- and Pci strains (Figures 3D and 3E). Why did not use Pto DC3000 hrcC, a strain without ETI? Did qsk1 mutants show enhanced resistance to Pto DC3000 and Pto DC3000 hrcC?

→ Thank you for pointing this out. We used *Pto* DC3000 *COR*$^-$ and *Pci* strains as we have observed a significant effect of PTI on resistance against these bacteria (Kadota et al., Molecular Cell, 2014). As requested, we also tested with *Pto* DC3000 and *Pto* DC3000 *hrcC-* (New Fig.S3 and S23). As shown in the figures below (New Fig.S3), *qsk1-1* is more resistant but *p35S:QSK1-3xHA#2* is less resistant to *Pto* DC3000 *hrcC*$^-$. This result is consistent with the data for *Pto* DC3000 *COR*$^-$ and *Pci* (Fig. 3D, 3E, 4D, and 4E). Similarly, *qsk1-1* is more resistant, but *p35S:QSK1-3xHA#2* is less resistant to *Pto* DC3000 *ΔhopF2* complemented with *HopF2*$_{Pto}$*-HA* (*Pto* DC3000 *ΔhopF2 HopF2*$_{Pto}$*-HA*) (New Fig. 10F). However, *qsk1-1* and *p35S:QSK1-3xHA#2* are similarly susceptible to *Pto* DC3000 compared to Col-0 (New Fig.S23). This difference in resistance may be attributed to the native *HopF2*$_{Pto}$ possessing an ATA start codon, which limits its expression. Therefore, the contribution of HopF2$_{Pto}$ is not as significant in *Pto* DC3000 wild type relative to *Pto* DC3000 *ΔhopF2* complemented with *HopF2*$_{Pto}$*-HA* (ATG). We carefully discuss this point in the discussion (Line 673-682).

New Fig. S3

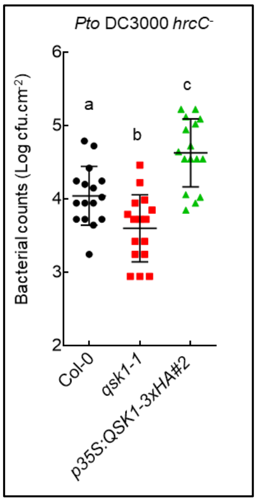

New Fig. 10F

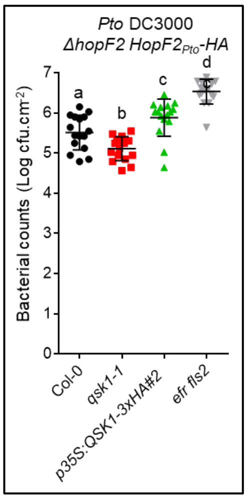

New Fig. S23

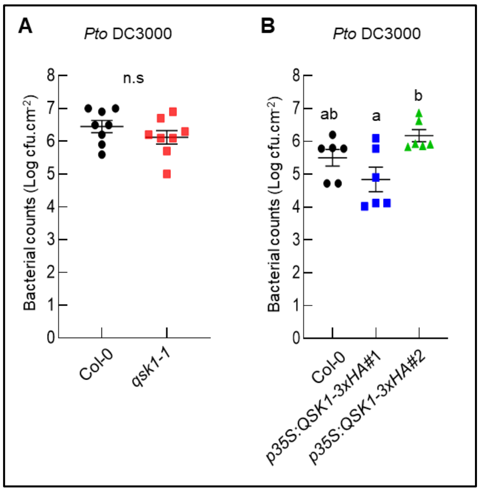

4. Figure S3, the authors detected the pep1-induced ROS burst in qsk1/pQSK1:QSK1-GFP lines to validate whether QSK1-GFP transgene complemented qsk1 phenotype. I will suggest adding the results about flg22- and efl18-induced ROS production to be consistent with those in Figure 3.

→ Thank you for pointing this out. As suggested, we checked flg22- and efl18-induced ROS production in the complementation lines (New Fig.S4B, S4C). Together with the data of pep2-induced ROS burst, we concluded that QSK1-GFP transgene complemented *qsk1* mutant phenotype.

New Fig.S4B

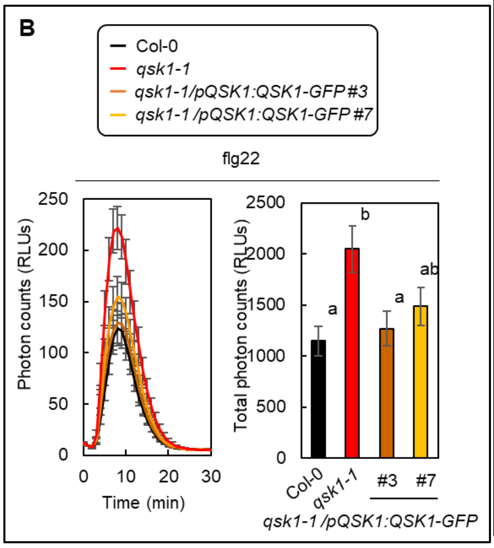

New Fig.S4C

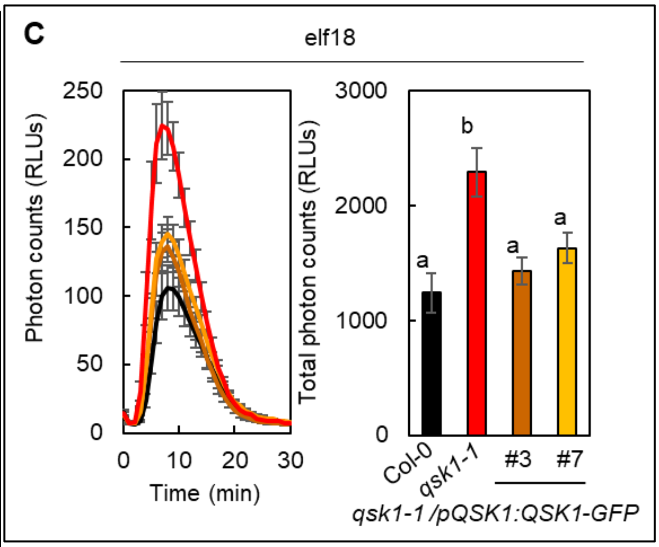

5. To investigate whether QSK1 exerted a negative regulatory effect on PRR protein accumulation, the authors detected the FLS2 protein abundance in qsk1 mutants without elicitor treatment (Figure 5A). It would be better to determine further whether the increase of FLS2 abundance in qsk1 mutants was altered or not after flg22 treatment. Same for EFR, does QSK1 play a negative role in EFR abundance after elf18 treatment?

→ Thank you for pointing this out. As suggested, we checked the effect of QSK1 on the abundance of FLS2, EFR and BAK1 after elicitation. We treated Col-0, *qsk1-1*, and *p35S:QSK1-3xHA #2* with 10 μM flg22 for 1 h, and checked FLS2 and BAK1 protein abundance by immunoblotting (New Fig.S11A and S11B).

New Fig.S11A

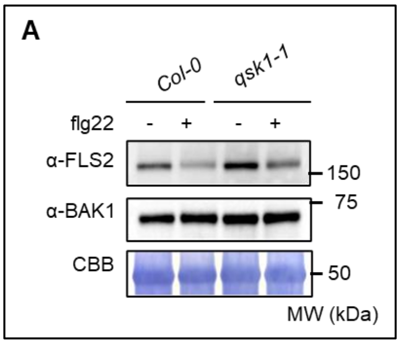

New Fig.S11B

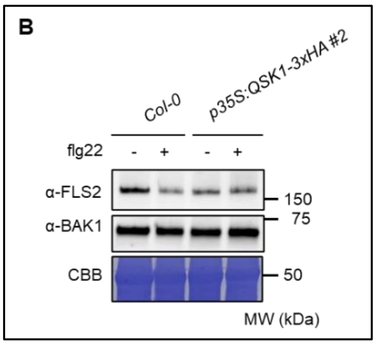

FLS2 protein level decreased in Col-0 1 h after flg22 treatment, possibly due to the protein degradation following endocytosis (Robatzek et al., Genes Dev 2006; 20:537-42). The treatment with flg22 also decreased FLS2 protein abundance in *qsk1-1* line, although the basal FLS2 protein level was higher in *qsk1-1* than in Col-0. In contrast, the basal FLS2 protein level was lower in *p35S:QSK1-3xHA #2*, and the treatment with flg22 did not further reduce FLS2 protein abundance. This might suggest that some FLS2 protein detected by WB in *p35S:QSK1-3xHA #2* is not functional or does not exist at the plasma membrane.

We also checked EFR-GFP abundance in *pEFR:EFR-GFP* and *pEFR:EFR-GFP/p35S:QSK1-3xHA#2* after treatment with 10 µM flg22 for 1 h (New Fig.S11C). EFR-GFP level decreased in *pEFR:EFR-GFP* 1 h after elf18 treatment. In contrast, the basal EFR-GFP protein level was lower in *pEFR:EFR-GFP/p35S:QSK1-3xHA#2*, and the treatment with elf18 did not further reduce EFR-GFP protein abundance.

New Fig.S11C

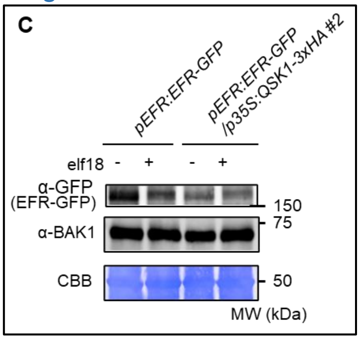

6. Figure 5G, the authors conclude that QSK1 overexpression may facilitate vacuolar degradation of PRRs through the autophagy pathway or the endocytosis pathway, according to the results with Con A treatment. If FLS2 and EFR proteins were regulated by vacuolar degradation, why did not conA treatment increase their protein levels (line 3 vs line 1 in Figure 5G)?

→ Thank you for pointing this out. We saw a very minor increase of FLS2 and EFR-GFP in *pEFR:EFR-GFP* after the treatment with ConA (line 3 vs line 1 in New Figure 5H). We think that the QSK1-mediated negative regulation of FLS2 and EFR through vacuolar degradation is weaker in *pEFR:EFR-GFP* than in *pEFR:EFR-GFP/p35S:QSK1-3xHA* line due to the lower QSK1 level. Therefore, the effect of ConA on the abundance of FLS2 and EFR-GFP may be weaker in *pEFR:EFR-GFP* than in *pEFR:EFR-GFP/p35S:QSK1-3xHA* line. We carefully discuss this point in the result (Line 307-311).

New Fig.5H

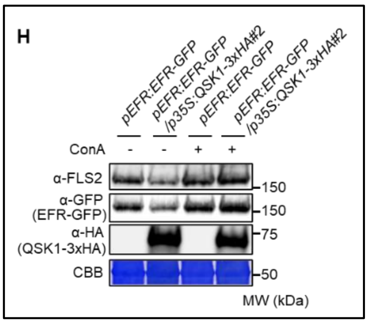

7. Flg22-induced QSK transcripts increase a lot (Figure S6). Why didn't flg22 induce their protein levels (Figure 2A input)?

→ qRT-PCR analysis was done using seedlings treated with flg22 for 6 h, while IP was performed using seedlings treated with flg22 for 10 min. We think the treatment with flg22 for 10 min is too short to increase the QSK1 protein level.

8. Figure 7A, compared with the wild type HopF2pto, why did D175A mutation significantly reduce HopF2Pto protein abundance? When HopF2PtoD175A-HA levels were low, it was hard to tell whether the D175A mutation reduced HopF2Pto suppression on FLS2 protein abundance.

→ Thank you for pointing this out. As Reviewer1 indicated, the D175A protein itself may be unstable, or the transgenic *pDEX:HopF2$_{Pto}$-HA D175A* line may not produce HopF2$_{Pto}$-HA (D175A) as efficiently as *pDEX: HopF2$_{Pto}$ -HA* WT line. To address this, we treated both lines with different DEX concentrations to find the optimal DEX concentration that induces HopF2$_{Pto}$ -HA protein synthesis at similar levels (New Fig. S15).

New Fig.S15

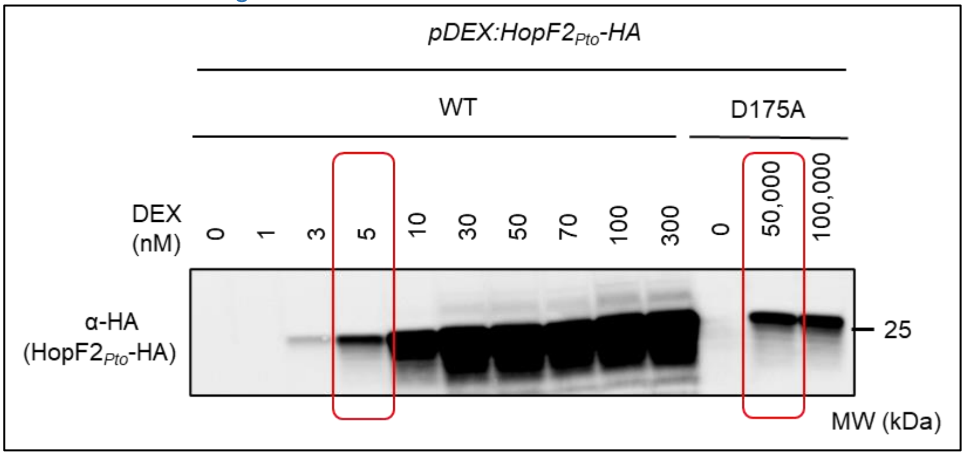

Using this condition, we compared FLS2 protein abundance in *pDEX:HopF2$_{Pto}$-HA* and *pDEX:HopF2$_{Pto}$-HA* (D175A) (New Fig. 7A) after treatment with DEX. As shown in the figure below (New Fig. 7A), both lines produced similar levels of HopF2$_{Pto}$ (WT) and HopF2$_{Pto}$ (D175A), but HopF2$_{Pto}$ (WT), but not HopF2$_{Pto}$ (D175A), reduces FLS2 abundance, highlighting the importance of D175 catalytic residue of HopF2$_{Pto}$ to induce a reduction in FLS2 protein levels.

New Fig.7A

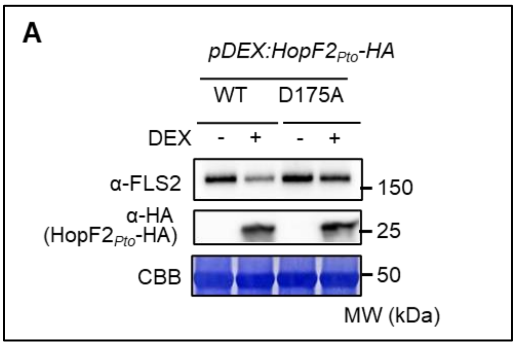

9. If HopF2Pto-mediated FLS2 protein degradation depended on QSK1 (Figure 10A), why conA did not block HopF2Pto-mediated FLS2 degradation (Figure S8), but it blocked QSK1-mediated FLS2 degradation (Figure 5G).

→ Thank you for pointing this out. This is because HopF2*Pto* reduces transcript levels of *FLS2* (Fig. 8A). As shown in Fig. 8A, *FLS2* transcript levels are reduced by 80 % after treatment with Dex for 24 h. In Fig. S16, we treated *pDEX:HopF2Pto-HA* with DEX for 24 h and then treated with ConA. Even though ConA would block QSK1-mediated FLS2 degradation, *pDEX:HopF2Pto* line may not have enough *FLS2* transcripts for *de novo* synthesis of FLS2 protein. In contrast, HopF2*Pto* does not reduce transcript levels of *EFR* and ConA effectively countered the HopF2*Pto*-mediated reduction in EFR protein levels. We carefully explain this in the result section (Line 453-457).

10. Figure 10C, if HopF2Pto-HA mediated FLS2 protein degradation, why were the FLS2 protein levels higher 24 h after inoculation with Pto DC3000 ΔHopF2 HopF2Pto-HA?

→ Thank you for pointing this out. When using *pDEX:HopF2Pto-HA* line, DEX treatment depletes FLS2 protein abundance (Fig. 6D) and eliminates flg22-mediated responses (Fig. S19 and S20). However, in natural infections, multiple PAMPs from *Pseudomonas* rapidly trigger PTI responses, leading to the transcriptionally upregulation of PRRs and subsequent FLS2 accumulation. The bacteria then inject HopF2*Pto* into plant cells at a later stage to reduce FLS2 abundance. Thus, in the natural infection process, HopF2*Pto* works to suppress PAMP-inducible FLS2 accumulation. We carefully explain this in the results (Line 363-371).

11. Figure 10D, to determine whether HopF2Pto-mediated FLS2 protein degradation contributed to disease resistance, the authors need to detect the bacterial growth of Pto DC3000 ΔHopF2 HopF2Pto-HA in fls2 mutants in addition to qsk1 mutants.

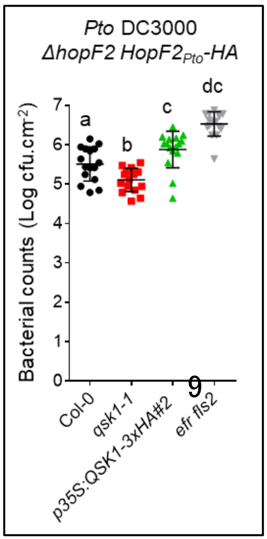

→ Thank you for pointing this out. As suggested, we repeated the patho-test using *efr fls2* double mutant. We chose *efr fls2* line as QSK1 reduces both EFR and FLS2 protein levels (New Fig.10F).

New Fig.10F

Response to Reviewer #2

Authors identified that QSK1, which is a component of the PRR-RBOH complex, negatively regulates pattern-triggered immunity by downregulating the accumulation of PRRs. Moreover, the pathogen's effector, HopF2pto targets QSK1 and contributes to their virulence indicating QSK1 plays a pivotal role in plant immune responses. Although I found the report to be very rich and full of data, however, I have a couple of questions about molecular mechanisms underlying the virulence function of HopF2pto through QSK1. I feel that the following details make the conclusions clearer.

→ We sincerely appreciate the valuable comments provided by Reviewer #2, which have greatly contributed to improving the quality of our manuscript. We have carefully considered each comment and have made the necessary revisions as outlined below. We think that these revisions adequately address Reviewer #2's comments and our conclusions became clearer.

1. The phenotype of mutants lacking QSK1 does not seem to affect plant growth. Why is this gene, which can be categorize into S gene, conserved in many plants?

→ We think that QSK1 is important for the regulation of FLS2, which is conserved in most angiosperms. We performed phylogenic analyses of QSK1 (New Fig. S26) and found that QSK1 is also well-conserved in many angiosperms including basal angiosperms, monocots, and dicots.

New Fig.S26B

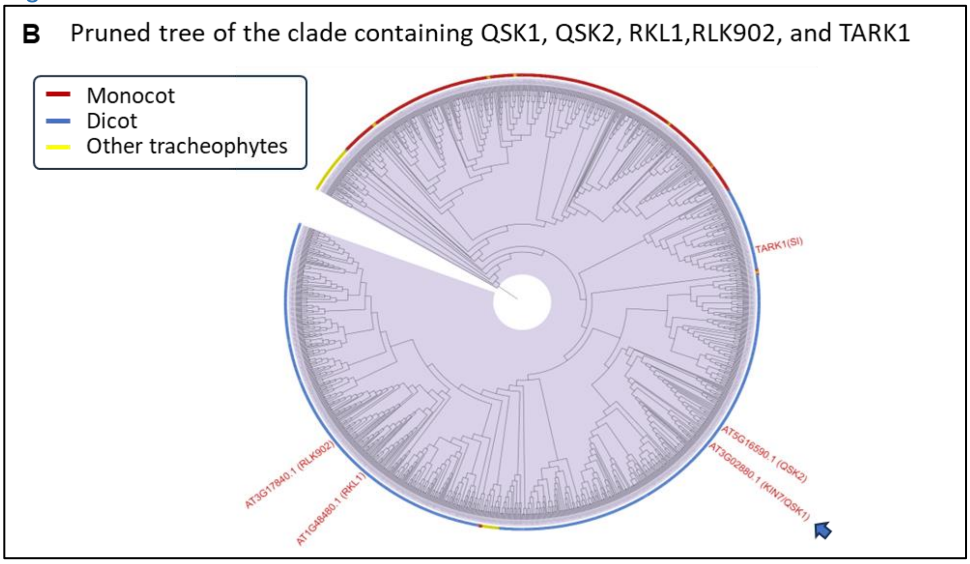

2. While overexpression or deficit of QSK1 did not affect the transcript level of FLS2 (Fig, 5C), Dex-induced expression of HopF2pto downregulates at the transcript level (Fig. 8). In this manuscript, authors only confirmed that QSK1 is required for its accumulation and virulence function (Fig. 10A). Please investigate the transcript level of FLS2 when HopF2pto expressed in qsk1 mutant background in order to confirm that transcriptional effect by HopF2pto required QSK1.

→ Thanks for pointing out this important point. As suggested, we checked the transcript levels of *FLS2* and *PROSCOOP8* in *pDEX:HopF2$_{Pto}$-HA* and *pDEX:HopF2$_{Pto}$-HA/qsk1-1* with or without DEX treatment (New Fig.10C). We chose *PROSCOOP8* because its expression is most significantly reduced by HopF2$_{Pto}$ among all *PROSCOOPs*. As shown in the figure below, QSK1 is required for the reduction of the transcripts of *FLS2* and *PROSCOOP8* by HopF2$_{Pto}$.

New Fig.10C

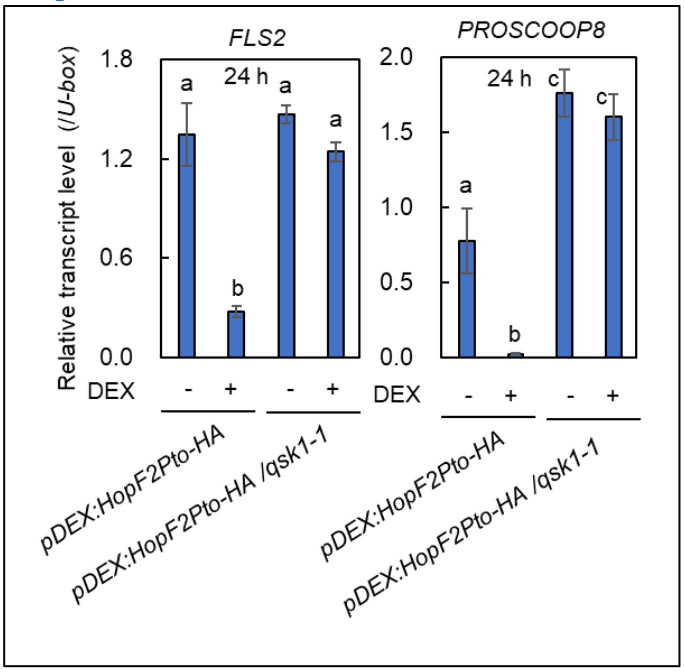

3. The result of the pharmacological assay indicated that HopF2pto reduces EFR accumulation via vacuolar degradation through either the autophagy pathway or the endocytosis pathway, but not FLS2 accumulation (Fig. 9). Please investigate the accumulation of EFR when HopF2pto expressed in qsk1 mutant background in order to confirm that EFR protein degradation required QSK1.

→Thanks for pointing out this important point. Unfortunately, we don't have *qsk1/Dex:HopF2*$_{\text{Pto}}$*-HA/EFR-GFP* homozygous line to investigate EFR accumulation. However, we have clear evidence that HopF2$_{Pto}$ reduces EFR protein levels (Fig. 9) and HopF2$_{Pto}$ protein level is reduced in *qsk1-1* mutant, suggesting that the reduction of EFR protein levels by HopF2$_{Pto}$ requires QSK1(Fig. 10A). However, we did not have clear evidence that QSK1 is required for HopF2$_{Pto}$ -mediated inhibition of PTI signaling pathways under many different PRRs such as FLS2, EFR, MIK2, and PEPR1/2. Therefore, we checked the effect of QSK1 on HopF2$_{Pto}$ -mediated inhibition of ROS production induced by activation of these PRRs (New Fig. 10D, New Fig. S21). As shown in the figures below, these data show that QSK1 is required for the inhibition of ROS production induced by flg22, SCOOP12, elf18, and pep2.

New Fig.10D

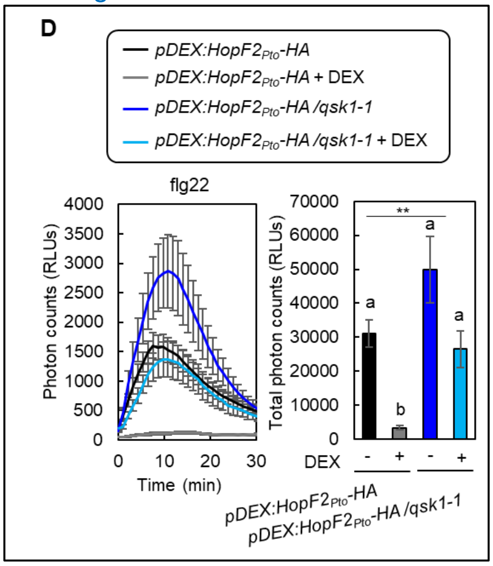

New Fig. S21

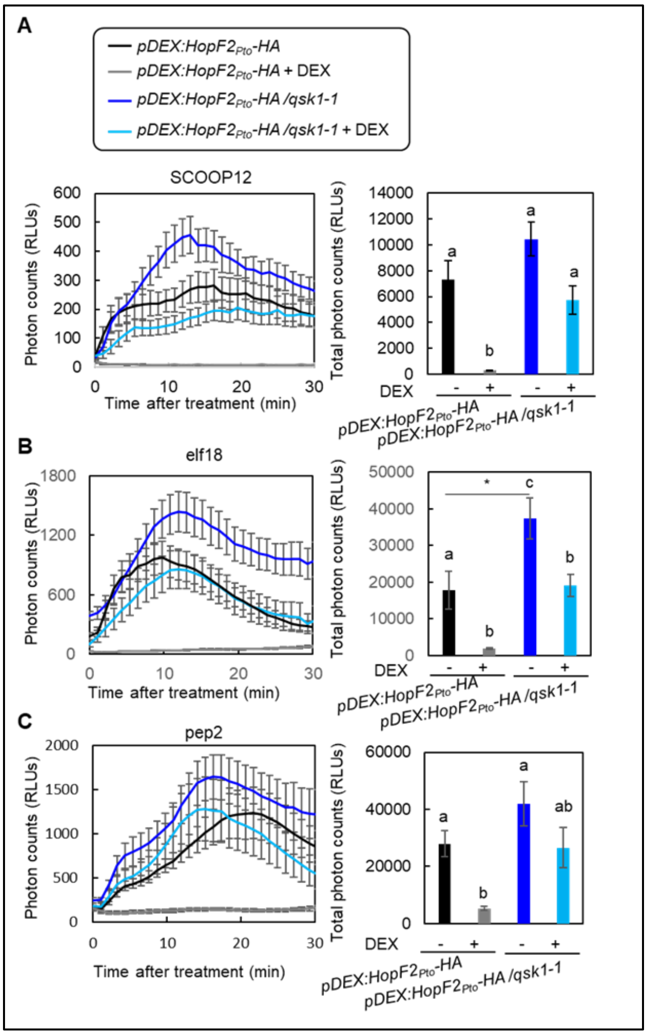

Response to Reviewer #3

In this manuscript, the authors employed a comparative Immunoprecipitation-Mass Spectrometry (IP-MS) approach to discern proteins interacting with the Pattern Recognition Receptors (PRRs) FLS2, EFR, and RBOHD. They successfully identified a novel Leucine-Rich Repeat Receptor Kinase (LRR-RK) named QSK1, elucidating its negative regulatory role in plant PAMP-Triggered Immunity (PTI) signaling. Subsequent investigations uncovered the interaction between the bacterial effector HopF2 and QSK1, resulting in the stability of QSK1 protein. Consequently, this stability led to diminished transcription of PRR receptors FLS2 and EFR, thereby attenuating plant PTI signaling. However, the manuscript lacks an exploration of how QSK1 modulates the function of the PRR-RBOHD complex concerning its formation, separation, or signal transduction (see comments 4-6). Nevertheless, this is a very good study identifying a new negative regulator QSK1 in PTI signaling. The study represents another elegant example of new key immune component in PTI signaling, and provides evidence to show how phytopathogens exploit effectors to manipulate plant immunity. Furthermore, the manuscript is well organized and written, and the main conclusions are supported by multiple data and complementary approaches. Several comments have been provided for the authors' consideration to further improve the manuscript:

→ We sincerely appreciate the valuable comments provided by Reviewer 3, which have greatly contributed to improving the quality of our manuscript. We have carefully considered each comment and have made the necessary revisions as outlined below. We believe that these revisions adequately address Reviewer 3's comments.

1. In certain Western Blot (WB) images, the quantification of FLS2 and EFR protein levels in different genetic backgrounds appears qualitative rather than quantitative. Please quantify the protein amount of FLS2 and EFR.

→ Thank you for pointing this out. As suggested by Reviewer3, we quantified FLS2 and EFR protein levels in *qsk1-1* mutant, *p35S:QSK1-3xHA#2* and *pDEX:HopF2*$_{Pto}$*-HA* line (New Fig. S9 and S14). As you can see in the figures below, the differences in FLS2 and EFR protein levels were statistically significant.

Fig. S9

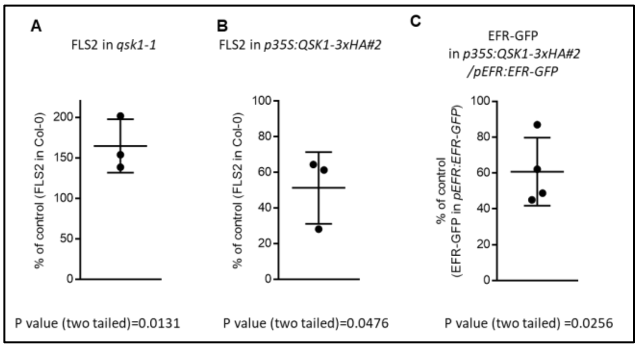

Fig.S14

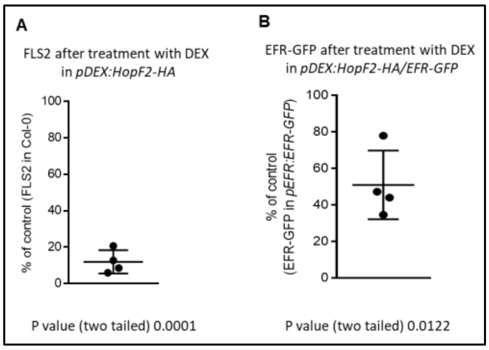

2. In Figure 3C, there is an observed increase in MAPK activation at 15 minutes, whereas it is reduced at 30 minutes in QSK1 mutant. Why?
→ We think that due to the higher accumulation of FLS2 in *qsk1-1* mutant, MAPK activation is induced more rapidly and strongly, leading to more rapid inactivation.
But, the exact mechanisms are unknown.

3. Why the authors exclusively assessed the Pst Cor- and Pci-related disease phenotypes of the qsk1 mutant? How about wild-type Pst DC3000?
Thank you for pointing this out. This is the same question posed by Reviewer 1. Please see our response to Comment 3 by Reviewer 1.

4. Does QSK1 have kinase activity?
→ Thank you for pointing this out. The kinase activity of QSK1 has been demonstrated in two previous papers (Wu et al., Mol Cell Proteomics, 2019, Aryal et al., Curr Biol, 2023), and autophosphorylation was shown in another paper (Nemoto et al., Phytochemistry, 2011).

If yes, 1) whether the kinase activity is required for QSK1-mediated immune responses, such as ROS, MAPK, and disease?
→ As suggested by Reviewer 3, we checked the importance of the kinase activity on the function of QSK1 to suppress PTI (New Fig. S12).

D488N mutation in QSK1 is thought to prevent the nucleophilic attack on the gamma-phosphate of the ATP molecule and thus reduces the activity of the enzyme to zero (Aryal et al., Current Biology 2023). We expressed QSK1-mCherry and QSK1(D488N)-mCherry in *N. benthamiana* and checked flg22-induced ROS production. As shown in the figure below (New Fig. S12A), QSK1-mCherry reduced flg22-induced ROS production to the GUS-expressed region in the same leaves. Unexpectedly, QSK1(D488N)-mCherry reduced flg22-induced ROS production much more effectively, suggesting that kinase activity is not required for this function (New Fig. S12B).

New Fig.S12

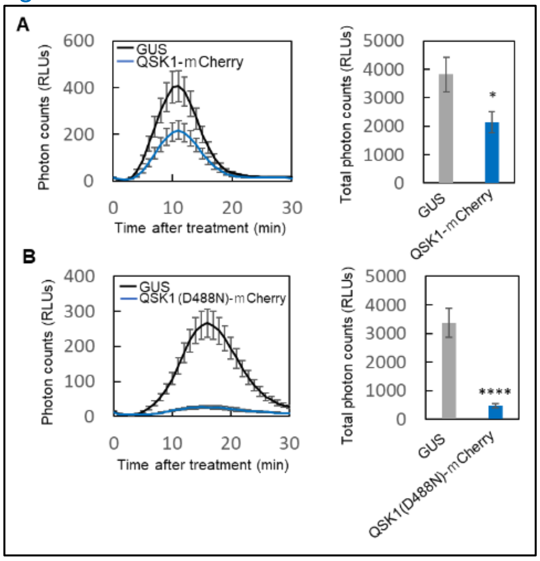

We also checked FLS2-3xmyc-GFP protein levels after co-expression with QSK1-mCherry or QSK1(D488N)-mCherry. QSK1-mCherry reduced FLS2-3xmyc-GFP protein accumulation (New Fig. S12A C). QSK1(D488N)-mCherry reduced FLS2-3xmyc-GFP protein accumulation much more effectively (New Fig. S12D). We compared the protein levels of QSK1-mCherry or QSK1(D488N)-mCherry expressed in the same leaf and found that QSK1(D488N)-mCherry protein level was much higher than QSK1-mCherry (New Fig. S12E). These results show that the kinase activity of QSK1 is not required for the regulation of FLS2 protein levels, and the QSK1 abundance is the key factor for FLS2 protein level reduction. As we menthioned in the discussion, QSK1 might function to compete with RGI for FLS2 interaction to inhibit GLV2-mediated RGI-FLS2 interactions, which causes the degradation of GLV2-unbound FLS2 (Stegmann et al., 2022 EMBO Rep). We carefully discuss this point in the results and discussion section (Line 552-570).

New Fig.S12

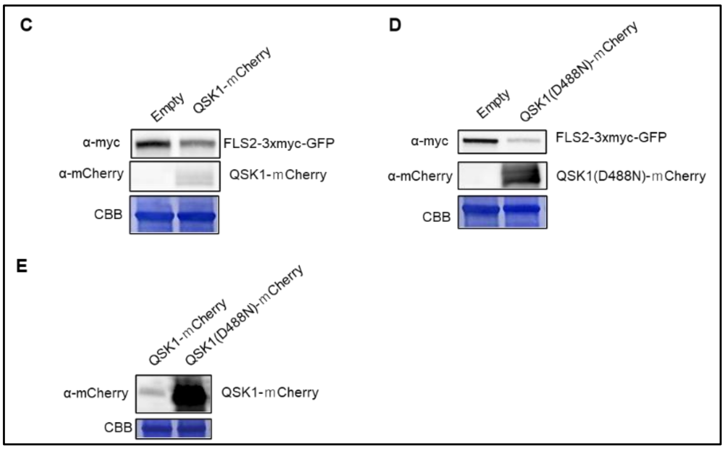

2) whether QSK1 can phosphorylate RBOHD?

→ As you can see in our responses to the Editor, we checked the chitin-induced ROS production in *qsk1-1* mutant, the complementation lines (New Fig. S6A), and *p35S:QSK1-3xHA#2* (New Fig. S6B), and found that QSK1 does not affect chitin-induced ROS production. We also checked the RBOHD protein levels in *qsk1-1* and *p35S:QSK1-3xHA#2*, and found that there is no difference in RBOHD protein levels between these lines. This series of new data shows that QSK1 does not regulate RBOHD protein levels.

5. Does QSK1 interact with BAK1? Or does QSK1 affect FLS2-BAK1 interaction? Because FLS2 and BAK1 also form a complex in the resting state, which is similar to QSK1-FLS2.

→ As you can see in our responses to the Editor, we checked the interaction of QSK1 to BAK1 by co-IP assay. The result shows that QSK1 also associates with BAK1 *in vivo*, while QSK1 does not affect BAK1 protein levels.

6. In the previous model, RBOHD was not complexed with FLS2 and EFR. BIK1 is released from the activated PRR complex and then binds to and phosphorylates RBOHD. Here, QSK1 can bind FLS2, EFR and RBOHD in the resting state, so do the authors think that QSK1 forms a trimer with RBOHD and FLS2 or EFR?

→ We observed that RBOHD forms complexes with EFR and FLS2 in the resting state, and this interaction remains unchanged upon PAMP treatment, while activated BIK1 exhibits stronger binding to RBOHD (Kadota et al., Mol Cell 2014, Figures 1B and 2D). In our current study, RBOHD was also identified in FLS2 immunoprecipitates with or without flg22 treatment (Supplementary Table S1_1 FLS2-IP).

Given that QSK1 was co-immunoprecipitated with EFR, FLS2, and RBOHD, it suggests that QSK1 could be part of the EFR-RBOHD and FLS2-RBOHD complexes. Additionally, QSK1 associates with BAK1 in the resting state (Supplemental Fig. S24), indicating QSK1's involvement in multiple protein complexes. However, QSK1 might not directly interact with EFR, FLS2, or RBOHD but may exist in the same nanodomains at the plasma membrane. Previous studies have shown that FLS2 and its associated components are localized in REM1.2-positive nanodomains at the plasma membrane (Bücherl et al., eLife, 2017). These nanodomains are thought to accumulate in detergent-resistant membranes (DRMs) upon flg22 treatment (Keinath et al., J Biol Chem, 2010). Indeed, our co-IP with EFR, FLS2, or RBOHD and LC-MS/MS analyses identified proteins that accumulate in DRMs in response to flg22, including QSK1, REM1.2, ACA10, SYP71, HIR1, and HIR4 (Fig. 1B). Additionally, rice RBOHB, which is orthologous to Arabidopsis RBOHD, was shown to exist in DRM in rice (Nagano et al., Plant Cell, 2016). These results suggest our co-IP may isolate not only PRR-containing protein complexes but also isolate components in immunity-specific nanodomains at the plasma membrane. We carefully discuss this point in the discussion (Line 497-514).

7. Since HopF2 mono-ADP-transferase enzymatic activity is required for plant immunity, HopU1 is another mono-ADP-transferase that regulate FLS2 transcripts via blocking the binding of receptor FLS2's mRNA with RNA-binding proteins. So did QSK1 is also required for HopU1-mediated FLS2 transcripts?

→ Thank you very much for your very nice suggestion! Although we are focusing on the relationship between QSK1 and HopF2$_{Pto}$ in this manuscript, it is definitely an interesting possibility to test in a future project.

8. Since HopF2 regulates a subset of transcripts, but the mechanism is unknown. Compared to HopU1, how HopF2 regulates gene transcripts should be discussed in the discussion section.

→ Thank you for pointing this out. HopF2$_{Pto}$ expression in plants reduces the basal transcript levels of PRRs such as FLS2, LORE, MIK2, and phytocytokines such as SCOOPs (Fig. 8). Additionally, it reduces basal FLS2 protein levels. In contrast, HopU1 expression in plants does not affect *FLS2* transcript levels or basal FLS2 protein levels (Fig. 4A and 6A in Nicaise et al., EMBO J. 2013). These results show that although both HopF2$_{Pto}$ and HopU1 encode ADP-ribosyl transferase and function to suppress FLS2 accumulation upon Pseudomonas infection, the mechanisms of FLS2 suppression differ significantly.

There are two possible mechanisms by which HopF2$_{Pto}$ regulates transcript levels. First, HopF2$_{Pto}$ may reduce the protein levels of phytocytokine receptors such as MIK2, possibly through vacuolar degradation similar to EFR and FLS2. Second, HopF2$_{Pto}$ has been shown to inhibit BAK1 and MKK5, which are important for many different peptide hormone signaling pathways, including SCOOPs, Peps, and RGIs (MKK5 might also be involved in RALF and CEP signaling). The reduction of phytocytokine receptors and the inhibition of peptide hormone signaling pathways likely suppress the baseline

expression of PRRs and phytocytokines such as PROSCOOPs. We have added a discussion about this point in the discussion section (Line 627-643).

9. Did the authors isolate another QSK1 T-DNA mutants, which show similar immune phenotypes?

→ As far as I know, there are only two T-DNA mutants of *qsk1* (*qsk1-1* and *qsk1-2*) published so far. We used *qsk1-1* (SALK_019840) in our study. As suggested, we got another allele, *qsk1-2* (GK-689A02) which has T-DNA insertion in the promoter region. As shown in the figure below, we genotyped and got the homozygous lines, and checked the transcript levels of *QSK1* by using two different sets of primers. However, unfortunately, *QSK1* transcript level was not reduced but rather increased in *qsk1-2* mutant, while *QSK1* transcript level was clearly reduced in *qsk1-1* mutant. Thus, we used only *qsk1-1* mutant in this study. Although we have one KO line, *qsk1-1/pQSK1:QSK1-GFP* complements the *qsk1-1* mutant phenotype, and *p35S:QSK1-3xHA#2 line* shows the opposite phenotype to *qsk1-1* mutant.

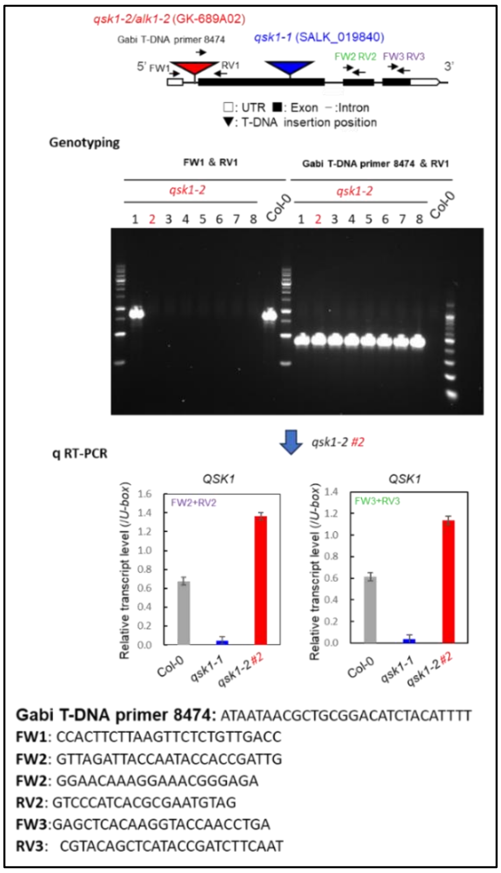

10. Protein sizes should be included in each WB images.
→ Thank you for pointing this out. I added the size of the marker in each WB image.

Dear Ken / Dr. Shirasu:

We have received reviews of your manuscript entitled "The leucine-rich repeat receptor kinase QSK1 is a novel regulator of PRR-RBOHD complex and is employed by the bacterial effector HopF2Pto to modulate plant immunity." On the basis of the advice received, the board of reviewing editors would like to accept your manuscript for publication in The Plant Cell. This acceptance is contingent on revision based on the comments of our reviewers. In particular, please consider addressing the points raised by reviewer 3 through textual changes.

Please highlight all changes and include a detailed annotation of changes of the text, with line numbers, and noting your responses to the comments.

To submit your revised manuscript, click:

Link Not Available

If you have any questions about the revision submission procedures, please contact the Editorial Office Staff (tpc-submissions@aspb.org). If you cannot return the revised manuscript within 30 days, please let us know. Otherwise, we will assume that you have elected not to revise the manuscript and withdraw it.

Thank you very much for the privilege of reviewing this work. I look forward to receiving the next version.

Sincerely,

The Plant Editorial Board

Reviewer #1 (Comments for the Author):

The authors have addressed all my previous comments.

Reviewer #2 (Comments for the Author):

We believe that the revisited manuscript adequately answers the questions and is worthy of publication.

Reviewer #3 (Comments for the Author):

In this study, Yukihisa Goto and colleagues identified a novel regulator of the PRR-RBOHD complex, which plays a role in plant immunity and contributes to the PTI co-receptor scheme that enables interaction between pathogens and plants. This finding is of interest and value in the context of understanding plant PTI. The revised manuscript is well written and shows clear evidence of improvement. I recommend that the manuscript be published, although I have a few suggestions for improvements.

1. As illustrated in Fig. S11B, the authors demonstrated that the FLS2 protein level decreased following a one-hour flg22 treatment. Conversely, overexpression of QSK1 was observed to inhibit the expression of FLS2. However, no discernible difference was observed between lanes three and four. A similar observation can be made with regard to the expression of the ERF protein in Fig. S11C.

2. The accumulation of QSK1 transcript induced by flg22 and elf18 is clear in Fig. S8. However, these PAMPs also induce the PTI response. It would be beneficial to discuss in more detail why a negative modulator accumulates during infection.

3. In Fig. S4A, it would be helpful to identify which band is the target band in the western blot, which is marked with two asterisks.

Please also note the following:

-The Plant Cell now requires authors to complete and submit an author revisions checklist upon submission of a revised manuscript. The aim of the checklist is to aid authors in preparing a high-quality manuscript, facilitate the review and assessment of revised manuscripts, and help to ensure that journal standards are maintained across the board. If your manuscript is accepted, the completed checklist will be published as supplemental material attached to the article online. Please download a copy of the checklist (pdf fillable form) at this link, for submission with your revised manuscript: https://tpc.msubmit.net/html/Author_Revisions_Checklist.pdf.

-Supplemental materials should be restricted to large datasets and tables, presentation of replicates, and validation of reagents, methods, or genotypes. Any data that are used to support the major claims must be in the main manuscript. Supplemental figure legends must indicate what figure in the main manuscript is supported by the supplemental data presented. Please justify how each of the supplemental figures meet the criteria.

-Sampling methods and the nature of "biological replicates" should be described precisely (i.e. different plants, parts of plants, pooled tissue, independent pools of tissue, sampled at different times, etc.), along with a clear description of and rationale for any statistical analyses conducted. The reader should know exactly what was sampled; what forms the basis of the calculation of any means and statistical parameters reported. This is also necessary to ensure that proper statistical analysis was conducted.

Dear Prof. Xing Li, Senior Editor

Dear Prof. Blake Meyers, Editor-in-Chief, The Plant Cell

We would like to express our sincere appreciation to you and the reviewers for your valuable time and insightful feedback on our manuscript.

In response to the suggestions provided by Reviewer 3, we have made the following revisions:

1. As illustrated in Fig. S11B, the authors demonstrated that the FLS2 protein level decreased following a one-hour flg22 treatment. Conversely, overexpression of QSK1 was observed to inhibit the expression of FLS2. However, no discernible difference was observed between lanes three and four. A similar observation can be made with regard to the expression of the ERF protein in Fig. S11C.

The reviewer noted that overexpression of QSK1 reduced FLS2 abundance and did not further reduce FLS2 and EFR protein abundance after recognition of PAMPs (no discernible difference was observed between lanes three and four). We agree with this observation and added a discussion highlighting that in *p35S:QSK1-3xHA#2,* a portion of the FLS2 and EFR proteins may be non-functional or mislocalized, preventing their recognition of PAMPs, subsequent endocytosis, and degradation following PAMP recognition (lines 283-285).

2. The accumulation of QSK1 transcript induced by flg22 and elf18 is clear in Fig. S8. However, these PAMPs also induce the PTI response. It would be beneficial to discuss in more detail why a negative modulator accumulates during infection.

As suggested by reviewer 3, We added discussion in lines 569-571. “Additionally, QSK1-mediated negative regulation might be upregulated following PAMP recognition, as PAMPs increase QSK1 transcript levels (Supplemental Fig. S8), suggesting that QSK1 may contribute to the shutdown of PTI after its activation.”

3. In Fig. S4A, it would be helpful to identify which band is the target band in the western blot, which is marked with two asterisks.

As suggested by reviewer 3, I modified Fig.S4A and added more detailed explanations in the legend.

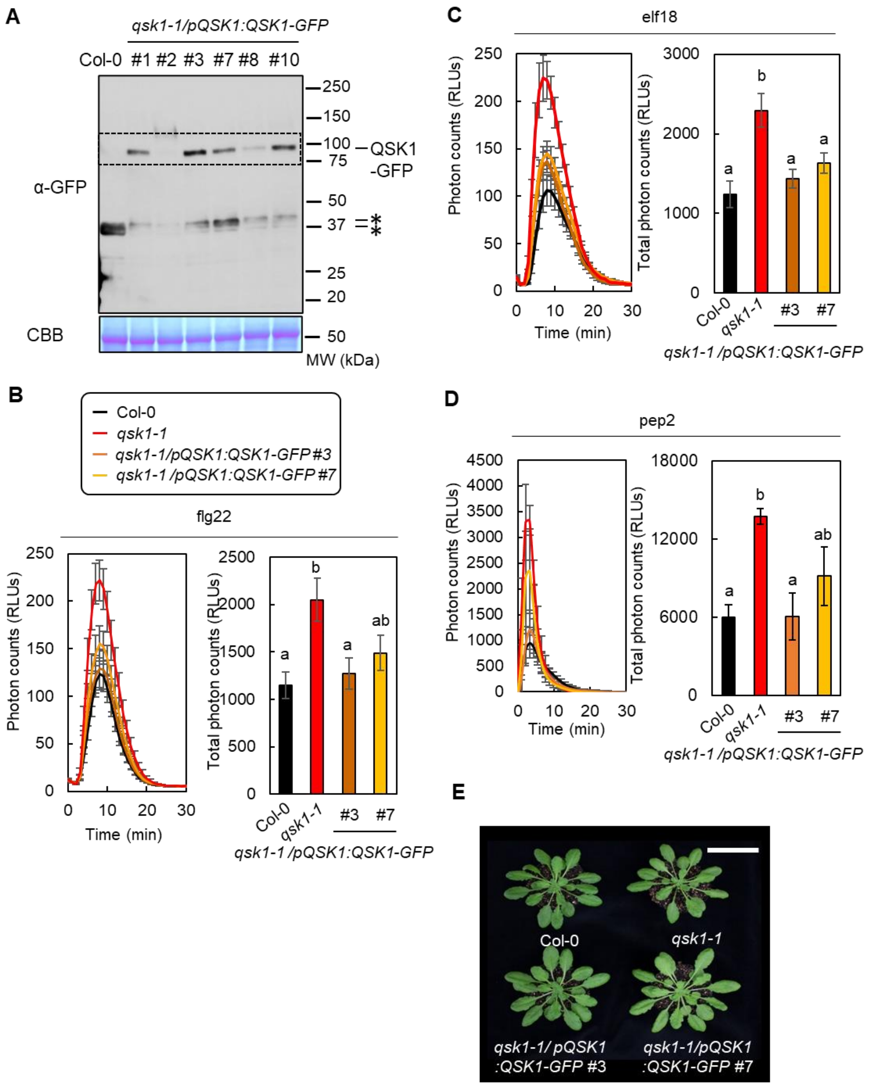

Supplemental Figure S4. Phenotype recovery in *qsk1* complementation lines. (Supports Figure 3) A) The QSK1-GFP protein levels in two-week-old seedlings of *qsk1-1/pQSK1:QSK1-GFP* lines were measured by immunoblotting with α-GFP antibody. Equal loading of protein samples is shown by CBB staining. Asterisks indicate non-specific bands present also in Col-0. **B-D)** *qsk1-1/pQSK1:QSK1-GFP* lines induce a similar level of ROS production upon treatment with flg22 (**B**), elf18 (**C**), and pep2 (**D**) to Col-0. 16 leaf discs from four- to five-week-old Arabidopsis plants were treated with 1 µM flg22, 1 µM elf18, or 1 µM pep2, and the time-course (left), and total amount (right) of ROS production were measured by a luminol-based assay. Values are mean ± SE (n=16). Different letters indicate significant differences at $p \leq 0.05$ (one-way ANOVA, Tukey's post hoc test). **E)** The growth phenotype of six-week-old Col-0, *qsk1-1 mutant*, and *qsk1-1/pQSK1:QSK1-GFP* lines. The white bar represents 5 cm. The experiments were repeated three times with similar results.

Dear Dr. Ken Shirasu:

We are pleased to inform you that your paper entitled "The leucine-rich repeat receptor kinase QSK1 is a novel regulator of PRR-RBOHD complex and is employed by the bacterial effector HopF2Pto to modulate plant immunity" has been accepted for publication in The Plant Cell, pending a final editorial review by a science editor. At this stage, your manuscript will be evaluated by a science editor with respect to its presentation of scientific content, compliance with journal policies, and presentation for a broad readership. The Plant Cell has contracted with Plant Editors (planteditors.com) to provide this service to our authors, and you will soon receive additional information on this process.

Please note that each author needs to link their ORCID identifier to their account in the system before your manuscript can be published. If any authors do not have an ORCID linked to their account, they will receive a message with a link to complete this task. Please ensure that ALL of your coauthors have completed this task as soon as possible.

LICENSE INFORMATION Shortly after your paper is sent to production, you will receive an email from our publisher's author support team, **SciPris**, with information on article processing charges, as well as the types of Open Access license you would be able to purchase. The accepted version of your manuscript will not appear on our Advance Articles page until the license is selected and signed, so please look out for an email from SciPris. For more information see the Article Publication Fees section of our author guidelines: https://academic.oup.com/plcell/pages/General-Instructions

The Plant Cell and The Arabidopsis Information Resource (TAIR) are collaborating to collect functional annotation data about Arabidopsis genes from authors. This includes information about the gene's molecular function (e.g., kinase activity, ATP synthetase activity), the biological process/es it is involved in (e.g., endosperm development, threonine biosynthesis), its subcellular location (e.g., nucleus, ER), anatomical or developmental expression pattern (e.g., leaf, ovule, flower stage 10, seedling stage), or its partner in a protein-protein interaction (e.g., AT1G01010 interacts with AT1G01020).

If your paper contains results falling into one or more of these categories for Arabidopsis genes, we request that you now submit these data for inclusion in TAIR by filling in the form provided at the following URL: https://www.arabidopsis.org/doc/submit/functional_annotation/123. If you need further clarification on what types of data can be submitted please contact curator@arabidopsis.org.

Finally, we encourage your submission of artwork for the journal cover. If there is an image or illustration related to your paper that you would like to have considered, please email it in .pdf or .tif format to clowe@aspb.org. An image title, brief, 2-3 sentence description of the image, and the name of the person credited for the image are also encouraged. If your image is selected to appear on the online cover of the journal, you will be contacted with the scheduled issue date and further information.

We look forward to seeing your paper published.

Sincerely,

The Plant Cell Editorial Board

=====
IMPORTANT REMINDER: PEER REVIEW REPORTS
=====

If you opted to publish a peer review report along with your article during the original submission process, it will be prepared by the editorial staff and publicly posted with your manuscript, inside the zip file that contains any other supplemental material. As a reminder, the peer review report is a public record of all comments from editors and reviewers, as well as your prior responses, as you received them in the decision letters for each draft of your manuscript. If you agreed to publish this report and have changed your mind, or are not sure if you selected this option, please contact the editorial office as soon as possible before signing the license agreement from our publisher.

=====

----FOR ASPB OFFICE USE ONLY (DO NOT EDIT)----
MSID: 40327

Corresponding Author: Prof. Shirasu
